# Remote management of heart failure using implantable electronic devices

**DOI:** 10.1093/eurheartj/ehx227

**Published:** 2017-05-27

**Authors:** John M. Morgan, Sue Kitt, Jas Gill, Janet M. McComb, Ghulam Andre Ng, James Raftery, Paul Roderick, Alison Seed, Simon G. Williams, Klaus K. Witte, David Jay Wright, Scott Harris, Martin R. Cowie

**Affiliations:** 1 Faculty of Medicine, University of Southampton, Tremona Road, Southampton SO16 6YD, UK; 2 Wessex Cardiology Centre, University Hospital Southampton NHS Foundation Trust, Southampton SO16 6YD, UK; 3 Department of Cardiology, Guy’s and St Thomas’ NHS Foundation Trust, London SE1 9RT, UK; 4 Department of Cardiology, Newcastle-upon-Tyne Hospitals NHS Foundation Trust, Newcastle-upon-Tyne NE7 7DN, UK; 5 NIHR Leicester Cardiovascular Biomedical Research Unit, University of Leicester, Glenfield Hospital, Leicester LE3 9QP, UK; 6 Department of Cardiology, Blackpool Teaching Hospitals NHS Foundation Trust, Blackpool FY3 8NR, UK; 7 Department of Cardiology, University Hospitals of South Manchester NHS Foundation Trust, Manchester M13 9WL, UK; 8 Multidisciplinary Cardiovascular Research Centre, University of Leeds, Leeds, UK; 9 Institute of Cardiovascular Medicine and Science, Liverpool Heart and Chest Hospital NHS Foundation Trust, Liverpool L14 3PE, UK; 10 Imperial College London (Royal Brompton Hospital), Dovehouse Street, London SW3 6LY, UK

**Keywords:** Remote monitoring, Heart failure, Implantable devices

## Abstract

**Aims:**

Remote management of heart failure using implantable electronic devices (REM-HF) aimed to assess the clinical and cost-effectiveness of remote monitoring (RM) of heart failure in patients with cardiac implanted electronic devices (CIEDs).

**Methods and results:**

Between 29 September 2011 and 31 March 2014, we randomly assigned 1650 patients with heart failure and a CIED to active RM or usual care (UC). The active RM pathway included formalized remote follow-up protocols, and UC was standard practice in nine recruiting centres in England. The primary endpoint in the time to event analysis was the 1st event of death from any cause or unplanned hospitalization for cardiovascular reasons. Secondary endpoints included death from any cause, death from cardiovascular reasons, death from cardiovascular reasons and unplanned cardiovascular hospitalization, unplanned cardiovascular hospitalization, and unplanned hospitalization. REM-HF is registered with ISRCTN (96536028). The mean age of the population was 70 years (range 23–98); 86% were male. Patients were followed for a median of 2.8 years (range 0–4.3 years) completing on 31 January 2016. Patient adherence was high with a drop out of 4.3% over the course of the study. The incidence of the primary endpoint did not differ significantly between active RM and UC groups, which occurred in 42.4 and 40.8% of patients, respectively [hazard ratio 1.01; 95% confidence interval (CI) 0.87–1.18; *P* = 0.87]. There were no significant differences between the two groups with respect to any of the secondary endpoints or the time to the primary endpoint components.

**Conclusion:**

Among patients with heart failure and a CIED, RM using weekly downloads and a formalized follow up approach does not improve outcomes.

## Introduction

Despite advances in the care of heart failure patients they remain at high risk of death and hospitalization.^1–3^ Studies that have investigated a range of remote monitoring (RM) strategies, intended to help patients avoiding hospitalization due to heart failure deterioration, have shown mixed outcomes with little evidence of significant clinical benefit. It is likely that the effectiveness (and cost-effectiveness) of RM depends on the design of care pathways in which the technologies are deployed.^4^

We conducted a multicentre, randomized, controlled trial, remote management of heart failure using implantable electronic devices (REM-HF), to determine whether management of heart failure patients in response to information gained by RM of implanted devices would reduce the combined endpoint of death from any cause and hospitalization for cardiovascular reasons, as compared to usual care (UC). To make the study as relevant as possible to real-world clinical practice and circumstances, it was conducted using devices from multiple manufacturers. The RM follow-up processes were formalized according to the prevailing understanding of clinical practice at the time of study inception and allied healthcare professionals were trained in the RM process in each of the participating centres. To our knowledge, REM-HF is the largest study, with longest follow-up, of RM of heart failure to date.

## Methods

### Study design, administration, and supervision

A description of the study design was published previously.^5^ In summary, our study was a randomized, event-driven, multicentre, open label, and parallel group clinical trial in which patients with heart failure and a cardiac implanted electronic device (CIED) [cardiac resynchronization therapy (CRT) with pacemaker function (CRT-P), CRT with defibrillator function (CRT-D), or implantable cardioverter-defibrillator (ICD)] were randomized to receive either UC or management informed by active remote-monitoring (RM). The active RM approach is described in detail below. In summary, it consisted of weekly data downloads from patients’ devices with simultaneous review by remote monitors who followed a defined active follow-up approach. Usual care was existing follow-up practice in the participating centres and care was taken that it was not influenced by study procedures. The context of the study was the UK’s National Health Service and nine recruiting centres.

The study protocol (which is available at http://eprints.soton.ac.uk/398865/) was approved by the National Research Ethics Service Committee, Yorkshire and the Humber-Sheffield, UK. The study was registered with the UK’s Clinical Research Network (10383) and ISRCTN (96536028) and conducted in accordance with the Good Clinical Practice guidelines and the principles of the 2002 Declaration of Helsinki. All patients recruited to the study provided written informed consent.

The Steering Committee oversaw the execution of the trial and data analysis according to a pre-specified statistical analysis. A Supervisory Committee was responsible for trial governance. An independent Endpoint Review Committee reviewed all patient events and adjudicated them according to its charter, detailed in the statistical analysis plan. The trial was reviewed by an independent Data and Safety Monitoring Committee who reviewed an interim analysis for signals of harm, overwhelming benefit, or futility, and sanctioned study continuation when 400 primary events had been adjudicated on 27 February 2015.

### Inclusion and exclusion criteria and randomization procedure

For inclusion, patients had to have symptomatic heart failure (NYHA Class II–IV) documented at enrolment, with a CIED (ICD;CRT-D; CRT-P) implanted according to NICE guidance or local clinical discretion at least 6 months previously (and optimally programmed according to the treating physician), stable and optimal medical therapy (working to NICE Guidelines) for heart failure for 6 weeks prior to enrolment, the ability to independently comprehend and complete quality of life questionnaires and to give informed consent. Patients were not eligible for recruitment if they had had any device change or lead replacement procedure within 30 days, had had an acute myocardial infarction or any cardiac surgical procedure within 3 months, were unable to use the technology due to mental or physical limitations, were aged <18 years, were pregnant, were on a planned heart transplantation list, had a life expectancy of less than a year due to non-cardiovascular disease, had current CIED complications (such as wound infection, haematoma, lead fracture), or were unable to understand written and spoken English.

Patients meeting these criteria, after informed consent, were randomized 1:1 to UC or to active RM, performed centrally by FormsVision B.V. (Abcoude, The Netherlands) via an electronic care record form management system. The randomization schedule was stratified by recruiting site and device type (CRT-P; CRT-D; ICD), with randomly permuted block sizes of either four or 6 patients.

### Remote monitoring care pathway, procedural handbook, and remote monitoring staff

The trial investigators adopted a pragmatic approach to active RM based on a feasibility study,^5^ steering committee clinical experience, and reference to the published literature. The RM attributes of CIEDs differ between manufacturers and the parameters monitored by manufacturers’ CIEDs are listed in *Table 1*. Remote monitoring clinical management procedures were standardized across centres and formalized in a Procedural Handbook (Appendix II to the protocol) that guided reactions to active RM changes (including medication changes, lifestyle advice, and onward referral). The Procedural Handbook integrated the monitored parameters into the active RM workflow. Choice of CIED was determined by purchasing practice in participating centres.

**Table 1:** Parameters measured by cardiac implanted electronic devices for remote monitoring and used to guide interventions

Medtronic	Boston scientific	St Jude medical
Bi-ventricular pacing %	Bi-ventricular pacing %	Bi-ventricular pacing %
Nocturnal HR		
Thoracic Impedance		Thoracic impedance (if programmed on)
Activity levels	Activity levels	Activity levels
AT/AF burden	AT/AF burden	AT/AF burden
Ventricular arrhythmias	Ventricular arrhythmias	Ventricular arrhythmias
Therapy from device	Therapy from device	Therapy from device
Heart rate variability	Heart rate variability (SDANN)	
Lead integrity	Lead integrity	Lead integrity
Device programming	Device programming	Device programming
V–V interval at time of D/L	V–V interval at time of D/L	V–V interval at time of D/L

HR, hazard ratio; AT, atrial tachycardia; AF, atrial fibrillation.

At each of the nine study sites, one healthcare professional (nurse practitioner or clinical physiologist experienced in heart failure management and/or CIED follow-up) was appointed as the monitor responsible for screening and enrolling patients, active RM, and study management. These monitors underwent an initial 2 days intensive training in application of the CIED’s monitoring technologies in the study protocol, the workflows described in the Procedural Handbook (which was written by the steering committee to optimize monitoring capabilities based on understanding of the literature, personal experience, and learnings from a the feasibility study described in a methodology article^5^) and active RM aims. There was subsequent informal refresher training at intervals during the study.

Following patient enrolment, they instructed participants randomized to active RM in how to perform weekly CIED downloads. They also interpreted the weekly downloads with particular attention to the observation of multi-parameter trends rather than single observations (including thoracic impedance change) and in the context of patient feedback during telephone interviews. No ‘alerts’ were programmed ‘on’ for the purpose of heart failure management and therefore, there was no co-ordinated heart failure care response to ‘alert’ generation. ‘Alerts’ for lead integrity or battery depletion were allowed according to physician discretion. The remote monitors offered lifestyle, clinical and medication change advice to patients within the confines of the Procedural Handbook, and additional clinic visits, or recommendation to attend their general practitioner or the emergency room. Patients in the UC group were informed of study follow-up procedures. The remote monitor contacted these patients by telephone to make a comprehensive assessment of heart failure events during the study follow-up period, with completion of the study UC CRF. However, the remote monitor was instructed not to interfere with UC patient management and to avoid any patient interactions that might inadvertently do so. For UC, those sites that used RM for device checks continued that practice as to do otherwise would have interfered with UC. At its most frequent, this was done every 6 months and without use of a ‘protocol-driven’ response. Usual care did not include use of active RM to manage heart failure in any form.

### Data collection and management

All study data for both the active RM and UC groups were collected and recorded by the site staff on the electronic case record form, which captured clinical events and healthcare utilization elements.

### Follow-up timing

Contact was made with all patients at 3, 6, 12, and 24 months and at the end of the study.

Continual on-site monitoring was performed with source-data verification of core data in all patients. Central monitoring of documents (patients’ records and electronic case report forms) regarding serious adverse events was performed before assessment by the endpoint review committee. The trial was terminated after the protocol-specified goal of 546 identified and adjudicated primary endpoints was met, and patients exited from the study with an end-of-trial telephone contact so that any remaining endpoints or adverse events could be collected before the database was locked.

### End points

The primary study endpoint in the time-to-event analysis was the 1st event of the composite of death from any cause or an unplanned hospitalization for cardiovascular reasons. The secondary endpoints were death from any cause; cardiovascular death; non-cardiovascular death; cardiovascular-related death or unplanned cardiovascular hospitalization; death from any cause or unplanned hospitalization for non-cardiovascular reason; unplanned cardiovascular hospitalization; unplanned hospitalization for non-cardiovascular reasons.

### Statistical analysis

The study was designed to show a maximum hazard ratio (HR) of 0.755 in the rate of the 1st primary end-point event with RM. We calculated that data on 546 events were required for the study to have power of 90% to show that reduction, at an overall two-sided type I error rate of 5%. We estimated that this would require a minimum of 697 patients to be recruited per group (1394 total) with a minimum follow-up of 2 years, but to allow for patient drop-out, we increased the total planned recruitment to 1650.

The primary analysis was conducted in the intention to treat population, which consisted of all randomized subjects, with each subject analysed as part of the group to which they were randomized and including all events that occurred before the database was locked. This included any subjects later found to be ineligible and those who did not follow the trial protocol. The trial was designed to include one interim analysis and used the Peto–Haybittle rule to maintain an overall 5% type I error. The significance level of the final analysis was 0.048. Cox proportional hazards models including the stratification variables of recruiting site and device type were used to test for differences between the UC and active RM groups. The excess of events at the termination of the trial was included in the final analyses. Cause-specific HR was calculated, and cumulative incidence curves were used to visualize survival data. Further details regarding the statistical analysis, including analyses of the secondary endpoints and a priori subgroup analyses, are provided in the statistical analysis plan (Appendix III to the protocol).

## Manuscript preparation

The study funders and sponsor had no role in study design, collection, analysis, and interpretation of data, manuscript preparation or decision to submit the article for publication. The 1st draft of the manuscript was prepared by the study principal investigators (JMM and MRC), who, with the trial statistician, had unrestricted access to the data. The manuscript was reviewed and edited by all the authors. All the authors made the decision to submit the manuscript for publication and assume responsibilities for the accuracy and completeness of the analyses and for the fidelity of this report to the trial protocol.

## Results

**Figure 1:**
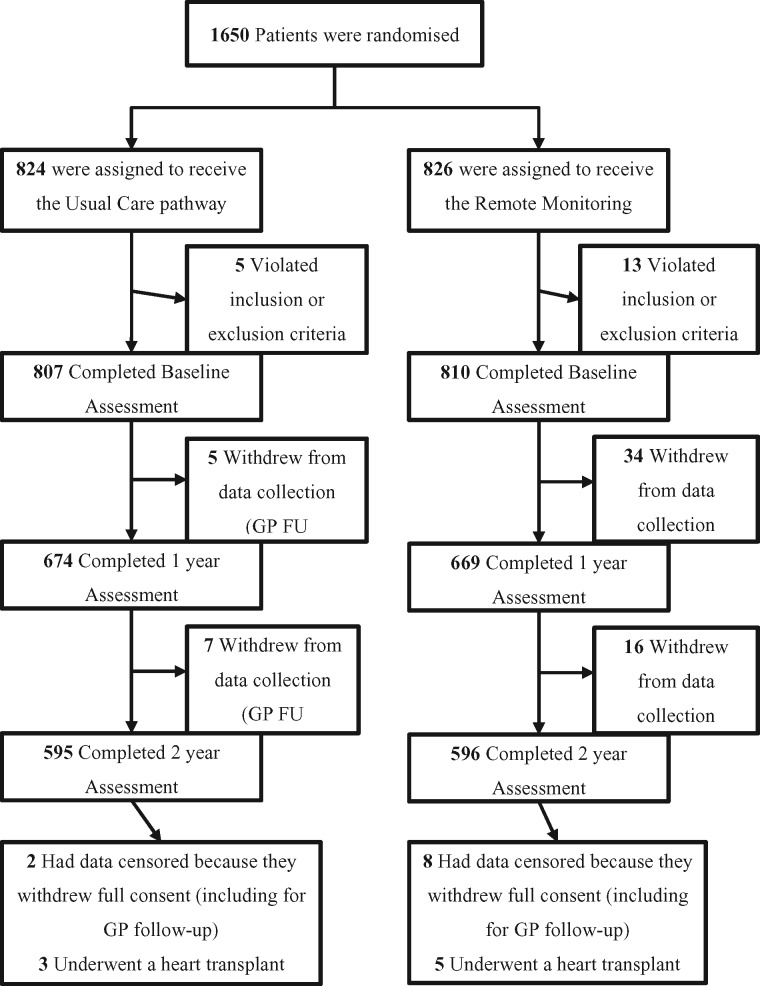
Randomization, treatment, and follow-up of the patients. GP, general practitioner doctor.

Between 29 September 2011 and 31 March 2014 1650 patients were enrolled and included in the intention-to-treat analysis; 824 patients were assigned to the UC group and 826 to the RM group (*Figure 1*). Vital-status verification was completed for 100% of patients. Consent for further follow-up was withdrawn by 72 (4·4%) patients during the study (mainly due to inclusion in other heart failure therapy studies). Baseline characteristics of the patients were similar between the two groups (*Table 2*). The mean age was 70 years; 86% were male. The majority of patients were in New York Heart Association (NYHA) Classification II (70%) or III (30%). At baseline, 91% were taking an angiotensin converting enzyme inhibitor or angiotensin receptor blocker, 91% were taking a beta-blocker, and 52% an aldosterone antagonist.

**Table 2:** Demographic and clinical characteristics of the patients at baseline, according to treatment group^a^

Characteristic		Randomized group		
		Remote monitoring *n* = 824	Usual care *n* = 826	Total *n* = 1650
Age (years)		69.5 ± 10.31	69.5 ± 10.04	69.5 ± 10.17
Age (years)—Females		66.5 ± 11.90	68.00 ± 11.47	67.2 ± 11.69
Age (years)—Males		69.9 ± 9.95	69.8 ± 9.76	69.9 ± 9.85
Male sex		707 (85.8%)	708 (85.7%)	1415 (85.8%)
BMI		28.9 (5.72)	28.8 (5.48)	28.9 (5.60)
BMI—Females		29.0 (6.55)	28.1 (6.34)	28.6 (6.45)
BMI—Males		28.9 (5.58)	28.9 (5.32)	28.9 (5.45)
Recruiting Site	Blackpool	98 (11.9%)	102 (12.4%)	200 (12.1%)
	Brompton	85 (10.3%)	84 (10.2%)	169 (10.2%)
	Guys	64 (7.8%)	65 (7.9%)	129 (7.8%)
	Leeds	102 (12.4%)	99 (12.0%)	201 (12.2%)
	Leicester	102 (12.4%)	100 (12.1%)	202 (12.2%)
	Liverpool	98 (11.9%)	105 (12.7%)	203 (12.3%)
	Manchester	101 (12.3%)	99 (12.0%)	200 (12.1%)
	Newcastle	95 (11.5%)	93 (11.3%)	188 (11.4%)
	Southampton	79 (9.6%)	79 (9.6%)	158 (9.6%)
NYHA Classification	II	585 (71.0%)	561 (67.9%)	1146 (69.5%)
	III	238 (28.9%)	263 (31.8%)	501 (30.4%)
	IV	1 (0.1%)	2 (0.2%)	3 (0.2%)
Myocardial infarction		497/818 (60.1%)	478/818 (58.4%)	975/1636 (59.6%)
Coronary artery bypass surgery		268/819 (32.7%)	247/818 (30.2%)	515/1637 (31.5%)
Percutaneous coronary suregery		209/819 (25.5%)	190/818 (23.2%)	399/1637 (24.4%)
Valve replacement		66/819 (8.1%)	51/819 (6.2%)	117/1638 (7.1%)
Diabetes mellitus	Type I	5/819 (0.6%)	9/819 (1.1%)	14/1638 (0.9%)
	Type II	203/819 (24.8%)	216/819 (26.4%)	419/1638 (25.6%)
Type II diabetics on medication		155 (76.4%)	171 (79.9%)	369 (80.2%)
Systolic blood pressure (mmHg)		118.9 ± 17.61	119.1 ± 18.43	119.0 ± 18.02
Diastolic blood pressure (mmHg)		70.1 ± 11.02	70.1 ± 11.33	70.1 ± 11.17
Type of cardiac implantable device	ICD	275 (33.4%)	276 (33.4%)	551 (33.4%)
	CRT-D	442 (53.6%)	438 (53.0%)	880 (53.3%)
	CRT-P	107 (13.0%)	112 (13.6%)	219 (13.3%)
Pulse (beats/minute)		68.1 (10.22)	68.6 (9.92)	68.3 (10.07)
History of atrial fibrillation		339/819 (41.4%)	338/816 (41.4%)	677/1635 (41.4%)
Haemoglobin (g/L)		134.6 ± 15.57	133.1 ± 16.22	133.8 ± 15.91
Documented coronary artery disease		563/819 (68.7%)	548/818 (67.0%)	1111/1637 (67.9%)
Left ventricular ejection fraction (%)		29.9 ± 10.24	30.0 ± 9.81	29.9 ± 10.02
Smoking history	Never smoked	275 (33.6%)	262 (32.0%)	537 (32.8%)
	Ex-smoker	471 (57.5%)	495 (60.4%)	966 (59.0%)
	Current smoker	73 (8.9%)	62 (7.6%)	135 (8.2%)
Oral anticoagulant		394 (47.8%)	389 (47.1%)	783 (47.5%)
ACE inhibitor or ARB		750 (91.0%)	754 (91.3%)	1504 (91.2%)
Beta-blocker		749 (90.9%)	746 (90.3%)	1495 (90.6%)
Aldosterone antagonist		430 (52.2%)	435 (52.7%)	865 (52.4%)
Diuretic (excluding aldosterone antagonists)		635 (77.1%)	631 (76.4%)	1266 (76.7%)
Antiplatelet		485 (58.9%)	448 (54.2%)	933 (56.5%)
Cardiac glycoside		153 (18.6%)	180 (21.8%)	333 (20.2%)
Anti-arrhythmic		205 (24.9%)	203 (24.6%)	408 (24.7%)

ACE, angiotensin-converting enzyme; ARB, angiotensin-receptor blocker; COPD, chronic obstructive pulmonary disease; NYHA, New York Heart Association; ICD, implantable cardioverter defibrillator; CRT-D, cardiac resynchronization therapy with defibrillator function; CRT-P, cardiac resynchronization therapy with pacemaker function; BMI, body mass index.

a All comparisons between the two groups were non-significant (*P* > 0.05).

At 6 months after randomization, 476 patients in the RM group (58% of the 824 patients randomized) had transmitted data for at least 75% of weeks in the study (not including any periods of hospitalization). This had increased to 548 patients (66%) by 12 months, and was 513 (62%) at 24 months. A total of 79 325 CIED downloads were reviewed in the RM group, and action was taken in 599 (73%) of patients: in 131 of these patients (22%) action was taken in response to only one download, in a further 201 patients (34%) action was taken in response to two–four downloads, and 267 patients (45%) had action taken in response to five or more downloads (maximum of 38). The actions taken, and the number of patients affected, are shown in *Table 3*. The numbers quoted are number of participants affected. Some participants had multiple actions with a total of 5536 actions taken on 3534 occasions (multiple actions taken at some points).

**Table 3:** Actions taken in response to weekly remote download of cardiac implanted electronic devices data (remote monitoring group)

Action Taken	Number of subjects impacted (Number of Incidences) {% of patients}
Phoned Patient	520 (2378) {63.0}
Mean per patient-year	1.15
Median per patient-year (LQ–UQ)	0.43 (0–1.45)
Discussed download with clinician	408 (1390) {49.4%}
Mean per patient-year	0.68
Median per patient-year (LQ–UQ)	0 (0–0.85)
Medication change by remote monitor	134 (226) {16.2}
Mean per patient-year	0.11
Median per patient-year (LQ–UQ)	0 (0–0)
Advised to contact GP or attend a clinic Mean per patient-year	345 (910) {41.8}0.45
Median per patient-year (LQ–UQ)	0 (0–0.53)
Advised to contact GP	124 (206) {15.0}
Advised to visit HF clinic	113 (198) {13.7}
Advised to attend device clinic	202 (328) {24.5}
Advised to attend cardiovascular outpatient clinic	109 (178) {21.5}
Other advice to patient	274 (632) {(33.2}
Mean per patient-year	0.30
Median per patient-year (LQ–UQ)	0 (0–0.30)
Total	599 (5536) {72.6}
Mean per patient-year	2.68
Median per patient-year (LQ–UQ)	1.03 (0–3.36)

GP, general practitioner doctor; HF, heart failure; LQ, lower quartile; UQ, upper quartile.

**Figure 2:**
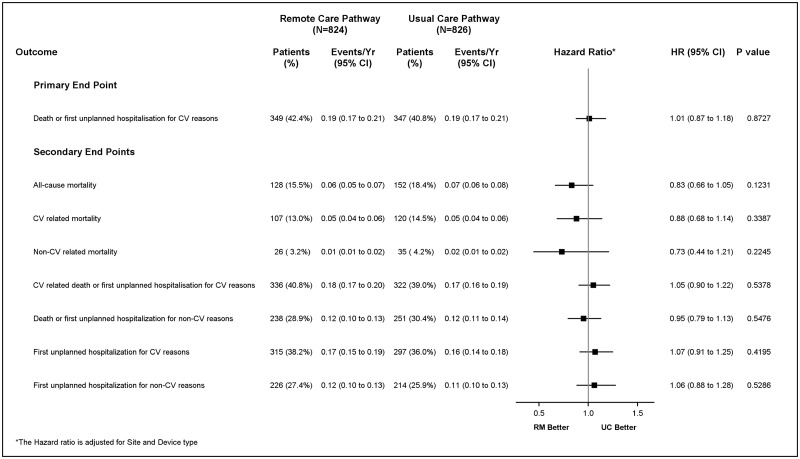
Forest plot of the comparison of end point events by treatment group.

**Figure 3:**
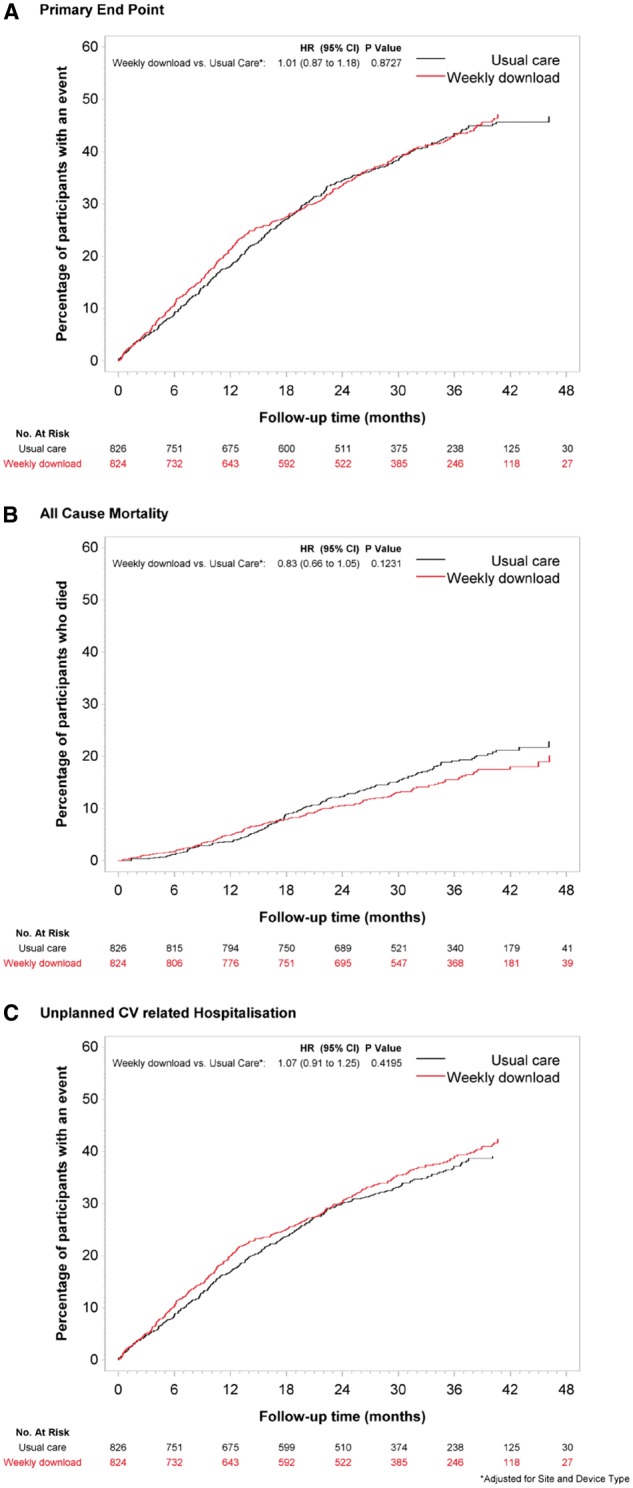
Cumulative incidence curves for the primary end point, death from any cause, and unplanned hospitalization for cardiovascular reasons. The primary endpoint was a composite of death from any cause and unplanned hospitalization for cardiovascular reasons.

Patients were followed for a median of 2.8 years (range 0–4.3 years). A summary of endpoint events is provided in *Figure 2*. No significant difference was seen in the rate of the primary end point, which occurred in 349 patients (42.4%) in the RM group and in 347 patients (40·8%) in the UC group (HR 1·01; 95% confidence interval [CI] 0·87 to 1·18; *P* = 0·87). Cumulative incidence curves for the composite primary endpoint of death from any cause and hospitalization for cardiovascular reasons, as well as for both components separately, did not reveal a significant difference between the two groups (*Figure 3*). No significant differences were seen between the two groups with respect to the secondary endpoints (*Figure 2*).

Subgroup analyses showed that none of the baseline characteristics—including age (<70 years vs. ≥70 years), gender, NYHA class (Class I or II vs. III or IV), type of device, documented history of coronary artery disease, or history of atrial fibrillation, identified a group in which management guided by RM was more effective than UC. No adverse events were reported during the study period.

## Discussion

Active RM studies for heart failure have used a wide variety of technologies and clinical care approaches.^6–11^ The importance and relevance of remote device monitoring has been considered in recent European Society of Cardiology guidelines.^12^ In its simplest form, active RM has used telephone-based patient support. Greater monitoring capability is afforded by devices that collect data such as thoracic impedance, cardiac rhythm change, haemodynamics, and patient activity. Meta-analyses of small studies suggested that this type of active RM may reduce death from heart failure and hospitalization.^13^^,^^14^ More recently, however, the telemonitoring to improve heart failure outcomes (Tele-HF) Trial,^7^ the telemedical interventional monitoring in heart failure (TIM-HF),^8^ and the Better Effectiveness After Transition-Heart Failure (BEAT-HF) Trial^9^ which tested non-implantable RM strategies against UC, demonstrated no effect on outcomes including death or hospitalization. However, Tele-HF and BEAT-HF only followed patients up to 6 months and reported only moderate patient compliance.^7^^,^^9^

More sophisticated monitoring technologies reside within CIEDs, which are implanted for therapeutic reasons. Diagnostic capabilities intended to signal heart failure deterioration include measurement of intrathoracic impedance, heart rate variability, nocturnal heart rate, patient activity, and occurrence of atrial and ventricular arrhythmia. A further design feature of CIEDs has been used of automated ‘alerts’ when monitored parameters exceed pre-determined thresholds considered to indicate heart failure deterioration.^10^^,^^15^^,^^16^ Their use, to signal heart failure status change and trigger a therapy change, has been a key element of the care pathway design in many RM studies, mainly based on intrathoracic impedance measurement. However, intrathoracic impedance has poor sensitivity and specificity for such deterioration and has been shown to drive unnecessary hospitalizations.^15^ In the Optimization of Heart Failure Trial, intrathoracic impedance monitoring using ‘alerts’ was not superior to routine care in avoiding death or hospitalization related to cardiovascular causes.^17^ In the Clinical Evaluation of Remote Notification to Reduce Time to Clinical Decision Trial (CONNECT) hospitalizations’ duration was reduced in those randomized to remote notification, driven chiefly by detection of atrial tachyarrhythmia lasting longer than 12 h but there was no reduction in the risk of hospitalizations.^11^

In contrast to single parameter, ‘alert-based’ monitoring approaches observational data of a combined multiparameter heart failure diagnostic algorithm suggested a 30-day window of identifying patients at higher risk of hospitalization for heart failure.^18^^,^^19^ The Implant-based Multiparameter Telemonitoring of Patients with Heart Failure (IN-TIME) study tested CIED monitoring of tachyarrhythmia, sub-optimal biventricular pacing, increased ventricular extrasystolic activity, and decreased patient activity with daily data downloads sent to a single monitoring centre.^20^ Comparing this strategy with UC for 12 months in 664 patients across 36 centres there was an improvement in a combined end-point of death from any cause, NYHA Class change and patient global self-assessment. The study demonstrated reduced mortality in the monitored group, although this effect may have been expressed principally in patients with atrial fibrillation.

Most recently, monitoring resynchronization devices and cardiac patients (MORE-CARE) was stopped early because of slow enrolment, with 865 of a planned 1720 enrolled.^21^ At 24 months, follow-up in these patients enrolled within 8 weeks of an implant of a CRT-D device, RM using an ‘alert’ for intrathoracic impedance and atrial tachyarrhythmias, there was no difference in the primary endpoint of all-cause mortality or hospitalization for cardiovascular or device-related reasons.

Previous studies of CIED monitoring have used a single manufacturer’s devices, monitoring at a single centre (IN-TIME),^20^ and have been chiefly driven by ‘alerts’ (e.g. CONNECT,^11^ MORE-CARE^21^, OPTILINK^17^) which may occur too late in the course of a heart failure deterioration episode to allow pre-emptive therapy, even if appropriately generated.

The REM-HF study investigators endeavoured to create a RM strategy that is useable in real-world clinical practice. Each recruiting centre conducted RM ‘in house’ and with multiple manufacturers’ products reflecting commercial and organizational realties which do not easily allow the practise of RM across regional or national boundaries or with a single manufacturer’s technology. REM-HF did not use ‘alerts’ to trigger interactions but rather review of changes in trends over time in monitored parameters; the incidence and nature of RM actions in REM-HF (5536 interventions in 599 patients) appears comparable to that in IN-TIME (1225 actions in 280 patients). We would argue that the patients’ adherence to the RM weekly download schedule throughout the study was as good as was pragmatically possible, generating additional contact with the majority (72.5%) of patients. In our multicentre, randomized, controlled trial in patients with heart failure and a CIED, we found no reduction in the risk of death from any cause or hospitalization for cardiovascular reasons with management guided by weekly active RM, as compared to UC in nine English hospitals. Subgroup analyses failed to identify a group for which the intervention was effective, including those with a history of atrial fibrillation. This was despite both the trial having a larger number of patients and considerably longer follow-up than other similar studies, and clinically relevant patient adherence to weekly downloads driving many additional contacts with patients. Importantly, however, we did not show an increase in hospitalization for heart failure as has been the case with pre-set ‘alert’ based approaches to RM of CIED.^15^

The patient population recruited to REM-HF may have been less sick than that in IN-TIME and OPTILINK as suggested by a higher proportion of patients with mild symptoms (NYHA Class II). However, the mortality rates in these studies appear similar to that found in REM-HF. More patients gave a history of atrial fibrillation at study enrolment in REM-HF (41.4%) than in either OPTILINK (30·4%)^16^ or IN-TIME (25·4%).^20^

In contrast to the use of diagnostic features within therapeutically-indicated CIEDs, two monitoring-only cardiac implantable technologies have undergone clinical trial assessment.^22^^,^^23^ A pressure sensor mounted on an intravascular lead and connected to an implanted monitoring device giving continuous right ventricular pressure monitoring failed to achieve a significant reduction in hospitalizations.^22^ However, a wireless pulmonary artery haemodynamic monitor that allows a physician to adjust medication to achieve a target pulmonary artery pressure reported a 33% reduction in hospitalizations at 6 months compared to UC in the US healthcare system.^23^ It is unclear whether the effect relates to RM and/or to the specific haemodynamic targeting of drug therapy but it may be that managing patients to remain within a target zone reflecting optimal physiological status offers better outcomes.

In summary, REM-HF employed a rigorous active RM strategy, employing the monitoring capabilities of CIEDs. The strategy was relevant to real-word clinical practice and not focused on single manufacturer technology or single centre capability although it was implemented by trained ‘remote monitors’. Nevertheless, this active RM strategy provided no benefit over UC for patients with heart failure. Thus, this large multicentre trial with longer follow-up than previous studies does not support the routine use of management guided by weekly routine RM using a CIED. Perhaps an effect could be demonstrated in health care systems with less well developed UC or in a sicker heart failure population. Furthermore, future developments in RM technologies may enhance diagnostic and interventional capabilities but will require robust evaluation before broad clinical adoption.

## Supplementary material

Supplementary material is available at *European Heart Journal* online.

## Supplementary Material

Supplementary Data
