# Supplementary material for: Remote management of heart failure using implantable electronic devices
Source: Eur Heart J. 2017 May 27;38(30):2352–60. doi: 10.1093/eurheartj/ehx227 (PMC5837548; doi:10.1093/eurheartj/ehx227)
Supplement: Supplementary Data [file supplementary_material_protocol_procedural_handbook_sap_ehx227.docx]

**Protocol**

**REM-HF**

**REmote Monitoring an evaluation of implantable devices for management of Heart Failure Patients**

**Professor John M Morgan**

**University Hospital Southampton NHS Foundation Trust**

**Tremona Road**

**Southampton S016 6YD**

**Tel: 023 8079 4633**

**REmote Monitoring an evaluation of implantable devices for management of heart failure patients – REM-HF**

**Study Summary Table**

| Methodology | Randomised non-blinded study |
| --- | --- |
| Study Duration | 5 years |
| Study Centre(s) | 9 UK major cardiac centres implanting >/=200 ICDs/CRT-Ds per annum |
| Objectives | A HTA of remote management of heart failure using an implanted monitoring device |
| Number of Subjects | Event-driven, but likely 1650 (825 remotely monitored) |
| Diagnosis and Main Inclusion Criteria | Patients implanted with an Implantable Cardioverter Defibrillator/Cardiac Resynchronisation Therapy-Defibrillator/Pacemaker device that has heart failure monitoring capabilities as well the arrhythmia monitoring. Patients must be on stable medical therapy for 6 weeks prior to recruitment and have had a device implant for 6 months. |
| Study Product, Dose, Route, Regimen | Industry wide ICD or CRT-D/P models capable of fulfilling clinical requirements of the study. |
| Reference therapy | Usual care moving towards remote monitoring but using patient driven measures |
| Statistical  Methodology | Multicentre randomised controlled trial, primary outcome combined endpoint of mortality and cardiovascular hospitalisation using Cox proportional hazards regression |

**Southampton Admin Team:**

**Study Centre Admin Lead: Professor JM Morgan**

**Study Co-PIs: Professor M Cowie; Professor JM Morgan**

**Study Manager: Sue Kitt**

**Study Nurse Leads: Sarah Earles; Lisa Fletcher**

**Coordinator: Michelle Beveridge**

**Contents:**

**1 Introduction**

**2 Study Importance**

**3 Risk-Benefit Justification**

**4 Study Hypothesis**

**5 Study Objectives**

**6 Inclusion and Exclusion Criteria**

**7 Staffing of the Study and Study Grant Costs**:

**8 Recruitment and Randomisation**

**9 Study Details and Data Collection**

**10 Data Analysis**

**11 Cost Effectiveness**

**12 Training**

**13 Project timetable:**

**14 Data Storage**

**15 Database**

**16 Expected Value Of Results**

**17 Safety issues**

**18 Study Committees**

**19 Investigator responsibilities**

**20 Monitoring**

**21 Premature termination**

**22 Indemnity**

**23 Study Personnel and Brief Job Descriptions**

**24 Reference List**

**Appendices 1 Introduction**

**Abstract**

There is a pressing need to develop new ways of managing the rapidly increasing number of people living with heart failure in the UK. The epidemic of heart failure has arisen because of the rapidly ageing population and dramatic improvements in the survival from acute coronary disease and heart failure. Remote monitoring of heart failure using automated implanted devices and bespoke care pathways/protocols to give early warning of deterioration and facilitate pre-emptive action may be an important solution to the dual problem of increasing healthcare need but static (or reducing) healthcare resource. This study randomises a typical population of patients with heart failure who have implanted devices capable of being monitored remotely to either protocol-driven care informed by weekly remote monitoring, or to usual care. The study is powered to detect a clinically-meaningful reduction in all cause mortality and cardiovascular hospitalisation as the primary endpoint, with secondary endpoints that include cost effectiveness. The results of this study could potentially change NHS practice for many tens of thousands of patients with heart failure, facilitating an integration of modern technology with evidence-based treatment to optimise health gain and value for money in the health service.

**Statement of Originality**

This is a large UK multicentre randomised trial of implanted remote monitoring technology for patients with heart failure. The differentiating aspects of this study are: use of implantable devices for automated data collection in the context of protocol-driven care pathways, facilitating optimal management of heart failure patients with less need for face-to-face secondary or tertiary care contact, leverage of best use of modern technology in the NHS setting, with comprehensive assessment of clinical and cost effectiveness. To our knowledge no other study has addressed these issues in the context of a prospective randomised study powered to show the impact of this care approach on patient mortality, hospitalisation, and quality of life, together with a robust evaluation of cost effectiveness.

**Background**

Chronic Heart Failure (CHF) epidemiology: CHF is common and related hospitalisation and healthcare costs continue to rise. The British Heart Foundation (BHF) estimates 393,000 men and 314,000 women aged over 45 in the UK have heart failure with prevalence increasingly steeply in the elderly.^1^ With the ageing population and improving treatment of coronary artery disease, heart failure prevalence will continue to increase rapidly.^2^ CHF is associated with poor survival due to either sudden arrhythmic death or death due to pump failure.^3,4^  CHF is also associated with significant morbidity: the Heart of England Screening study showed one third of people with heart failure have dyspnoea that severely limits physical activity (occurring at rest, or upon minimal exertion - washing, dressing or walking from room to room).^5^ SF-36 scores are significantly lower than those in the general population, in all eight areas of quality of life assessment by that tool. Quality of life is poorer than in many other chronic conditions such as chronic lung disease, arthritis, or other cardiac conditions such as angina. Hospital episode statistics showed that in 2008/9 (the most recent year with published data) there were 106, 800 ‘finished consultant episodes’ for heart failure in England, with 90% of these related to emergency hospital admission, with a mean length of stay of 11.8 days, and a total of 740 700 bed days – this represents around 1% of all emergency medical admissions and 2% of inpatient bed days.^6^ The PENTAG Economic Model (NHS R&D HTA Programme 2006) estimated that 1.8% of total NHS budget was spent on heart failure management, with 68% of the spend being on acute deterioration admissions due to pulmonary oedema, arrhythmia, silent infarction, infection, or drug non-compliance/efficacy reduction. Reduction in heart failure admissions is therefore of paramount importance in decreasing overall healthcare costs.^6,8^ No large randomised studies have evaluated the clinical or economic impact of care pathway-specific remote monitoring for management of CHF.

Frequent pre-emptive remote monitoring by devices may enable early identification and treatment of pulmonary congestion and malignant arrhythmias (the two main mechanisms for mortality in Heart Failure), which could reduce heart failure morbidity and related hospitalisation.^9,10,11^

Early detection of deterioration is enabled by implanted technologies that telemeter their recorded data for assessment by clinicians using bespoke information technology and which may offer greater benefit and better patient experience than available external technologies that patients themselves need to administer.

There is now strong evidence from systematic reviews that remote (patient-driven) monitoring does reduce mortality. ^12,13^ A number of previous studies have evaluated the use of a remote (patient-driven) monitoring strategy in heart failure. A recent meta-analysis by Klersy et al. identified 20 randomised controlled trials (RCTs) (6,258 patients) and 12 cohort studies (2,354 patients).^13^ Thirteen studies evaluated a telephone-based remote monitoring approach and 21 a technology-based approach, involving home monitoring equipment in 17 and an implantable device in 2. In both RCTs and cohort studies remote monitoring was associated with a significantly lower number of deaths (RCTs: relative risk, RR, 0.83 p=0.006; cohort studies: RR: 0.53 CI, p<0.001) and hospitalisations (RCTs: RR: 0.93, p=0.030; cohort studies: RR: 0.52, p<0.001). Cohorts have significant larger effect sizes partly due to selection biases. Two very recent external monitoring trials not included in the above reviews (HOME-HF^15^ and HHH Study^16^) showed no reduction in death or hospitalisation but found that emergency hospitalisations were reduced substantially. With improved CHF services (community-based nurse lead heart failure services and hospital-based specialist services) the picture is evolving. Of the 21 previous studies to have employed a technology-based remote monitoring approach, 17 used home monitoring equipment to collect physiological data such as blood pressure and weight, and another 2 used phone calls with decision support systems. However, as decompensation may be due to fluid shift rather than fluid overload, the relationship between weight gain and clinical deterioration is not straightforward, and body weight may not be that sufficiently sensitive as a marker of decompensation.^13,15^ Quality of life, cost and patient acceptability of remote monitoring however, have been less frequently reported. Clark et al’s meta-analysis of remote monitoring trials found that of 14 RCTs included, only 6 reported quality of life data, 4 reported costings, and 4 patient acceptability.^14^

Implantable monitoring devices have been designed to measure many complex internal physiological variables. Adamson et al. investigated the use of an implantable monitor that provided continuous right ventricular (RV) haemodynamic data, in 32 patients with chronic heart failure.^17^ During the study there were 12 hospitalisations with decompensated heart failure, and significant alterations in RV haemodynamics were detectable prior to 9 of these episodes, 4±2 days before hospitalisation. Using the same device in a randomized controlled trial, Bourge et al. evaluated a remote monitoring strategy for 274 patients with heart failure.^18^ There was a non-significant reduction of 21% in heart failure related events in the remote monitoring group, with a significant 36% (p=0.03) reduction in heart failure related hospitalisation.

Intrathoracic impedance falls as pulmonary fluid accumulates during acute decompensation because electric current passage across the lung is enhanced.^19,20,21^ Thus, Yu et al. retrospectively evaluated an implantable system capable of intrathoracic impedance monitoring in 33 patients with advanced heart failure.^22^ During the study 10 patients were hospitalized for fluid overload a total of 25 times. Over an average of 18.3 (SD:10.1) days prior to admission, intrathoracic impedance decreased by an average of 12.3% (SD:5.3) (p<0.001). Using an automated detection algorithm, an impedance drop below a threshold level was 76.9% sensitive in detecting hospitalisation for fluid overload, with 1.5 false-positive (threshold crossing without hospitalisation) detections per patient-year of follow-up. A benefit of the intrathoracic impedance monitoring system over other potential implantable haemodynamic monitoring technologies is that ICDs with intrathoracic impedance monitoring capability are now widely available and used in clinical practice.^23,24,25^ During an episode of decompensated heart failure, changes in haemodynamics and fluid status are often detectable days to weeks prior to the onset of symptoms and hospital admission. Chaudhry et al. investigated the pattern of weight change prior to hospital admission, in a nested case-control study of 134 patients with hospital admissions with decompensated heart failure and 134 matched controls.^26^ They found that in cases versus controls, weight gain started ~30 days prior to decompensation, with a substantial increase in weight in the week prior to admission. From findings such as this has developed the concept of pre-emptive remote monitoring, where patient data is continuously collected without the presence of the patient, overcoming issues of compliance and anxiety which are disadvantages with patient-driven monitoring methods.^20,27,28^ If early identification of clinically relevant changes signals incipient decompensation, then early treatment may prevent further deterioration and hospital admission.^29,30^ Crucially previous studies of technology-based remote monitoring care pathways have focused on haemodynamic assessments to predict deterioration,^29^ but arrhythmias also have a significant impact on mortality, morbidity and hospitalisation rates.^31^ Sudden cardiac death, predominantly secondary to ventricular arrhythmias, is the commonest mode of cardiac death in all but the most advanced heart failure^3.^  Atrial fibrillation is present in around a third of patients hospitalised with heart failure and adversely impacts on prognosis. Furthermore, in 48 612 patients enrolled in the OPTIMIZE-HF study, arrhythmias contributed to 13.5% of heart failure hospitalisations.^32^ It is therefore likely that remote monitoring using data concerning arrhythmia burden as well as haemodynamics, as included in our study, will improve outcomes compared to the use of haemodynamic data alone.^28,31^

The PARTNERS HF study showed that monthly review of heart failure diagnostic data in devices could identify patients at higher risk of heart failure hospitalisation within the following month and suggests that algorithm-driven care of patients as planned in this protocol is likely to be effective.^33^ The PARTNERS HF study did not address the issue of whether interventions driven by risk identification could reduce patient hospitalisation. The RAPID RF study is intended to identify how remote monitoring can alter heart failure management.^30^ However, it uses only one monitoring technology and is simply a registry and does not employ specific remote monitoring pathways.

Currently about 50-100 patients per million population in the UK receive an implanted device (CRT-D or ICD) that is, at least in theory, capable of facilitating remote monitoring of heart failure (in addition to providing arrhythmia therapy) but cheaper monitoring-only devices could be indicated in as many as 5000 per million population if they are shown to provide effective care.^17,35,36^ The CHAMPION clinical trial study reported a 30% drop in the 6 month risk of HF hospitalisation using a pulmonary artery pressure sensor implanted transvenously in patients with moderately severe CHF.^37^ Thus clinical interventions early in the deterioration phase could prevent hospitalisation and mortality if mechanisms are in place to reliably detect the onset of such deterioration and effect therapy change. The care model and technologies used in this trial offer the potential for personalised health care through "remote" patient management in the community using secondary and tertiary care expertise and interventions as required, and challenge the usual patterns of healthcare delivery. We will test the generalisibility of the care pathways to different populations/communities and challenge usual patterns of healthcare delivery.

**Preparatory Feasibility Study:**

A single centre (University Hospital Southampton NHS Foundation Trust) prospective feasibility study was performed to aid design of remote monitoring pathways, give indicative costs and to gauge patient acceptance of intensive wireless remote monitoring versus a well developed community heart failure management service for patients with implanted devices. We recruited 80 patients with implanted devices (ICDs or CRT-Ds) to a non randomised comparison of remote monitoring versus usual care. Feasibility of patient recruitment and retention was tested with 86% of those eligible participating in the study. There were no drop outs in the remotely monitored patient population over the 6 month follow-up period. The sensor technologies and care pathways used were feasible in either changing drug dosages or organising necessary clinical assessment. Furthermore, at study completion, SF6D assessment demonstrated that quality of life was significantly improved in the remote monitoring group compared to the usual care patients, and cost utility modelling suggested a ratio within a range acceptable to NICE. As a feasibility study it was not powered to demonstrate any differences in outcome for a comprehensive remote monitoring care pathway. Its drawbacks were that it was non-randomised and this may have influenced baseline characteristics between the groups due to selection bias, with patients given remote monitoring-capable devices at implant as they were thought likely to accept the technology or to have clinical features likely to benefit from remote monitoring, such as atrial fibrillation or frequent episodes of ventricular tachycardia. Such a bias may have affected outcomes, though for factors we were able to measure baseline comparisons were similar (age, comorbidity) and in fact the remote monitoring group had greater severity of CHF. Follow-up was relatively short (6 months) and though remote monitoring was well accepted and associated with improved quality of life scores, it is not clear if these would persist long term. We concluded that remote monitoring of heart failure using implanted devices is well tolerated by patients even when used “aggressively” in a manner likely to facilitate pre-emptive management strategies for clinical deterioration. The clinical pathways used in this feasibility study were practical and well accepted by patients and healthcare professionals. The feasibility study set the scene for a major study powered to assess outcomes and which is the subject of this protocol.

**2. Study Importance**

This is the first study (to our knowledge) that attempts to evaluate new working patterns that will allow optimal management of HF patients in primary care using secondary/tertiary care clinical expertise. It will test changes in working practice that may lead to re-evaluation of other chronic disease management care structures that in turn will allow optimal use of healthcare resources to effectively manage costly and high volume disease states.

**3 Risk-Benefit Justification**

There is no increased risk to patients, as they will have access to currently available care pathways when required but will have the additional advantage of immediate but remote expert monitoring. However, the study will evaluate the acceptability of such monitoring to patients and their perception of this and any adverse impact on quality of life.

**4 Study Hypothesis**

We hypothesise that patient-independent remote monitoring using implanted device technology and deployed in clinical pathways designed for chronic management of heart failure in the home, will significantly reduce all-cause patient mortality and cardiovascular hospitalisation in the context of NHS care and will be more cost effective than usual care.

**5 Study Objectives**

- To determine the clinical and cost-effectiveness of remote disease management devices with pulmonary impedance measurements/other physiological assessments (using specialist nurses to monitor and initiate delivery of interventions) compared to usual care in patients with severe chronic heart failure.
- To evaluate patient and carer satisfaction of implanted monitoring devices for heart failure management.

**6 Inclusion and Exclusion Criteria**

***Inclusion Criteria:*** Devices will be either Implantable Cardioverter Defibrillators (ICDs) or Cardiac Resynchronisation Therapy-Defibrillators (CRT-Ds) or Cardiac Resynchronisation Therapy-Pacemakers (CRT-Ps) (as the required monitoring features are currently available only in those device types). Participants will all have received an ICD, CRT-P or CRT-D at least six months previously, for the treatment and monitoring of chronic heart failure. Patients receiving these devices will be doing so according to NICE guidance and/or local clinical discretion and not for the purposes of this study. In addition patients should meet all of the following criteria:1) Be on stable medical therapy for CHF for 6 weeks prior to recruitment; 2) Will have the ability to independently comprehend and complete Quality of Life Questionnaires; 3) Will have the ability to give informed consent; 4) Will be on optimal medical therapy according to the treating physician, working to NICE Guidelines; 5) Will have had their device programmed to give optimal therapy according to the treating physician; 6) Will have symptomatic heart failure (i.e. NYHA Class II to IV) documented at the time of study enrolment.; 7) Will be at least 30 days post any device change or lead replacement procedure; 8) Will be at least 3 months post any cardiac surgical procedure; 9) Will be at least 3 months post acute myocardial infarction.

***Exclusion Criteria:*** Participants will be excluded from the study if they meet any of the following criteria: 1) Unable to use the technology due to mental or physical limitations 2) Less than 18 years old 3) Pregnancy 4) On a heart transplant list 5) Life expectancy of < one year (non cardiovascular related) in the opinion of the treating physician 6) Current device related complications, e.g. wound infection or haematoma, lead fracture, 7) Device implanted less than 6 months previously. 8) Patients unable to understand written and spoken English.

**7 Staffing of the Study and Study Grant Costs**:

Staffing and costs have been calculated on basis of a preliminary feasibility study that has demonstrated feasibility of study design and practicality of service delivery and that indicates expectation of patient compliance/acceptance. Industry charges for use of the remote monitoring technology will be already made by the healthcare services as remote device management is now common clinical practice. If that is not the case it is anticipated that industry partners will waive charges for the duration of the study although the notional cost will be included in the study analysis.

**8 Recruitment and Randomisation**

Number and rate are known to be achievable on the basis of the feasibility study and analysis of patient populations/clinical practices at study sites. It is notable that during the pilot/feasibility study patient recruitment was at 86%. This is indicative of patient compliance with device therapy and is much more robust than would normally be expected for example for pharma studies.

In the feasibility study 454 patients were screened, 101 were eligible for inclusion, 80 consented, 16 refused and 5 patients were ring-fenced to avoid, due to complicated history or ongoing issues. Estimated numbers of prevalent cases in each centre at start of the study is 150 patients. Number of new cases estimated in each centre per annum is 150 patients. Recruitment will be closed when study complement is recruited.

Patients recruitment will be by a letter of invitation (Ethics approved) attendance at outpatient clinics and in patients (eg.HF episode - not device related complication).

Informed consent will be obtained by a study trained member of research staff and will include consent to inform GP + primary care team.

Randomisation:

This will be performed by an external agency (Tenelea, the Netherlands). Randomisation will be performed via the study specific electronic case report form (ECRF).. Patients will be randomised to either “usual care” or “remote management care pathway” via weekly downloads.

The randomisation schedule is centre specific. Stratification is by device type as they have different capabilities and case mix will differ i.e. by ICD, CRT-P and CRT-D. Randomisation will be in blocks of 4 and 6 randomly permuted to ensure concealed allocation.

**9 Study Details and Data Collection**

***Intervention: Remote Care Pathway:*** The remote-monitoring informed care pathway has been developed on the basis of the feasibility study and has been additionally informed by clinical experience and literature analysis by the co-applicants. The steering group has agreed the operational procedures that will govern the actions of the remote monitoring staff in response to the device-generated data. These have been formalized in procedural handbooks that have undergone pilot evaluation (see Appendix 2). The procedural handbook comprehensively deals with heart failure, arrhythmia and device management. In the active monitoring limb there will be weekly telemetering of data (downloads), as this was shown to be equally informative as daily in the feasibility work. The effector arm comprises protocol-driven changes in care co-ordinated by the site remote monitor (with consultant support as required) delivered through telephone support, primary care contact or direct patient contact in secondary/tertiary care.

***Usual Care Pathway:*** It is recognised that usual care pathways will differ between centres and that in many centres remote device follow up for technical checks on the devices is currently practiced. This usual device care will be unaffected as the study assesses the value and intensity of pre-emptive monitoring of disease state and **not** routine device follow up. As heart failure services are somewhat variable across the centres we will be comparing these non-standardised approaches to a standardised remote care package. This adds to the generalisability of the findings.

***Follow-up:*** minimum of 2 years.

***Outcomes measured*:**

The primary endpoint will be combined all-cause mortality (ACM) or unplanned cardiovascular (CV) hospitalisation whichever came first.

The Endpoint Review Committee will adjudicate on :

1. the appropriate classification of cause of death, using data from primary and secondary care records, coroner reports and the death certificates. Mortality data will be obtained from the NHS Medical Research Information Service (MRIS)
2. underlying cause of hospitalisation using a standardised proforma and reviewing circumstances of hospitalisation from primary care records and hospital notes.

Secondary outcomes will include ACM, unplanned CV hospitalisation, CV mortality, unplanned CV hospitalisation, and unplanned hospitalisation.

*Quality of Life Assessment:* All participants will complete the SF-12, EuroQol (EQ5D), and Kansas Heart Failure Questionnaire (KHFQ) at enrolment, 3 months, 6 months, 12 months and 24 months. These will be administered by the study remote monitors. The initial quality of life forms will be completed at the time of patient enrolment in the hospital. Subsequent assessments will be performed by the patients in their homes. The forms will be sent to them by post with the necessary means for return of the forms. Receipt of the forms and their return will be managed by the remote monitors and local study administration staff. *Cost-effectiveness:* including cost of interventions, cost of Cardiovascular related health care.

Process measures will be collected to compare the pattern of care between the two arms and across centres (as we have no standardisation of usual care and the protocol is a pragmatic guideline and interpretation may differ between centres).

Key abnormalities detected in RM relate to atrial/ventricular arrhythmias and HF events. Key interventions are medication patterns, including anti-coagulation for atrial fibrillation.

There will be assessment of implementation of recommended interventions in the RM group and particularly those performed in primary care.

This flow chart summarises the study:

*- Acute care*

In the event of symptomatic deterioration, patients in the remote management arm will be instructed to contact the heart failure study nurse according to local practice or remote monitor during office hours and to perform an immediate data download. The remote monitor will report in a prompt manner to the patient that the download has been received and will give the patient a management plan. This plan will be documented on the patient’s record. Execution of the management plan will be the responsibility of the primary care team and the remote monitor will communicate the plan during office hours on the day of the download to the primary care physician or his/her deputy with responsibility for that patient, so that clinical care changes can be implemented during the same working day. The primary care clinician will be asked to document receipt of that information and execution of a management change. In the event that the primary care clinician suggests an alternative plan then this will be communicated to the remote monitor.

*- Emergency care*

In the event of a clinical emergency or urgent out of hours/weekend deterioration the patient will access the emergency services in the usual way. However, the patient can be issued with a study identity card for presentation to clinical staff caring for the patient on hospital admission. This card will identify ways of downloading data to the remote monitor so that data relating to the emergency admission is captured in study records. It will not be used to guide emergency management.

**10 Data Analysis**

**Outcomes measured will be:**

**Primary endpoint:** Combined endpoint of all cause mortality or unplanned hospital admission for any cardiovascular event

**Secondary endpoints:** Quality of life using the EuroQol (EQ5D), SF12 (using physical and mental health component scale) and Kansas Heart failure questionnaire at 1 and 2 years.

Using survival analysis we will consider:

- ACM
- CV mortality
- Unplanned CV hospitalisation
- CV mortality or unplanned CV hospitalisation
- Cumulative number of unplanned CV hospitalisations using multinomial logistic regression (0,1,2,>2)

For harm see below under interim analyses: ACM and any unplanned hospitalisation.

**Costs**

Costs of both implanted devices and conventional heart failure monitoring will be measured. These will include the costs associated with dealing with data obtained. Also measured will be all costs associated with providing heart failure related care in both arms of the study.

***Sample size calculations:*** Our sample size calculations use estimates from previous studies: i) Intervention group event rate: The Companion^38^ and Care HF^39^ trials with a patient population comparable to our study control group, reported 48% for any death or cardiovascular disease hospitalisation at 2 years, and 39% at 29 months for cardiovascular death or unplanned hospitalisation. We anticipate slightly lower rates in the proposed due to their receiving better routine care, and so assume a 40% event rate in the control arm by 2 years. ii) Overall risk reduction: The meta-analysis of Clark et al^10^ with telemonitoring or structured telephone support, reported a 20% risk reduction. Hence we will assume that the intervention group has a rate of 32%. This size of effect (40% reduced to32%) is clinically relevant. Assuming 5% (2-sided) significance and 90% power, 546 events are needed to detect this size of difference in a log-rank survival comparison of the two trial groups. This requires 1394 patients in total (697 per group). We will inflate this total by approximately 20% to allow for attrition, giving a target recruitment of 1650. This sample size is sufficient to estimate the effect size in the component outcomes - ACM, cardiovascular disease unplanned hospitalisation - and is feasible.

***Statistical analysis:***

Cox proportional hazards regression will be used to analyse the primary outcome data according to the intention to treat. The stratifying variables will be entered as covariates and any clinically important baseline imbalance will also be accommodated in the model. Estimates will be reported as hazard ratios with 95% confidence intervals. Secondary clinical outcomes with time to event will be analysed in a similar way using a multifactorial survival analysis modelling method appropriate to the multicentre nature of the data (i.e. to allow for centre effects).

We will censor at time at study end for those alive and free of hospitalisation and at last date known to be free of hospitalisation for those who are lost to follow-up (e.g. leave the area). Withdrawals from RM will be recorded but such will be analysed at ITT.

Quality of life outcomes will be analysed at 1 and at 2 years.

From Euroqol : utility, VAS.

From SF12: Utility; Mental component score (MCS); Physical component score (PCS)

Kansas Heart failure numerical score.

Results will be analysed by multiple or logistic (if pre-specified categories) regression. Multiple imputation will be used to explore the impact of missing data in QoL data.

Cumulative number of unplanned CV hospitalisations will be analysed by multinomial logistic regression

A priori sub group analysis for the primary outcome will be conducted on the following variables:

Age

History of atrial fibrillation

Type of device (ICD, CRT-P,CRT –D)

Baseline functional status (NYHA grade, Kansas HF score).

***Interim analysis:***

We propose that the Independent Monitoring Committee (IMC) will review recruitment, data completeness and end points, and ensure no adverse effects from remote monitoring, at appropriate time points during the study.

For benefit we will use the primary outcome and Peto–Haybittle rule which uses a p value of p<0.001This is recommended by Pocock (Pocock SJ, Current controversies in data monitoring for clinical trials Clin Trials 2006;3:513e21) as it is conservative, would likely lead to a recommendation to stop only when there is a very convincing effect, thus preventing premature stopping of the trial, and it does not affect final analysis p values (final would be p=0.048) .

We plan one interim analysis based on primary events accumulated (at approximately 400 events).

For harm key outcomes will be ACM and any unplanned hospitalisation (e.g. if RM leads to over zealous use of diuretics leading to falls, acute renal failure) and we plan to undertake this interim analysis at the same time as benefit analysis.

**11 Cost Effectiveness**

The outcome measure used in the economic evaluation will be cost per quality adjusted life years (QALY). QALYs will be estimated using the EuroQol (EQ5D). Costs will be measured using an NHS perspective. As part of the study we will record the costs associated with providing remote monitoring, as well as any routine cardiac care received in each group. Resource use will be obtained from a variety of sources as appropriate, including hospital records and directly from participants. Where possible we will follow published guidelines for cost-effectiveness analysis, for example the NICE reference case and the BMJ guidelines for authors of economic evaluations.

**12 Training**

*Remote Monitoring Preparation*

Patients will be introduced to the remote monitor providing remote care and will be instructed in use of the care pathways.

Patients will receive training on use of remote monitoring through their implanted device by the study site remote monitor. They will receive their home monitoring transmission devices (specific model depending on implant device features). Devices will be interrogated using a monitor with automatic or manual download capabilities with either wireless or non wireless technology.

The remote monitor will communicate with the patients’ primary care services to inform them of entry to the study

**13 Project timetable:**

***STUDY TIME LINES:***

### Staff training & deployment

**Patient Follow-up**

**Data Analysis**

**Results Dissemination**

**Patient Recruitment**

**1-2**

**2-24**

**2-48**

**48-54**

**55-60**

**14 Data Storage**

For each enrolled patient, data will be stored in a specific study database. All aspects of local data recording and management will be performed by the remote monitor.

**15 Database**

An ECRF will be utilised to capture healthcare utilisation elements, QoL assessment and clinical events on a patient by patient basis allowing accurate and comprehensive assessment of care pathway. The Southampton Clinical Trials Unit is managing this. The data will be held by University Hospital Southampton NHS Foundation Trust and will conform to all Data Protection Requirements.

**16. Expected Value Of Results**

The NHS needs to develop different care mechanisms that enable effective use of technology to help better manage patients with chronic disease such as heart failure in the community, with safety, clinical efficacy, and overall patient benefit. We need to prove that the care model and technologies tested in this trial offer the potential for personalized health care through "remote" patient management in the community using secondary and tertiary care expertise and simple interventions as required. Our care model challenges usual patterns of healthcare delivery, bringing care and expertise closer to the patient. Dissemination of this technology in NHS care, in an optimal care pathway model, could greatly improve patient outcomes and the economics of CHF management, with planned therapy changes pre-empting symptomatic deterioration and the need for emergency hospitalisation, thus improving resource utilization. Improving health outcomes with reduced overall cost is needed more now than ever before, and this study may be an important step in that direction for the increasing number of people with heart failure who have an implanted device.

# 17. Safety Issues

Many of the study participants will have chronic illnesses and will be receiving treatment for heart failure and arrhythmias. Due to their illness, prolonged hospitalisation and re-admissions to hospital will be commonly occurring events during clinical care.

Similarly, study participants with (severe) underlying heart disease are at risk of dying during the course of the study as a result of the natural history of their disease/age.

In agreement with the Sponsor (Southampton) of this study, all Adverse Events including those normally classified within GCP as "serious" and result in hospitalisation or death will be documented in the ECRF and reviewed by an appropriately trained member of the research team. An SAE according to this protocol is considered to be any untoward medical occurance that results in death, requires inpatient unplanned hospitalisation or prolongation of existing hospitalisation. These Serious Adverse Events (SAE’s) will be assessed by a medically qualified team member.  Where an SAE is assessed as being related to study procedures, the reporting will be escalated to the Sponsor and the concerned hosting R&D Department. SAE’s not related to the study intervention are documented in the ECRF but will not be escalated to Sponsor or hosting R&D.

**18 Study Committees**

An Independent Monitoring Committee has been established under the chairmanship of Professor John Camm. Professor John Cleland sits on that committee together with an industry representative and a representative of the British Heart Foundation. The Co-PIs will attend meetings in open session but not in closed session during data review.

An independent End Point Adjudication Committee has been established under the chairmanship of Dr George Sutton.

A Data Monitoring Committee has been established to ensure patient safety. This committee is under the chairmanship of Professor Henry Dargie

*The Study Steering Group comprises:*

**Prof Martin Cowie**

CO PI - LONDON Imperial College, London

Contact E-mail [m.cowie@imperial.ac.uk](mailto:m.cowie@imperial.ac.uk)

**Prof John Morgan**

CO PI - University Hospital Southampton

Contact E-mail Jmm@hrclinc.org

**Dr Jaswinder Gill**

INVESTIGATOR - Guys and St Thomas

Contact E-mail [jaswinder.gill@gstt.nhs.uk](mailto:jaswinder.gill@gstt.nhs.uk)

**Dr Janet McComb**

INVESTIGATOR - Newcastle Freeman Hospital

Contact E-mail [Janet.McComb@nuth.nhs.uk](mailto:Janet.McComb@nuth.nhs.uk)

**Dr Andre Ng**

INVESTIGATOR - Leicester Glenfield Hospital

Contact E-mail [gan1@leicester.ac.uk](mailto:gan1@leicester.ac.uk)

**Prof Paul Roderick**

Professor of Public Health

Department of Medicine

Contact E-mail: pjr@soton.ac.uk

**Dr Alison Seed**

INVESTIGATOR - Blackpool Victoria Hospital

Contact E-mail [Dr.Seed@bfwhospitals.nhs.uk](mailto:Dr.Seed@bfwhospitals.nhs.uk)

**Prof James P Raftery**

Professor of health technology assessment

Contact E-mail: J.P.Raftery@soton.ac.uk

**Dr Simon Williams**

INVESTIGATOR - Manchester North West Heart Centre and Transplant Unit, South Manchester NHS Trust

Contact E-mail simon.williams@uhsm.nhs.uk

**Dr Klaus Witte**

INVESTIGATOR - Leeds University of Leeds

Contact E-mail [k.k.witte@leeds.ac.uk](mailto:k.k.witte@leeds.ac.uk)

**Dr Jay Wright**

INVESTIGATOR - Liverpool Heart and Chest Hospital

Contact E-mail [David.Wright@lhch.nhs.uk](mailto:David.Wright@lhch.nhs.uk)

**Mrs Sue Kitt**

Study Manager REM-HF- University Hospital Southampton

Contact E-Mail Sue.Kitt@uhs.nhs.uk

This committee will also act as Publications Committee under the chairmanship of the PIs. Intellectual property ownership will be governed by the BHF grant terms and conditions and after that, NHS-industry contractual negotiations, with data ownership residing with the steering committee.

# 19. Investigator responsibilities

Investigators must ensure that following Main REC approval, Site Specific Assessment (SSA) and local Trust R&D approval is obtained. All formal agreements between the coordinating centre and local Trusts will be signed prior to starting the study.

The study will be conducted in accordance with the Research Governance Framework for Health and Social Care (2005) and Good Clinical Practice.

# 20. Monitoring

The study will be monitored and audited in accordance with University Hospital Southampton NHS Foundation Trust (UHS) procedures. All trial related documents will be made available on request for monitoring and audit by UHS, the relevant REC or other regulatory bodies. The UHS trial manager will perform monitoring visits during the study or in conjunction with a close out visit. The purpose of this visit is to ensure compliance to the protocol and that ethical and regulatory guidelines are met

# 21. Premature termination

The trial may be terminated at a site by the coordinating centre after agreement with the Steering Committee if any of the following occurs:

The centre cannot comply with the requirements of the protocol.

The centre is unable to comply with the required data standards.

# 22. Indemnity

This is an NHS sponsored research study. For NHS sponsored research HSG (96) 48 reference no.2 refers. If there is negligent harm during the clinical trial when the NHS body owes a duty of care to the person harmed, NHS indemnity covers NHS staff, medical academic staff and honorary contracts, and those conducting the trial. NHS indemnity does not offer no fault compensation and is unable to agree in advance to pay compensation for non-negligent harm. Ex-gratis payments may be considered in the case of a claim.

**23. Study Personnel and Brief Job Descriptions**

**Remote monitoring staff**

Responsible for:

- Patient screening and recruitment
- Ordering of remote monitoring hardware
- Interpretation of downloads
- Communication with Clinical Staff
- Review of device troubleshooting
- Review of heart failure/arrhythmia data requiring therapeutic intervention according to study algorithms
- Review of any data in the light of adverse event
- All patient and clinical communications in relation to study management
- Contacting patients to document healthcare utilisation
- Collection and input of local study data into ECRF

**Study Administration Staff**

Responsible for:

- Liaising between site staff and Study Manager
- Collecting and collating healthcare utilisation data from local hospitals/GP surgeries.
- Provide administrative support to remote monitors such as sending out and collecting health questionnaires
- General study administration duties such as sending out REC approved GP letters.

**Study Trial manager**

Responsible for:

- Overall collection, input and management of data
- Reporting to regulatory authorities
- Collating of protocol deviation and serious adverse event data reported by remote monitors
- Collating data pertaining to patient deaths
- Overall management of study and resources
- Organising study training for relevant staff and updates of study progress to all study staff
- Liaising with trial statistician and data management staff
- Ensuring good communication between all sites and main study centre
- Reporting regularly to chief investigator
- Organisation of initial training programmes

**Senior Clinical Staff**

Responsible for:

- Overall supervision of study performance in their centre
- Review of troubleshooting data and heart failure/arrhythmia burden as requested by monitor
- Responsible as local investigator for their centre
- Oversight roles and communication with clinical staff

**Trial statistician**

Responsible for:

- Maintaining quality and completeness of all data collected in conjunction with trial co-ordinator and data manager
- Data checking, cleaning and statistical analysis.
- Preparing analyses and reports for endpoint committee.
- Contributing to writing of all reports and papers

**Lead Steering Group**

Responsible for oversight of study, with delegation of primary supervision of several aspects of the study to:

- Lecturer: Cost effectiveness analysis
- Public health consultant: Statistical design and liaison with Data Monitoring Committee
- Arrhythmia and Heart Failure Leads: supervision of relevant aspects of study performance

**19 References**

1. Capewell S, Allender S, Critchley J, et al. Modelling the UK burden of cardiovascular disease to 2020 A Research Report for the Cardio & Vascular Coalition and the British Heart Foundation. *British Heart Foundation*, September 2008.
2. Dickstein K, Cohen-Solal A, Filippatos G, et al. ESC Guidelines for the diagnosis and treatment of acute and chronic heart failure 2008 The Task Force for the Diagnosis and Treatment of Acute and Chronic Heart Failure 2008 of the European Society of Cardiology. Developed in collaboration with the Heart Failure Association of the ESC (HFA) and endorsed by the European Society of Intensive Care Medicine (ESICM). *Eur J Heart Fail* 2008; **29**: 2388-2442.
3. Mozaffarian D, Anker SD, Anand I, et al. Prediction of mode of death in heart failure: the Seattle Heart Failure Model. *Circulation* 2007; **116**: 392-8.
4. Hjalmarson A, Goldstein S, Fagerberg B, et al. Effect of metoprolol CR/XL in chronic heart failure. Metoprolol CR/XL Randomized Intervention Trial in Congestive Heart Failure (MERIT-HF). *Lancet* 1999; 353:2001–2007.
5. Hospital Episode Statistics 2008-9. Available at: <http://www.hesonline.nhs.uk/Ease/servlet/ContentServer?siteID=1937&categoryID=203> (last accessed 3 July 2010)
6. Davies, MK., Hobbs, FDR., Davis, RC. et al. Prevalence of left-ventricular systolic dysfunction and heart failure in the Echocardiographic Heart of England Screening Study: a population based study. *Lancet* 2001; 358: 439-44.
7. Fox M, Mealing S, Anderson R, et al. The clinical effectiveness and cost-effectiveness of cardiac resynchronisation (biventricular pacing) for heart failure: systematic review and economic model. *Health Technol Assess* 2007; **11:** iii-iv, ix-248.
8. 2000 – 2007 Hospital Episodes and Statistics; *Department of Health* 2001; The Stationary Office.
9. Al-Rousan M, Al-Ali AR, Eberlein A. Remote patient monitoring and information system. *Int J Electron Healthc* 2006; **2**: 231-49.
10. Blanchet KD. Remote patient monitoring. *Telemed J E Health* 2008; 14:127-30.
11. Boriani G, Diemberger I, Martignani C, et al. Telecardiology and remote monitoring of implanted electrical devices: the potential for fresh clinical care perspectives. *J Gen Intern Med* 2008; 23 Suppl 1:73-7.
12. Cotter G, Felker GM, Adams KF, et al. The pathophysiology of acute heart failure--Is it all about fluid accumulation. *American Heart Journal* 2008; **155**: 9-18.
13. Klersy C, De Silvestri A, Gabutti G, et al. A meta-analysis of remote monitoring of heart failure patients. *J Am Coll Cardiol* 2009; 54: 1683-1694.
14. Clark RA, Inglis SC, McAlister FA, et al Telemonitoring or structured telephone support programmes for patients with chronic heart failure: systematic review and meta-analysis. *British Medical Journal* 2007; **334**: 910-1.
15. Dar O, Riley J, Chapman C, et al. A randomized trial of home telemonitoring in a typical elderly heart failure population in North West London: results of the Home-HF study. *European J Heart Fail* 2009; **11**: 319-325.
16. Montara A, Pinna GD, Johnson P, et al HHF Investigators. *European J Heart Fail* 2009; 11: 227-8.
17. Adamson PB, Magalski A, Braunschweig F, et al. Ongoing right ventricular hemodynamics in heart failure: clinical value of measurements derived from an implantable monitoring system. *J Am Coll Cardiol* 2003; **41**: 565-571.
18. Bourge RC, Abraham WT, Adamson PB, et al. Randomized controlled trial of an implantable continuous hemodynamic monitor in patients with advanced heart failure: The COMPASS-HF Study. *J Am Coll Cardiol* 2008; 51: 1073-1079.
19. Wang L. Key lessons from cases worldwide. *Am J Cardiol* 2007; **21**: 34G-40G.
20. Ypenburg C, Bax JJ, van der Wall EE, Schalij MJ, van Erven L. Intrathoracic impedance monitoring to predict decompensated heart failure. *Am J Cardiol* 2007; **99**: 554-7.
21. OptiVol fluid status monitoring with an implantable cardiac device: a heart failure management system. *Expert Rev Med Devices* 2007; 4: 775-80.
22. Yu C-M, Wang L, Chau E, Chan RH-W, Kong S-L, Tang M-O, et al. Intrathoracic impedance monitoring in patients with heart failure: correlation with fluid status and feasibility of early warning preceding hospitalisation. *Circulation* 2005; **112**: 841-848.
23. Cowie MR, Md, Conraads V, Tavazzi L, Yu,CM. Rationale and design of a prospective trial to assess the sensitivity and positive predictive value of implantable intrathoracic impedance monitoring in the prediction of heart failure hospitalisations: The SENSE-HF Study. *Journal of Cardiac Failure* 2009; **15**: 394-400.
24. Braunschweig F, Ford I, Conraads V, Cowie MR, et al; DOT-HF steering committee and investigators.Can monitoring of intrathoracic impedance reduce morbidity and mortality in patients with chronic heart failure? Rationale and design of the Diagnostic Outcome Trial in Heart Failure (DOT-HF). *Eur J Heart Fail* 2008;10: 907-16.
25. Catanzariti D, Lunati M, Landolina M, et al. Monitoring intrathoracic impedance with an implantable defibrillator reduces hospitalisations in patients with heart failure *PACE* 2009; **32**: 363-70.
26. Chaudhry SI, Wang Y, Concato J, et al. patterns of weight change preceding hospitalisation for heart failure. *Circulation* 2007; 116: 1549-1554.
27. Theuns DA, Jordaens LS. Remote monitoring in implantable defibrillator therapy. *Neth Heart J* 2008; **16**: 53-6.
28. Ricci RP, Morichelli L, Santini M. Home monitoring remote control of pacemaker and implantable cardioverter defibrillator patients in clinical practice: impact on medical management and health-care resource utilization. *Europace* 2008;**10**:164-70.
29. Bratan T, Clarke M. Optimum design of remote patient monitoring systems. *Conf Proc IEEE Eng Med Biol Soc* 2006; **1**: 6465-8.
30. Small R. Integrating monitoring into the infrastructure and workflow of routine practice: OptiVol. *Rev Cardiovasc Med* 2006; **7** Suppl 1: S47-55.
31. Shotan A, Garty M, Blondhein DS, et al. Atrial fibrillation and long-term prognosis in patients hospitalized for heart failure: results from heart failure survey in Israel (HFSIS). *Eur Heart J*; **31**: 309-17.
32. Chen J, Wilkoff BL, Choucair W, et al. Design of the Pacemaker REmote Follow-up Evaluation and Review (PREFER) trial to assess the clinical value of the remote pacemaker interrogation in the management of pacemaker patients. *Trials* 2008; **3**: 9 -18.
33. Small RS. Integrating device-based monitoring into clinical practice: insights from a large heart failure clinic. *Am J Cardiol* 2007; **21**: 17G-22G.
34. Whellan DJ, Ousdigian KT, Al-Khatib SM, et al. Combined heart failure device diagnostics identify patients at higher risk of subsequent heart failure hospitalisations: results from PARTNERS HF (Program to Access and Review Trending Information and Evaluate Correlation to Symptoms in Patients With Heart Failure) study. *J Am Coll Cardiol* 2010; **27**:1803-10.
35. Ho C. Implantable hemodynamic monitoring (the Chronicle IHM system): remote telemonitoring for patients with heart failure. *Issues Emerg Health Technol* 2008; **111**: 1-4.
36. Pacemakers and Implantable Defibrillators: UK National Survey 2006. [www.devicesurvey.com](http://www.devicesurvey.com)
37. Abraham WT, Adamson P. CHAMPION Clinical Trial Study. Proceedings from *Heart Failure Congress* 2010.
38. Bristow MR, Saxon LA, Boehmer J, et al. Cardiac-resynchronization therapy with or without an implantable defibrillator in advanced chronic heart failure. *N Engl J Med* 2004; **350**: 2140-2150.
39. [Cleland JG](http://www.ncbi.nlm.nih.gov/pubmed?term=%22Cleland%20JG%22%5BAuthor%5D), [Daubert JC](http://www.ncbi.nlm.nih.gov/pubmed?term=%22Daubert%20JC%22%5BAuthor%5D), [Erdmann E](http://www.ncbi.nlm.nih.gov/pubmed?term=%22Erdmann%20E%22%5BAuthor%5D), [Freemantle N](http://www.ncbi.nlm.nih.gov/pubmed?term=%22Freemantle%20N%22%5BAuthor%5D), [Gras D](http://www.ncbi.nlm.nih.gov/pubmed?term=%22Gras%20D%22%5BAuthor%5D), [Kappenberger L](http://www.ncbi.nlm.nih.gov/pubmed?term=%22Kappenberger%20L%22%5BAuthor%5D), [Tavazzi L](http://www.ncbi.nlm.nih.gov/pubmed?term=%22Tavazzi%20L%22%5BAuthor%5D); [Cardiac Resynchronization-Heart Failure (CARE-HF) Study Investigators](http://www.ncbi.nlm.nih.gov/pubmed?term=%22Cardiac%20Resynchronization-Heart%20Failure%20(CARE-HF)%20Study%20Investigators%22%5BCorporate%20Author%5D). The effect of cardiac resynchronization on morbidity and mortality in heart failure. [*N Engl J Med*](javascript:AL_get(this,%20'jour',%20'N%20Engl%20J%20Med.');) 2005; **352**: 1539-49.

**Appendix I:**

**Scoping cost effectiveness assessment**

*Cost of existing services*

Each HF patient costs the NHS around £1.2k pa, with the bulk (65%) of this due to inpatient admissions, due to a mean admission per patient of 0.32.

For a cohort of 100 patients the total cost is £120k, with some £77k due to inpatient admissions

*Scope for cost savings due to new service*

Assuming the new service led to reduced admissions, then to achieve savings of £155 per person the inpatient admission rate would have to reduce by 20% and to achieve the higher £620 per patient it would have to reduce 80%.

**Table 1**

**Illustrative cost of new service**

| Assumptions |  |  |  |  |  |
| --- | --- | --- | --- | --- | --- |
| Nurse | wte | 0.25 | 0.5 | 0.75 | 1 |
| Cost (gross £60k) |  | 15k | 30k | 45k | 60k |
| Patients | 100 |  |  |  |  |
| no requiring action |  | 5% | 10% | 15% | 20% |
| cost of action £100 |  | 500 | 1000 | 1500 | 2000 |
| Total service cost |  | 15.5k | 31k | 46.5k | 62k |
|  |  |  |  |  |  |

*Cost per QALY of new service*

The above analysis indicates that the new service may need to have large reductions on the need for inpatient admissions in order to be cost saving. However, the service could still be cost-effective even if not cost-saving. The descriptive statistics in appendix one indicate that he mean EQ5D score of those providing the cost data was 0.55. If we assume that the monitoring service and timely intervention could improve quality of life by either 0.02 or 0.05 we can estimate the likely cost per QALY of the new service (table 3). This is based on the QALY gain lasting for 1 year. Table 3 examines a number of different scenarios. These are: nurse service requires either 25% or 100% WTE, 0%, 20%, or 80% of inpatient admissions are saved and the nurse service refers between 5% and 200% of patients per year. It can be seen from table 3 that for a 0.02 QALY gain and a 25% WTE nurse service the cost per QALY appears to lie between £7,750 and £17,500 if no inpatient admission savings occur. These values are greatly reduced if 20% savings are assumed and under 80% savings the service would be cost saving. For the 0.02 QALY gain group all estimates lie between £40,000 and cost saving. If we assume a 0.05 QALY gain then all estimates like below £16,000 per QALY.

**Table 2**

**Mean NHS cost per HF patient and reductions in inpatient admission rate to achieve savings equal to the cost of the new service**

| Resource use |  |  |  |
| --- | --- | --- | --- |
|  |  |  |  |
|  | Mean |  |  |
| GP appointments | 2.74 |  |  |
| PN Visits | 0.76 |  |  |
| Outpatient visits | 1.14 |  |  |
| Inpatient | 0.32 | 0.062672 | 0.255668 |
| % change |  | -80.415 | -0.20104 |
|  |  |  |  |
|  |  |  |  |
| Costs |  |  |  |
|  |  |  |  |
|  | Mean |  |  |
| Beta Blockers | £19 |  |  |
| ACE | £59 |  |  |
| Statins | £97 |  |  |
| Nitrates | £15 |  |  |
| CCB | £34 |  |  |
| Loop diuretics | £13 |  |  |
| Other diuretics | £9 |  |  |
| Other CHD relevant medicines | £43 |  |  |
| Total Medicines | £289 |  |  |
| GP appointments | £52 |  |  |
| PN Visits | £7 |  |  |
| Outpatient visits | £89 |  |  |
| Inpatient visits | £771 | £151 | £616 |
| Total costs | £1,208 |  |  |

**Table 3 – Estimated cost per QALY per person year. Values are for varying assumptions regarding QALY gain and service specification**

| **Assuming 0.02 QALY gain per year** | | | | |
| --- | --- | --- | --- | --- |
|  | % Referred | Inpatient admissions saved | | |
|  |  | 0% | 20% | 80% |
| 25% WTE Nurse | 0.05 | 7,750 | 37 | Cost Saving |
|  | 0.2 | 8,500 | 787 | Cost Saving |
|  | 2 | 17,500 | 9,787 | Cost Saving |
| 100% WTE | 0.05 | 30,250 | 22,537 | Cost Saving |
|  | 0.2 | 31,000 | 23,287 | 60 |
|  | 2 | 40,000 | 32,287 | 3,660 |
| **Assuming 0.05 QALY gain per year** | | | | |
|  |  | Inpatient admissions saved | | |
|  |  | 0% | 20% | 80% |
| 25% WTE Nurse | 0.05 | 3,100 | 15 | Cost Saving |
|  | 0.2 | 3,400 | 315 | Cost Saving |
|  | 2 | 7,000 | 3,915 | Cost Saving |
| 100% WTE | 0.05 | 12,100 | 9,015 | Cost Saving |
|  | 0.2 | 12,400 | 9,315 | 60 |
|  | 2 | 16,000 | 12,915 | 3,660 |

**Annual costs of heart failure (from Leicester study)**

Costs are taken from a trial of a nurse led disease management programme^1;2^. Costs are for the year previous to the introduction of the intervention being evaluated in the above trial and are for the year 2003/4. The data did not specify if any patient had ICDs but these would probably not be common (if present) so the costs are for a non-ICD population. It is unclear how representative these costs would be of the individuals included in any pilot study.

Descriptive statistics

| N=391 |  |
| --- | --- |
| Age | 72 |
| Sex (percentage male) | 61% |
| EQ5D | 0.55 |

Resource use

|  |  | 95% CI | |
| --- | --- | --- | --- |
|  | Mean | lower | upper |
| GP appointments | 2.74 | 2.40 | 3.08 |
| PN Visits | 0.76 | 0.53 | 0.98 |
| Outpatient visits | 1.14 | 0.95 | 1.33 |
| Inpatient | 0.32 | 0.25 | 0.39 |

**Costs**

|  |  | 95% CI | |
| --- | --- | --- | --- |
|  | Mean | lower | upper |
| Beta Blockers | £19 | £14 | £23 |
| ACE | £59 | £52 | £66 |
| Statins | £97 | £83 | £112 |
| Nitrates | £15 | £11 | £19 |
| CCB | £34 | £28 | £40 |
| Loop diuretics | £13 | £11 | £15 |
| Other diuretics | £9 | £7 | £11 |
| Other CHD relevant medicines | £43 | £33 | £54 |
| Total Medicines | £289 |  |  |
| GP appointments | £52 | £46 | £59 |
| PN Visits | £7 | £5 | £9 |
| Outpatient visits | £89 | £74 | £104 |
| Inpatient visits | £771 | £508 | £1,035 |
| Total costs | £1,208 |  |  |

**Appendix II.**

**REM-HF**

**Protocol Handbook**

**For Remote Monitoring Staff**

**Contents:**

1. **Introduction**
2. **Reporting Structure**
3. **Heart Failure management**
4. **Atrial Arrhythmia management**
5. **Ventricular Arrhythmia management**
6. **Device Troubleshooting**
7. **Ischaemia**

**1 Introduction**

This handbook gives an overall guide to the interpretation of monitored data form implanted devices in patents with heart failure who are enrolled in the REM-HF study.

It supplements the Monitors’ Training Programme. This handbook will be used as an aide memoire during the two-week training course that will be delivered to all monitoring staff at study commencement and will therefore be supplemented by training material provided during the induction/training courses.

Whilst it endeavours to be comprehensive and give direction on interpretation of monitored data and appropriate clinical responses, it is not intended to entirely replace common sense clinical approaches.

The monitor should be prepared at all stages to consider discussion of the care issues with the supervising senior clinical staff.

**2) Reporting Structure**

The Remote Monitor will access the monitoring websites and analyse downloads.

They will interpret the data or when necessary ask for assistance in data interpretation. The data comprises information about device functionality and about patient disease state.

Disease state is assessed by heart failure management status, atrial arrhythmia management, and ventricular arrhythmia management.

The different manufacturers’ devices have similar disease management potentials. The job of the monitor is to interpret the monitored data and communicate meaningful information to primary, secondary and tertiary care that will assist with patient management.

The case report forms for the study have the communication strategy for each patient identified.

Thus, at study enrollment the monitor will identify those colleagues with whom they must communicate for each patient. The services available to support patients differ between centres and therefore the clinicians who will action primary care/community-based therapy changes will differ between centres and within centres. However, the management change plan will be consistent across the study.

The monitor will interpret the download data, accessing support/help from the centres’ cardiac technicians/consultant cardiologists as wanted. The outcome of this process will sometimes be a management change. This may be:

- Drug change: actioned through nominated primary care contacts (GP/community heart failure nurse).
- Primary care test: (e.g. electrolyte estimation) actioned through nominated primary care contacts (GP/community heart failure nurse)
- Specialist test: actioned at the local centre
- Programming change or specialist intervention: actioned at the local centre


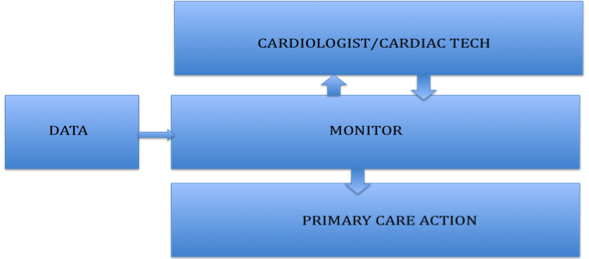


**3) Heart Failure Management**

Heart failure is a chronic condition with the risk of episodic deterioration (‘decompensation’). Aims of therapy include stabilisation of the syndrome, improvement in the prognosis and quality of life, and the avoidance of hospitalisation. Early detection of deterioration is a key component of heart failure management programmes, with the goal of adjusting therapy to re-stabilise the syndrome rapidly and to avoid the need for emergency hospitalisation.

Professional guidelines suggest that all patients recently hospitalised with heart failure (and other ‘high risk’ patients) should enter a heart failure management programme. Only around 20% of such patients in England do so at the present time but the centres involved in REM-HF have such programmes. Many of the key components of such a programme relate to monitoring (Table 1).

| ***Table 1. Recommended components of heart failure management programmes in the European Society of Cardiology Guidelines 2008 ^(2)^*** |
| --- |
| - **Multidisciplinary approach frequently led by HF nurses in collaboration with physicians and other related services** |
| - **First contact during hospitalization, early follow-up after discharge through clinic and home-based visits, telephone support and remote monitoring** |
| - **Target high risk, symptomatic patients** |
| - **Increased access to healthcare (telephone, remote monitoring, and follow-up)** |
| - **Facilitate access during episodes of decompensation** |
| - **Optimized medical management** |
| - **Access to advanced treatment options** |
| - **Adequate patient education with special emphasis on adherence and self-care management** |
| - **Patient involvement in symptom monitoring and flexible diuretic use** |
| - **Psychosocial support to patients and family and/or caregiver** |

There is much that the patient and their family can do to monitor how well the heart failure syndrome is controlled. Professional monitoring is likely to be supplemental to this self-monitoring. Self monitoring should facilitate self-management, where a patient adjusts their therapy depending on the control of the heart failure syndrome. Typically this would involve a patient adjusting the dose of diuretic depending on changes in their weight. However, patients experience a variety of symptoms, which complicates their ability to recognise the importance of symptoms and to identify their cause as heart failure-related. Older age, depression and cognitive dysfunction may decrease self-care ability. Some patients are able to self-monitor well, but do not then make the recommended changes in diuretic dosage, despite past experience and educational attempts by the healthcare team.

The National Institute for Clinical Excellence has made recommendations for monitoring of heart failure. (Table 2)

| ***Table 2. Recommendations from NICE for the monitoring of patients with CHF. ^(2)^*** |
| --- |
| **All patients with chronic heart failure require monitoring. This monitoring should include*:** |
| - **A clinical assessment of functional capacity, fluid status, cardiac rhythm (minimum of examining the pulse), cognitive status and nutritional status** |
| - **A review of medication, including need for changes and possible side-effects** |
| - **Serum urea, electrolytes and creatinine** |
| **More detailed monitoring will be required if the patient has significant co-morbidity, or has deteriorated since the previous review.** |
| **The frequency of the monitoring should depend on the clinical status and stability of the patient. The monitoring interval should be short (days to 2 weeks) if the clinical condition or medication has changed, but is required at least six monthly for stable patients with proven heart failure.** |
| **Patients who wish to be involved in their monitoring of their condition should be provided with sufficient education and support from their healthcare professional to do this, with clear guidelines as to what to do in the event of deterioration.** |

*This is a minimum. Patients with co-morbidities or co-prescribed medications will require further monitoring. Monitoring serum potassium is particularly important if a patient is taking digoxin or spironolactone.

In REM-HF the ‘control’ group are managed according to the local standard of care (likely to be at least as good as those recommended by the ESC (Table 1) and NICE (Table 2), but in the patients randomised to remote monitoring, weekly remote interrogation of the CRT-D or ICD device will provide **additional** information. This may allow earlier identification of preclinical signs of worsening heart failure and earlier identification of arrhythmic problems (see subsequent sections).

For those randomised to remote monitoring through the implanted device, additional information will be available on:

1. Mean daily physical activity via movement detectors
2. heart rate variability
3. intra-thoracic impedance

These data (including the trends over time and pattern recognition of the ‘usual’ sequence of events for an individual patient) will be reviewed by the remote monitor, and a decision made as to when to contact the patient for further information (such as request for information on symptom change or body weight). In the light of this further information, a decision will be made as to what is the most appropriate course of action.

This may be one or more of several options:

1. to do nothing other than review the situation remotely within a short space of time,
2. reinforce lifestyle advice (e.g. diet, salt intake, exercise),
3. make changes to medication (such as increase in diuretic dosage)
4. encourage compliance with medication,
5. trigger a clinical review by the primary or secondary care team.

This decision may require discussion with the clinical team normally reviewing the patient, such as the cardiologist, heart failure nurse specialist and/or primary care physician.

In general, decompensation of heart failure would be recognised by one or more of the following on the remotely collected data: decreased heart rate variability, decreasing intra-thoracic impedance, and decreasing mean daily physical activity. Not all of these changes will necessarily occur together, and the time course of such changes may vary from one patient to another and from one episode of decompensation to another.

**Corroboration** of the evidence for decompensation should be sought by enquiry regarding symptoms (by telephoning the patient) and trend in body weight if the patient has been weighing themselves regularly. As time goes on the pattern for an individual patient will become clearer e.g. intra-thoracic impedance may be very useful in some patients, but in others it may falsely suggest decompensation and should be corroborated by other data.

Over-reliance on the remotely collected data should be avoided: if a patient reports greatly increased symptoms (or weight) without any change in the monitored parameters, clinical assessment will be required to ascertain the reason for deterioration.

Deterioration in the heart failure syndrome may be associated with change in cardiac rhythm or frequency of AF/AFl or ventricular arrhythmia. The remotely monitored data should be reviewed as a **whole**, rather than piecemeal. Please see sections below.

**4) Atrial Arrhythmia Management**

**Overview**

Management of atrial fibrillation and atrial flutter (AF/AFl) only are discussed here because both these arrhythmias are common occurrences in heart failure patients. It is of course possible that these patients may have any other type of supraventricular tachycardia (SVT) and if reentry SVTs occur then formal cardiology review will be required, although such arrhythmias will be much less common than AF/AFl. An exception may be sinus tachycardia with first degree heart block and monitors will be expected to consider this as a possible cause of tachycardia with 1:1 A: V conduction and ensure discussion with technical staff for appropriate device programming to ensure discrimination from ventricular tachycardia. This issue will be specifically addressed during the training weeks.

Returning to AF/AFl, there are three broad areas of concern.

1. Has device programming taken into account their occurrence and the

consequences for detection/discrimination from ventricular arrhythmias?

1. Does the arrhythmia compromise patient well-being in terms of perception of palpitation or worsening heart failure and therefore do treatments need to be instituted to deal with rate/rhythm control?
2. Has the thromboembolic risk been addressed?

It should also be noted that the monitoring afforded by the implanted devices used in the study and the monitoring regime may unmask a burden of AF/AFl that would otherwise go undetected during conventional clinical care and therefore the above issues may be addressed in patients who would not ordinarily come to clinical attention. For the purposes of the study we will be assuming that the evidence base that informs management decisions for AF/AFl also informs appropriate management of these patients. The steering committee considers this to be a reasonable assumption.


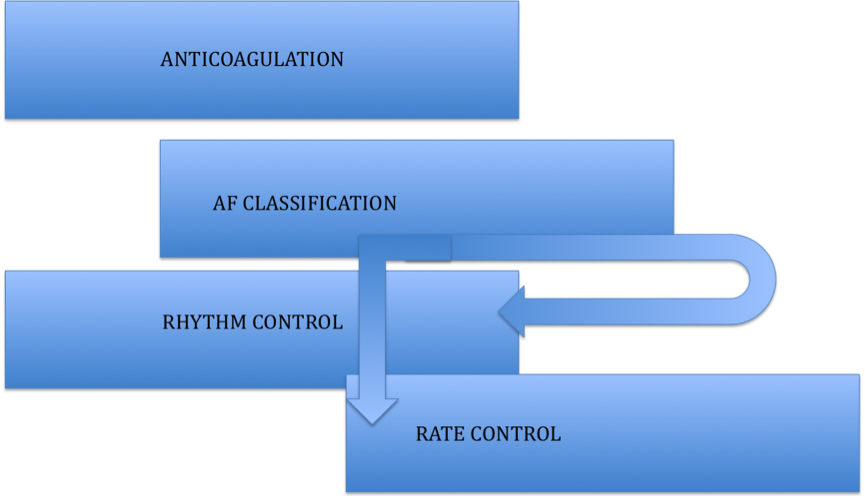
**AF**

***Background Considerations:***

*Anticoagulation Therapy and Thromboembolic risk*

This is an issue independent of other treatment strategies. There is clear guidance about its use depending on CHADS score:

Familiarise yourself with the CHADS scoring system for initiation of anticoagulation therapy in patients with atrial fibrillation. The above papers are attached.

The Steering Committee recommends warfarinisation with an INR of 2-3 for patients with a CHADS score > 1.

***Symptomatic AF***

When patients are complaining of palpitation then clinical assessment will be required in many. They are likely to be only a minority of the total number of patients with AF. In the first instance discuss the details of symptoms over the telephone, consider all the issues to do with rate/rhythm control (see below) and anticoagulation and then after those have been addressed and actioned offer a consultation if symptoms continue*.*

***Rhythm Management Options***

You will know that atrial fibrillation has been classified as:

- paroxysmal
- persistent
- permanent,

Given the above, on diagnosis of AF on monitoring, ask:

- Whether the patient is taking appropriate antithrombotic therapy?
- Is the patient symptomatic?
- Is AF causing worsening heart failure?
- Is the atrial arrhythmia paroxysmal, persistent or permanent and if paroxysmal, is it
- first episode
- recurrence

***Strategy*:**

*Step 1:* Initiate appropriate anticoagulation regime.

*Step 2:* Consider device programming.

a) Therapy for AF may affect device parameters. Consider detection and discrimination parameters and discuss with technician staff. In particular decided whether the rate is sufficiently fast to:

- Satisfy the VT/VF detection rate and lead to inappropriate shocks?
- Inhibit biventricular pacing?

Again answer to these questions may require discussion with technical staff and cardiology consultant. If in doubt consult them.

1. Therapy for AF may affect patient surveillance. If this is newly diagnosed and the patient is asymptomatic then increase frequency of downloads to twice weekly (and discuss with supervising cardiologist) for 4 weeks until the clinical situation is clearer. As a simple rule, if this is newly diagnosed atrial fibrillation and a remote treatment change is instituted, then increase the frequency of follow up to weekly for 4 weeks post management change.

*Step 3:* Consider treatment plan which may be:

- Rate control scenario
- Rhythm control scenario

A decision needs to be made as to what is the long-term rhythm control aim.

The decision could be determined by a number of clinical features and should be discussed with the supervising consultant who can make an informed decision about the overall direction of treatment strategy. This will not need face-to-face patient encounters although implementation of the chosen clinical strategy may do so. It will be influenced strongly by the atrial fibrillation classification. Thus, your task to is to “classify” the atrial fibrillation and then consider with the supervising physician a treatment plan.

Rhythm Control: Paroxysmal Self Terminating Atrial Fibrillation

In this instance rhythm control strategy will be adopted. If amiodarone is commenced consider the possible effect of this drug on defibrillation efficacy (see below). There is relatively less experience of the use of dronedarone in this scenario but if it is suggested by the responsible physician ensure that surveillance is maintained according to developing standards. Selection of any other antiarrhythmic drugs should follow careful clinical discussion.

Rarely an atrial fibrillation ablation strategy may be considered. Specifically raise this issue with the responsible physician. Again ask for a primary care assessment to assess electrolytes and renal function. Consider the burden of AF and how much it is limiting application of CRT.

Rhythm and Rate Control: Recurrent Persistent Atrial Fibrillation

In this instance a rate control strategy may be appropriate but if the duration and recurrence of atrial fibrillation is unclear then a decision may be taken to adopt a rhythm control strategy in the first instance.

*In the rhythm control scenario:* Consider with the supervising physician the use of appropriate drugs/cardioversion. Cardioversion may be considered using the device implanted to deliver the shock therapy. If amiodarone is commenced consider the possible effect of this drug on defibrillation efficacy (see below). There is relatively less experience of the use of dronedarone in this scenario, but if it is suggested by the responsible physician ensure that surveillance is maintained according to developing standards.

*In the rate control scenario:* Beta blockade will be the treatment of first choice. This could be commenced remotely in the absence of contraindications to beta blocker therapy – (check for these). Also assess whether there has been any other change in patient management recently and ask for a primary care assessment of electrolytes and renal function.

If already on adequate dose of beta blocker (and not on digoxin), consider digoxin, 500mcg bd for one day, then dose depending on age, weight and renal function. Ask for a primary care assessment to assess electrolytes and renal function.

Verapamil is a third line treatment.

In some circumstances AV node ablation may be the most reliable way of ensuring CRT therapy application and this should be specifically considered and discussed.

Permanent Atrial Fibrillation

A rate control strategy will be adopted. Assess any change in patient management recently and ask for a primary care assessment of electrolytes and renal function. Beta blockade will be the treatment of first choice. This can be commenced remotely though primary care in the absence of contraindications to beta blocker therapy – check for these. If already on adequate dose of beta blocker (and not on digoxin), consider digoxin, 500mcg bd for one day, then dose depending on age, weight and renal function. Verapamil is the third line treatment.

In some circumstances AV node ablation may be the most reliable way of ensuring CRT therapy application and this should be specifically considered and discussed.

Amiodarone is unlikely to be indicated.

***Specific Scenarios***

AF with rapid rate response (average rate > approximately 110 bpmin)

If AF with a rapid ventricular response rate is seen then rate control is relatively urgent, to avoid or reduce the possibility of inappropriate shocks.

Hospital admission may be required to do this, particularly if the patient presents with an inappropriate shock.

Alternatively, an urgent clinical assessment may be appropriate.

Beta blockade will be the treatment of first choice. This could be commenced remotely in the absence of contraindications to B blocker therapy – check for these. Also assess whether there has been any other change in patient management recently and ask for a primary care assessment of electrolytes and renal function.

If already on adequate dose of beta blocker (and not on digoxin), consider digoxin, 500mcg bd for one day, then dose depending on age, weight and renal function. Ask for a primary care assessment of electrolytes and renal function. Verapamil is a third line treatment.

If persistent but not permanent, and if not responsive to rate control, consider cardioversion indication

Consider also

- Has the patient had an exacerbation of heart failure?
- Has the patient got a chest infection?
- Has the patient got thyrotoxicosis? (Particularly if taking Amiodarone)

Amiodarone commencement

Amiodarone may interference with digoxin (digoxin dose may need to be reduced) and i*nterference with warfarin (*Warfarin dose may need to be reduced).

INR clinic appointments may need to be altered/increased in frequency. Also, if the patient has experienced VT then Amiodarone may impact on rate of VT: may be slowed sufficiently so that it fails to meet the detection rate.

It may also impact on defibrillation efficacy due to an increase in defibrillation threshold. This may need formal reassessment - discuss with the responsible physician. Also remember the need for regular surveillance, with six monthly TFTs, LFTs, which should be requested from primary care.

**AFl**

This is a particularly troublesome and not uncommon arrhythmia in CRT patients.

It may not trigger mode switch, and so may persist at relatively high rates, causing worsening heart failure.

Consider:

- Anticoagulation
- Rate control
- Cardioversion
- Cavo-tricuspid isthmus ablation.

In general cavo-tricuspid isthmus ablation will be the treatment of choice for common atrial flutter. For non-isthmus dependent flutter it is likely that ablation will remain the treatment of choice but the needs to be discussed with the responsible physician. Bring all episodes of atrial flutter to the attention of both the physiologist and responsible consultant and initiate a discussion around drug and ablation control.

**5) Ventricular Arrhythmias**

Summary chart:

**
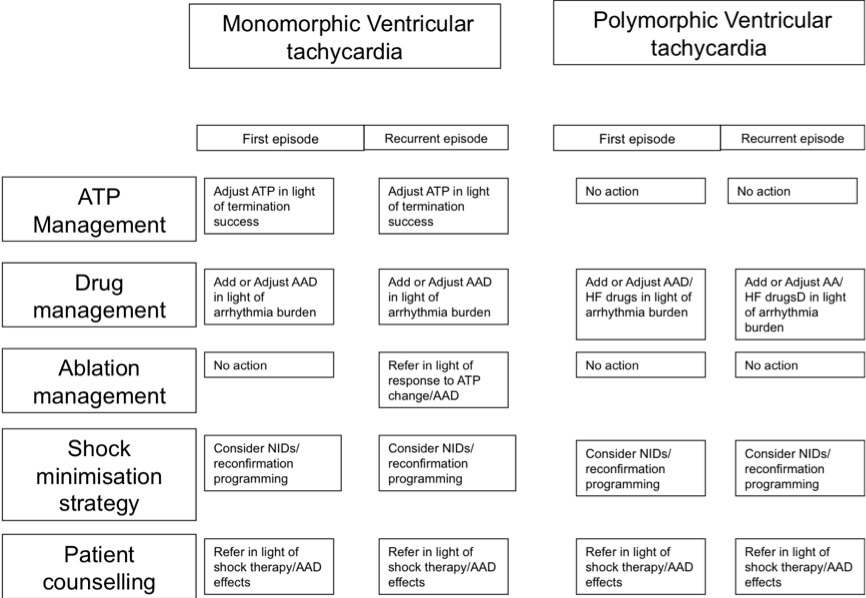
**

This chart summarises the approach to ventricular arrhythmias.

Your key role in management of ventricular arrhythmias is to identify their occurrence and change in burden. Both may indicate the need for a change in heart failure therapy, antiarrhythmia drug therapy, device programming (both for detection and therapy) and sometimes a further intervention such as ablation or device revision.

In the main you should discuss all these issues both with the technologist staff and the responsible physician. You should not initiate any therapeutic change without discussion with the responsible cardiologist.

However, you should be sufficiently acquainted with these issues to understand when it is appropriate to initiate a discussion about device therapy.

Do not forget to ensure that there are no changes that have taken place in patient medication or status that may have impacted on susceptibility to arrhythmia. Thus ask for primary care review of renal/electrolyte status and consider other co-morbidities or drug changes. Specifically ask patients questions to evaluate this. We are particularly keen to understand why change is occurring in arrhythmia stability status.

The two main arrhythmias to consider are management of monomorphic and polymorphic ventricular tachycardia as listed above.

Monomorphic ventricular tachycardia, even at fast rates, is often amenable to antitachycardia pacing. The precise programming of this can vary greatly and firm guidelines are difficult due to he breadth of the evidence base and its variability. It is necessary to ensure that all possible is done to avoid delivery of shock therapy when ATP my have been successful and this is the principle that will be adopted by technology staff and physicians. The concept of shock reduction will be specifically addressed during the training period and may be updated over the course of the study.

Antiarrhythmic drugs are useful adjuncts. In patients with a high burden of arrhythmia or with slow VT that is resistant to pace-termination and/or very frequent then catheter ablation may be an excellent therapy.

Polymorphic ventricular tachycardia is likely to be mush less amenable to ablation intervention but care should be taken to evaluate all the other issues mentioned above and in particular change in ischemia status or renal/electrolyte status.

Be aware that arrhythmia burden change will signal deterioration in heart failure status and all the heart failure issues in section 1 must be reviewed.

**Arrhythmia Storm**

When this occurs often the reasons/triggers are unknown. It will result in patient admission to hospital. Retrospective evaluation of data will be appropriate to see if issues were missed but management of the episode itself will be as an in-patient.

**Ventricular Fibrillation**

Patients may experience asymptomatic or nocturnal shock therapies for VF, which has no other impact on their well being. Having ascertained the accuracy of the arrhythmia diagnosis (i.e. appropriate shock therapy by analysis of stored electrograms) and efficacy of therapy no further action is required other than to look for precipitating factors (drug changes/co-morbidity change/renal-electrolyte status). Initiate these assessments in primary care. Also initiate weekly monitoring for 4 weeks to consider potential for underlying heart failure/arrhythmia burden change as a precipitant.

**Ventricular Ectopics**

In general these should be ignored unless causing unacceptable symptoms or reducing the efficacy of BiV pacing.

**6) Device Management**

It is intended that issues to do with device management will be handled by technology staff and the responsible clinicians. However you should familiarize yourself with the troubleshooting issues to allow high-level troubleshooting and to understand when you need to ask for advice.

The headline troubleshooting issues are summarized in the table below and these will be supplemented during the training programme.

| **Problem**  **TO BE IDENTIFIED BY MONITOR** | **Consider**  **DISCUSSION WITH CARDIAC TECH OR CARDIOLOGIST** | **Action**  **WITH CARDIOLOGIST AND CARDIAC TECH** |
| --- | --- | --- |
| **Appropriate shocks** | - Optimise ATP to successfully terminate arrhythmia | - Assess patient for reversible causes e.g. 12 lead ECG and U&E. - Optimise ATP regimes. - Consider anti-arrhythmic drugs - Advise about driving restrictions. - Address quality of life. - Dr assessment |
| **Inappropriate shocks** | - Rate - Rhythm - Discriminators - Noise | - Optimise programming e.g. discriminators, increase detection rate for VF/VT. - Treat misdiagnosed SVT e.g. drugs or ablation. - Check for over-sensing , impedance, lead fracture. - Send for CXR - Dr assessment. - ? turn off therapies |
| **Increased Threshold** | - Lead displacement - Lead fracture - Fluid accumulation | - Increase outputs - Send for CXR - Dr assess CXR - Lead replacement/ reposition. |
| **Increased Impedance**  **Decreased Impedance** | - Lead fracture - Insulation break | - Arm movement checks for inappropriate sensing - Reprogram sensitivity and mode - Check outputs - CXR - Dr assessment - Lead reposition/ replacement |
| **Over/under-sensing** | - Lead issue (fracture/ insulation break/displacement) | - Measure P and R waves - Check lead impedance (see above) - Alter sensitivity – only slightly in V lead - Dr assessment - CXR - DFT - Lead reposition replacement |

*Alerts*

“Alerts” will also be specifically addressed. The monitored device data can be flagged according to importance and will appear on the websites under “Alert” communications. The various manufacturers have, for the moment, to handle the issue of notification and data presentation in different ways. Therefore, during the training programme we will address Alert programming in such a way as to allow monitors to be consistent in their approach to their use whilst allowing flexibility.

The implanted devices are equipped with “Alert” facilities capable of recognising deterioration in either patient clinical status of change in device system functionality that may threaten system integrity/effectiveness. All Alerts will be programmed on but may be de-featured according to clinical judgement of the consultant cardiologist with clinical responsibility for the patient. This will not be a protocol violation but will be assessed in the study analysis.

In the event of an Alert being triggered then:

For non-automatic transmission the patient will contact the study nurse during office hours and perform a download.

The study nurse will inform the responsible senior clinician of all alert transmissions who will act on alerts and determine appropriate clinical action.

**7) Ischaemia**

At this stage, the role of devices in evaluating ischaemia burden is unclear. We may choose to adopt clinical practise during the course of the study as information and data become available. This will be discussed during training and updates.

It will be the role of the supervising consultant cardiologist to action review and therapy for ischaemia.

**Bibliography**

**HF**

National Institute of Clinical Excellence (2003). National clinical guideline for management of chronic heart failure in primary and secondary care. Available at: <http://www.nice.org.uk/nicemedia/pdf/Full_HF_Guideline.pdf>. (accessed 12 June 2010)

Dickstein K, Cohen-Solal A, Filippatos G et al (2008). European Society of Cardiology Guidelines for the diagnosis and treatment of chronic heart failure. European Heart Journal. 29, 2388 – 2442

Nicol ED; Fittall B; Roughton M; Cleland JGF; Dargie H; Cowie MR. (2008). NHS heart failure survey: a survey of acute heart failure admissions in England, Wales and Northern Ireland. Heart. 94,172-177

Rogers A, Addington-Hall J, Abery A et al (2000) Knowledge and communication difficulties for patients with chronic heart failure. BMJ. 321, 605 – 607.

Horowtiz CR, rein SB, Leventhal H (2004). A story of maladies, misconceptions and mishaps: Effective management of heart failure. Social Science and Medicine. 58, 631 – 643

Clark AM, Freydberg CN, McAlister FA, Tsuyuki RT, Armstrong PW, Strain LA (2009). Patient and informal caregivers’ knowledge of heart failure: necessary but insufficient for effective self-care. European Journal of Heart Failure. 11, 617 – 621.

Braunschweig F, Mortensen PT, Gras D, et al. (2005) Monitoring of physical activity and heart rate variability in patients with chronic heart failure using cardiac resynchronisation devices. American Journal of Cardiology. 95,1104 – 1107.

Adamson PB, Smith AL, Abraham WT et al (2004). Continuous autonomic assessment in patients with symptomatic heart failure. Circulation. 110, 2389 – 2394.

Yu CM, Wang L, Chau E et al. (2005) Intrathoracic impedance monitoring in patients with heart failure: correlation with fluid status and feasibility of early warning preceding hospitalization. Circulation. 112; 841-8.

Vollman D, Nagele H, Schauerte P et al.(2007) Clinical utility of intrathoracic impedance monitoring to alert patients with an implanted device of deteriorating chronic heart failure. European Heart Journal. 28; 1835-40.

Cowie MR, Conraads V, Tavazzi L, Yu CM, on behalf of the SENSE-HF Investigators (2009). Rationale and design of a prospective trial to assess the sensitivity and positive predictive value of implantable intrathoracic impedance monitoring in the prediction of heart failure hospitalizations: the SENSE-HF Study. Journal of Cardiac Failure 15; 394-400.

**Anticoagulation guidelines**

**Atrial fibrillation**

Fuster v et al. Guidelines for the management of patients with atrial fibrillation. Circulation 2006;114:e257-e354.

**Chads score**

Gage bf et al. Validation of clinical classification schemes for predicting stroke. Jama 2001:285:2864-2870.

Lip gyh. Refining clinical risk stratification. Chest 2009:10.1378.

**Appendix III.**

**Statistical Analysis Plan (SAP)**

**REM-HF RCT**

**Remote monitoring: an evaluation of implantable devices for management of heart failure patients**

**Date: 22/06/16**

**Final Version: 4.5**

**Prepared by:**

Scott Harris, Senior Medical Statistician

Dr Borislav D Dimitrov, Associate Professor in Medical Statistics

Professor James Raftery, Professor of Health Technology Assessment

**Edited and Approved by:**

Professor John Morgan, Co-Principle Investigator, University of Southampton

Professor Martin R Cowie, Co-Principle Investigator, Imperial College London

Professor Paul Roderick, University of Southampton

**Document history**

| **Version** | **Date** | **Changes made** |
| --- | --- | --- |
| 1.0 | 19/07/2015 | Original |
| 2.0 | 4/11/2015 | - |
| 3.0 | 6/12/2015 | - |
| 4.0 | 06/03/2016 | - |
| 4.1 | 01/04/2016 | Update to reflect final protocol and methodology paper |
| 4.2 | 03/05/2016 | Update to Statistical aspects |
| 4.3 | 23/05/2016 | Finalisation of Health Economics component and formatting changes |
| 4.4 | 09/06/2016 | Further formatting changes |
| 4.5 | 22/06/2016 | Final edits and formatting |

| **Document:** | | Statistical Analysis Plan (SAP) for Final Analysis | |
| --- | --- | --- | --- |
| **Sponsor Project Code:** | | REM-HF | |
| **UKCRN Study No.** | | 10383 | |
| **Study short title:** | | Remote monitoring in heart failure patients | |
| **Authors:** | | Scott Harris, Dr Borislav D Dimitrov, Prof. James Raftery,  Prof John Morgan, Prof Martin R Cowie, Prof Paul Roderick | |
| **Version date:** | | June 22^nd^ 2016 | |
| **Sponsor:** | | Southampton General Hospital, University Hospital Southampton NHS Foundation Trust | |
| **Senior Statistician:** | | Scott Harris, Senior Medical Statistician University of Southampton, Southampton, UK | |
| **Lead Health Economist:** | | Professor James Raftery University of Southampton, Southampton, UK | |
|  | |  |  |
| Confidential  The information contained herein is the property of University of Southampton and may not be reproduced, published or disclosed to others without written authorisation of the sponsor. The information provided in this document is strictly confidential and is available for review to investigators, potential investigators, health authorities and appropriate Ethics Committees. No disclosure should take place without written authorisation from the sponsor except to the extent necessary to obtain informed consent from potential subjects. Once signed, the terms of this protocol are binding for all. | |  |  |

**Table of Contents**

Signature Page 49

Abbreviations 50

Section 1. Introduction 51

1.1 Purpose of SAP 51

1.2 Trial Overview 51

1.2.1 Title 51

1.2.2 Trial Outline 51

1.2.3 Trial objectives 51

1.2.4 Participants 51

1.2.5 Intervention 51

1.2.6 Study Hypothesis 51

1.2.7 Recruiting sites 51

Section 2. Design Issues 52

2.1 Study Design 52

2.2 Randomisation procedure 52

2.3 Study Power and Sample Size 52

2.4 Data Collection, storage and management 53

2.4.1 Data Collection 53

2.4.2 Data Storage 53

2.4.3 Classification / confirmation of events 53

2.4.4 Data Management 53

2.5 Data monitoring and interim analyses 54

2.5.1 Planned Interim analyses 54

2.5.2 Rules for stopping the trial and adjustment of the significance level 54

Section 3. Statistical Analyses 54

3.1 Definition of Analysis Populations 54

3.1.1 Intention-to-treat population (ITT) 54

3.1.2 Per-Protocol population (PP) 54

3.2 Trial Endpoints 55

3.2.1 Primary Endpoint 55

3.2.2 Secondary Endpoints 55

3.3 Subject disposition 56

3.4 Demographic and Baseline Characteristics 56

3.5 Primary Analysis 56

3.6 Secondary Analyses 56

3.7 Extended Analyses 57

3.7.1 Inclusion of additional covariates 57

3.7.2 Proportion of remote monitoring for inclusion in per protocol Analysis 58

3.7.3 On-treatment (on-monitoring) analyses 58

3.8 Subgroup Analyses 58

3.9 Sensitivity Analyses 58

3.10 Missing Values 59

3.11 Analysis Software 59

Section 4. Health Economics Analysis 59

4.1 Measurements and elaboration 59

4.2 Process evaluation 59

4.3 QoL assessment 60

4.3.1 Transformation of skewed data 60

4.3.2 Missing data 60

4.4 Costs 60

4.4.1 Intervention cost 60

4.4.2 Resource use data 60

4.4.3 Medication Costs 61

4.4.4 Cost Analysis 62

4.4.5 Incremental Cost-Effectiveness Analysis 62

Section 5. Adverse events 62

5.1 Adverse event collection 62

5.2 Serious adverse event monitoring 62

5.3 SAE reporting 63

Section 6. List of tables and figures 63

6.1 Tables 63

6.1.1 Planned Baseline tables 63

6.1.2 Planned Outcome tables 63

6.2 Figures 65

Section 7. Appendices 66

7.1 ERC Charter – Version 7 66

7.2 Charter of the Data Safety Monitoring Board (DSMB) 69

7.3 Planned Baseline Tables 72

7.4 Planned Outcome Tables 80

7.5 Trial Timeline 83

7.6 Health economics: questionnaire and results (RMS) 84

7.6.1 Questionnaire sent to RMS 84

7.6.2 Results of RMS survey 85

Section 8. References 86

**Signature Page**

| **Co-Principal Investigator** | Prof. John Morgan  University of Southampton  Southampton, SO16 6YD, UK  E-mail: [jmm@hrclinic.org](mailto:jmm@hrclinic.org) | _______________  Signature | ______________  Date |
| --- | --- | --- | --- |
| **Co-Principal Investigator** | Prof. Martin Cowie  National Heart and Lung Institute  Imperial College London  Dovehouse Street  London, SW3 6LY, England  Tel.: +44-207-351 8856  Fax: +44-207-351-8164  E-mail: [m.cowie@imperial.ac.uk](mailto:m.cowie@imperial.ac.uk) | _______________  Signature | ______________  Date |
| **On behalf of the Sponsor** | Ailsa Duckworth  Head of Research & Development  University Hospital Southampton NHS Foundation Trust,  Southampton General Hospital,  Southampton, SO16 6YD, UK | _______________  Signature | ______________  Date |
| **Study Coordination/ CRO** | Sue Kitt  Study Manager  University Hospital Southampton NHS Foundation Trust,  Southampton General Hospital,  Southampton, SO16 6YD, UK | _______________  Signature | ______________  Date |
| **Senior Statistician** | Scott Harris  Senior Medical Statistician,  University of Southampton  Southampton, SO16 6YD, UK | _______________  Signature | ______________  Date |
| **Lead Health Economist** | Prof. James Raftery  University of Southampton  Southampton, SO16 6YD, UK | _______________  Signature | ______________  Date |

#

# Abbreviations

A&E Accident and Emergency

ACE Angiotensin-Converting Enzyme

ACM All-Cause Mortality

AE Adverse event

AHA American Heart Association

ARB Angiotensin Receptor Blockers

BHF British Heart Foundation

BMI Body Mass Index

BNF British National Formulary

CEA Cost Effectiveness Analysis

Centre Study centre; synonym for “site”

CRF Case report form

CRT-D Cardiac Resynchronisation Therapy-Defibrillator

CRT-P Cardiac Resynchronisation Therapy-Pacemaker

CV Cardiovascular

DSMB Data and Safety Monitoring Board

eCRF Electronic Case report Form

EQ5D Euroqol 5D

ERC Endpoint Review Committee

HEA Health Economics Analysis

HF Heart failure

HRG Healthcare Resource Group

ICD Implantable cardioverter defibrillator

IEC Independent Ethics Committee

IRB Institutional Review Board

KCCQ Kansas City Cardiomyopathy Questionnaire

LTAD Long Term Assist Device

LVEF Left ventricular ejection fraction

MI Myocardial infarction

MRA Mineralocorticoid Receptor Antagonists

NHS National Health Service

NICE National Institute for Health and Care Excellence

NYHA New York Heart Association

PPA Prescription Pricing Authority

PSSRU Personal Social Services Research Unit

QALY Quality Adjusted Life Year

QoL Quality of life

RCP Remote Care Pathway

REM Remote Event Monitoring

RMS Remote Monitoring Service

SAE Serious Adverse Event

SAP Statistical Analysis Plan

SC Steering Committee

Site Study site; (synonym for “centre”)

SOP Standard Operating Procedure

TSC Trial Steering Committee

UCP Usual Care Pathway

# Section 1. Introduction

## 1.1 Purpose of SAP

This Statistical Analysis Plan (SAP) is based on the final version of the study protocol (Version 19 - 12^th^ March 2014), the study design manuscript^1^ and the charters of the Endpoint Review Committee (ERC) and the Data and Safety Monitoring Board (DSMB) for the REM-HF trial. This SAP aims to further specify the procedures and statistical methods to be applied during the final analysis of the study data.

## 1.2 Trial Overview

### 1.2.1 Title

A randomised controlled trial of REmote Monitoring and evaluation of implantable devices for management of Heart Failure patients (REM-HF).

### 1.2.2 Trial Outline

This is a randomised, multi-centre, open label (non-blinded), parallel group, clinical trial in which patients are randomised to receive either the control/usual care (UCP) or intervention (RCP) pathways on a 1:1 basis. The randomisation is stratified on both Site and Device type (ICD, CRT-D or CRT-P). Recruitment started in September 2011, and the target accrual is 1650 randomised patients (825 per group) but with an event driven design (see 2.3). Follow-up is for a minimum of two years.

### 1.2.3 Trial objectives

To compare weekly remote monitoring-driven management (RCP) with usual care (UCP) for patients with heart failure, who have cardiac implanted electronic devices (ICD, CRT-D or CRT-P).

### **1.2.4 Participants**

Patients with heart failure who are implanted with an ICD, CRT-D or CRT-P device with remote monitoring capabilities. Patients must be on stable medical therapy for at least 6 weeks prior to recruitment and have had a device implant for at least 6 months.

### 1.2.5 Intervention

The remote care pathway (RCP) was informed by clinical experience and a review of the literature consisting of procedures to deal with heart failure, arrhythmia and device management. There will be weekly telemetering of data (downloads) and protocol-driven changes in care co-ordinated by the study site remote monitor. This care will be delivered through telephone support, primary care contact or direct patient contact.

### 1.2.6 Study Hypothesis

We hypothesise that patient-independent remote monitoring using implanted device technology and deployed in clinical pathways designed for chronic management of heart failure in the home, will significantly reduce all-cause patient mortality and cardiovascular hospitalisation in the context of NHS care and will be more cost effective than usual care.

### **1.2.7 Recruiting sites**

9 UK major cardiac centres that implant at least 200 ICDs/CRT-Ds per annum will recruit subjects to the trial. The 9 recruiting sites are (in alphabetical order):

- Blackpool – Blackpool Victoria Hospital, Blackpool Teaching Hospitals NHS Foundation Trust.
- Leeds – Leeds General Infirmary, Leeds Teaching Hospitals NHS Trust.
- Leicester – Glenfield Hospital, University Hospitals of Leicester NHS Trust.
- Liverpool – Liverpool Heart and Chest Hospital NHS Foundation Trust.
- London – Guys and St Thomas’ NHS Foundation Trust.
- London – Royal Brompton Hospital, Royal Brompton and Harefield NHS Foundation Trust.
- Manchester – Wythenshawe Hospital, University Hospital of South Manchester NHS Foundation Trust.
- Newcastle – Freeman Hospital, Newcastle upon Tyne NHS Foundation Trust.
- Southampton – Southampton General Hospital, University Hospital Southampton NHS Foundation Trust.

# Section 2. Design Issues

## 2.1 Study Design

This is a randomised, multi-centre, open label (non-blinded), parallel group, clinical trial in which patients are randomised to receive either the control (UCP) or intervention (RCP) pathways on a 1:1 basis.

## 2.2 Randomisation procedure

Subjects who met the inclusion criteria and did not meet any of the exclusion criteria were then given information about the trial. After giving informed consent each subject was then randomised to one of two management pathways; optimal medical management (UCP) or optimal medical management informed by weekly remote monitoring of the data from their implanted device (RCP) in a 1:1 ratio. Randomisation was performed centrally, by an external agency (Tenalea, The Netherlands) via a study-specific ECRF management system. The randomisation schedule was stratified by recruiting site and device type, with randomly permuted block sizes of either four or six patients per block.

## 2.3 Study Power and Sample Size

Our sample size calculation is driven by a required number of events and uses event rate estimates from previous studies: this is an event-driven trial. The UCP group event rate is estimated from the COMPANION^2^ and CARE-HF^3^ trials, that both had a patient population that we expect to be comparable to our study control group. These trials reported 48% for any death or CV disease hospitalisation at 2 years, and 39% at 29 months for CV death or unplanned hospitalisation, respectively. We anticipate slightly lower rates in this trial due to an improvement in routine care, and so assume a 40% event rate for our primary endpoint of all-cause mortality or unplanned CV hospitalisation (see 3.2.1) in the control arm at 2 years.

The meta-analysis of Clark et al^4^ that looked at telemonitoring or structured telephone support, reported a 20% risk reduction compared to usual care. Hence we assume that the intervention group will have an event rate of 32% at two years. This reduction in event rate (from 40% to 32%) at two years, if seen, would be clinically relevant.

Fixing a 5% (2-sided) significance level and 90% power, 546 events are needed to detect this effect size in a log-rank survival comparison of the two trial groups. To achieve this number of events we are likely to require 1394 patients in total (697 per group) with a minimum follow-up of 2 years. We will inflate this total by approximately 20% to allow for attrition, giving a final recruitment figure of 1650. This sample size would also be sufficient to provide a reasonable estimate of the effect size in the individual components of the primary outcome – ACM and CV disease related unplanned hospitalisation. This sample size is considered feasible within the trial timescales.

## 2.4 Data Collection, storage and management

### 2.4.1 Data Collection

An electronic clinical report form (eCRF) was utilised to capture healthcare utilisation elements, QoL assessment and clinical events on a patient-by-patient basis. This should allow for an accurate and comprehensive assessment of the care pathway. All aspects of local data recording and management are the responsibility of the remote monitor lead in each site.

All participants will complete the SF-12, EQ5D-3L and KCCQ at enrolment, 3 months, 6 months, 12 months and 24 months. These tools were administered by the study remote monitor leads in each centre. The initial health-related quality of life forms were completed at the time of patient enrolment in the hospital. Subsequent assessments were performed by the patients in their homes. The forms were sent to them by post with the necessary means for return of the forms. Receipt of the forms and their return were managed by the remote monitors and local study administration staff.

### 2.4.2 Data Storage

The original data are kept in each of the participating centres and conform to all necessary Data Protection Requirements. The data are also kept electronically on the TENALEA server, under the same European data protection regulations.

### 2.4.3 Classification / confirmation of events

All events suspected to be a primary or secondary outcome event will be adjudicated by two clinical experts on the Endpoint Review Committee (ERC). During this review each event will be classified according to the procedural rules of the ERC (see Appendix 1: 7.1).

### 2.4.4 Data Management

Prior to any statistical analysis, data will be exported by TENALEA as a series of SAS datasets. These SAS datasets will then be merged to produce the trial database. The trial database will be checked for missing, impossible and improbable values. Impossible and improbable values will be defined by clinical opinion/expertise. Improbable values will also include values that are outside three standard deviations of the mean value. Any questions regarding the data will go back to the trial data manager for possible referral back to the recruiting site. The final database will be locked for analysis once the ERC has classified all events and all identified data queries have been closed.

## 2.5 Data monitoring and interim analyses

### 2.5.1 Planned Interim analyses

An Independent DSMB reviewed recruitment, data completeness, primary outcome data, and safety of remote monitoring during the study. To facilitate the DSMB there was one planned interim analysis at 400 primary endpoint events (ACM or CV related hospitalisation) focusing on safety and the primary outcome. An independent statistician produced the analysis and presented the results to the DSMB. The independent statistician and the DSMB were not blinded to the treatment allocation.

### 2.5.2 Rules for stopping the trial and adjustment of the significance level

The DSMB had the ability to stop the trial early on safety grounds (see terms of reference for the DSMB – Appendix 2: 7.2) but in addition to this a formal stopping rule was instigated for the analysis of the primary outcome at 400 events. The threshold for significance at the time of the interim analysis was set at requiring a p value of <0.001. Setting the interim significance threshold at this level retained a threshold of p=0.048 for the final analysis using the Peto-Haybittle rule, whilst maintaining an overall 5% false positive error rate.

# Section 3. Statistical Analyses

## 3.1 Definition of Analysis Populations

### 3.1.1 Intention-to-treat population (ITT)

The primary analysis population is the Intention-To-Treat (ITT) population. The ITT population consists of all randomised subjects, with each subject analysed as part of the group to which they were randomised. All randomised participants will be included, including any subjects who are later found to be ineligible and those who do not follow the trial protocol.

### 3.1.2 Per-Protocol population (PP)

The PP population consists of all randomised patients who did not experience any major protocol violation within the first two years of follow up.

Protocol violations are any unapproved changes in the research study design and/or procedures that are within the investigator’s control and not in accordance with the IEC/IRB-approved protocol that may affect the participant’s rights, safety or well-being, or the completeness, accuracy and reliability of the study data. Classification of protocol violations will be done by an authorised core group of the TSC after review of a blinded list of protocol violations that occurred in the trial, but before the date of code break.

Protocol violations include enrolment of ineligible trial participants and application of study treatment other than by randomisation or other than as described in the study protocol.

Participants in the intervention arm (RCP) for whom we have less than 75% of their weekly remote monitoring downloads available (within the first two years) will be considered as major protocol violators. With regard to assessing compliance with data downloads, the weeks where a participant has been admitted to hospital (or is still a hospital inpatient) will be ignored - although remote monitoring may not occur during this period the patient will be being monitored locally.

The use of the 75% remote monitoring threshold as a definition of compliance with the protocol will be explored as one of the extended analyses.

## 3.2 Trial Endpoints

### 3.2.1 Primary Endpoint

The primary endpoint is the time to first event of either All-Cause Mortality (ACM) or unplanned Cardiovascular (CV) hospitalisation.

Cardiovascular mortality and unplanned cardiovascular hospitalisation will be evaluated by the Endpoint Review Committee (ERC), as outlined in Appendix 1: 7.1. Definitions will be documented in full in the procedural rules of the ERC. Death will be classified as cardiovascular unless an unequivocal non-cardiovascular cause of death has occurred. The extended analyses will include sensitivity analyses around the classifications.

### 3.2.2 Secondary Endpoints

The secondary endpoints of the trial are:

1. Time to death.
2. Time to a cardiovascular related death.
3. Time to a non-cardiovascular related death.
4. Time to first unplanned hospitalisation for cardiovascular reasons or cardiovascular related death.
5. Time to first unplanned hospitalisation for non-cardiovascular reasons or death by any cause.
6. Time to first unplanned hospitalisation for cardiovascular reasons.
7. Time to first unplanned hospitalisation for non-cardiovascular reasons.
8. Total number of unplanned hospitalisations.
9. Total number of unplanned hospitalisations for cardiovascular reasons.
10. Change from baseline in SF12 scores at 2 years (physical and mental health components).
11. Change from baseline in EQ5D scores at 2 years.
12. Change from baseline in KCCQ at 2 years.
13. Change from baseline in SF12 scores at 1 year (physical and mental health components).
14. Change from baseline in EQ5D scores at 1 year.
15. Change from baseline in KCCQ at 1 year.
16. Number and cost of hospitalisations.
17. Difference in cost of resources consumed.
18. Difference in cost of cardiovascular related health care use.
19. Incremental costs per quality-adjusted life years (QALYs).

Unless otherwise stated, secondary endpoints will be measured at the last follow-up or at the last available observation within the two year follow-up period.

## 3.3 Subject disposition

The screening process will be described and details of why subjects failed screening will be summarised with frequency counts. The number of subjects enrolled into the study and the number of subjects enrolled but not randomised, together with the reasons for not being randomised, will also be summarised.

The number of randomised subjects and the number of subjects withdrawing consent will be summarised by treatment group.

The frequency of subjects enrolled in each recruiting site will be tabulated by randomised treatment group and for all randomised subjects combined. This will also be presented by device type.

## 3.4 Demographic and Baseline Characteristics

All demographic and baseline characteristics will be summarised by randomised treatment group. The distribution of continuous variables will be examined by assessing treatment group specific histograms. Variables that appear to be normally distributed will be summarised using means, standard deviations, minimum and maximum values. Variables that do not appear to be normally distributed will additionally be summarised with medians, lower and upper quartiles. Categorical variables will be summarised using frequency counts and percentages within each of the randomised treatment groups.

## 3.5 Primary Analysis

The primary analysis will be performed on the adjudicated events in the ITT population.

The main analysis will use a Cox proportional hazards model to derive the hazard ratio for the comparison between UCP and RCP. The stratifying variables of recruiting site and device type will be included in the model as fixed effect categorical variables. All estimates will be reported as hazard ratios (HR) and will be presented with 95% confidence intervals.

Subjects will be censored at the time of study end for those alive and free of unplanned cardiovascular hospitalisation, and at the last date known to be alive and free of such hospitalisation for those who are lost to follow-up (e.g., those who leave a study site area).

Time to event will be visualised using Kaplan-Meier curves.

## 3.6 Secondary Analyses

Secondary clinical outcomes that are Time to Event will be analysed in a similar way to the primary endpoint. The analyses will be performed on adjudicated events in the ITT population. Subjects who are alive and event free at the end of the trial will be censored at the date of trial completion. Subjects who die (for a non-mortality endpoint) or who die for a different cause than the endpoint under investigation will be censored at their date of death. Those lost to follow-up will be censored at the date that their status was last known.

The total number of unplanned hospitalisations (for cardiovascular reasons and for all reasons) will be analysed using the Andersen-Gill model, a Cox regression model for counting processes. We will also explore the use of Poisson regression (with duration of follow-up included as an offset) and negative binomial models, depending on the range of counts that are seen in the data.

Continuous endpoints will be analysed using Analysis of Covariance (ANCOVA) including the baseline value as a covariate where available. Variables that appear to be skewed following a visual inspection of the separate group histograms will undergo a two parameter Box-Cox transformation prior to analysis.

Analysis approaches for the Health Economics endpoints are discussed in the Health Economics section (Section 4).

All secondary endpoint comparisons will be performed using the ITT population, and will include the same covariates as the model for the primary analysis (site and device type) and will be conducted using a 5% significance level. All estimates will be presented with 95% confidence intervals. There will be no imputation of missing values or adjustment of the significance level for multiplicity.

Time to event comparisons will be visualised using Kaplan-Meier curves.

## 3.7 Extended Analyses

The regression models fitted for the primary and secondary analyses will be extended through the exploration of the influence of the other baseline variables on the outcome and through on-treatment analyses that track each participant’s actual remote monitoring status. The impact of different thresholds of remote monitoring adherence for the inclusion in the PP population will also be explored. In addition, sensitivity analyses will be performed on the secondary endpoints for the CV death ‘equivalent’ events - emergency heart transplantation, appropriate shock from ICD, LTAD insertion and survived resuscitation of sudden cardiac arrest.

### 3.7.1 Inclusion of additional covariates

The primary and secondary analyses will be repeated, additionally including gender and baseline NYHA classification as fixed effect categorical variables and age at randomisation as a continuous covariate.

An additional set of exploratory analyses will examine the impact of further covariates from the baseline visit. For each of the primary and secondary analyses the impact of the following variables will be explored:

1. Diabetes:
   1. Type (none, type 1 or type 2).
   2. Treatment (insulin dependent or not).
2. Aetiology (Ischaemic or non-ischaemic heart failure).
3. Medication use (including beta-blockers, ACE/ARB, MRA, diuretics).
4. Body mass index (BMI).
5. Systolic blood pressure.
6. Pulse pressure.
7. LVEF.
8. Haemoglobin.

### 3.7.2 Proportion of remote monitoring for inclusion in per protocol Analysis

The threshold used for including participants in the PP analysis set, based on their percentage of remote monitoring that was conducted, will be explored. A range of different threshold percentages for successful monitoring will be used, with the impact on the treatment effect examined. This will enable us to explore whether the completeness of the monitoring has any impact on its effectiveness.

### 3.7.3 On-treatment (on-monitoring) analyses

The term on-treatment in this trial refers to the participants being actively, remotely monitored. Time periods where the actual remote monitoring status of the participants changes over time (for any given individual) will be analysed according to the actual status of the remote monitoring. In effect a patient who changes remote monitoring status will switch between the two treatment groups. This is achieved by the introduction of time-dependent treatment indicator variables. However, the fact that trial participants are switching between groups means that on-treatment analyses are no longer protected from bias by the randomisation process, and hence we fully acknowledge that they should be interpreted with caution.

## 3.8 Subgroup Analyses

The following subgroups have been defined prior to the randomisation code break:

- Age (<70 years, ≥70 years).
- Gender.
- NYHA (≤II vs. >II).
- Type of device (ICD, CRT-P and CRT-D)
- Ischaemic / non-ischaemic heart failure.
- Occurrence of shock therapy.

Target outcomes (the primary endpoint and selected secondary endpoints) will be presented by the given subgroups and compared using interaction tests. To facilitate this testing, an interaction term between the treatment comparison and the subgroup variable will be added to the relevant model for each of these subgroup analyses.

## 3.9 Sensitivity Analyses

The primary and secondary endpoints as well as the final models from the extended analyses will also be evaluated in the PP population, to investigate the sensitivity of the results. This sensitivity analysis will only be performed if the number of major protocol violations suggests that the main results may change.

The primary and secondary analyses will also be replicated using multiple imputation to assess the impact of the missing data values (where applicable).

In addition, sensitivity analyses will be performed on the secondary endpoints for the CV death ‘equivalent’ events - heart transplantation, appropriate shock from ICD, LTAD insertion and survived resuscitation of sudden cardiac arrest.

## 3.10 Missing Values

For the primary analysis no missing values will be replaced for the main analysis and so complete cases will be used (although there should be no missing values for the selected baseline covariates and the outcome is time to event, which allows for censored observations). Should there be any missing data then a supplementary sensitivity analysis that makes use of multiple imputation will be presented to assess the impact of the missing values.

For the secondary analyses complete case analyses will be used as the main analyses (although again the time to event outcomes allow for censored outcome data). In a similar fashion to the primary analysis multiple imputation will be used to assess the impact of any missing data as a supporting sensitivity analysis.

For the extended analyses multiple imputation will be used for the final models to assess the impact of any missing data on the conclusions.

## 3.11 Analysis Software

Data processing, elaboration, description and any additional coding or recoding, as well as all main analyses will be performed using the following routine licensed statistical software programmes:

1. SAS version 9.3 or later.
2. SPSS version 22 or later.
3. Stata version 12 or later.

All analyses and software scripts/logs will be stored in a shared directory on a secure server which can be accessed by the data manager, trial statistician and other study investigators.

# Section 4. Health Economics Analysis

## 4.1 Measurements and elaboration

The economic evaluation comprises a cost effectiveness analysis in terms of incremental cost per QALY. The composite nature of the primary outcome prevents its use as a measure of cost effectiveness. The perspective is that of the NHS.

In addition a process evaluation will compare the pattern of care between the two arms and across centres (usual care is not standardised and the protocol may be interpreted slightly differently by centre). Key abnormalities detected in remote monitoring relate to atrial/ventricular arrhythmias and HF events. Key interventions include changes in medication, including anti-coagulants for atrial fibrillation. Interventions in both groups will be assessed and compared.

.

## 4.2 Process evaluation

To the extent that remote monitoring in REM-HF leads to superior outcomes, it would do so by a process in which monitoring led to changes in treatment, mainly through medication changes (e.g., change in dose of diuretic, addition of new diuretic, anticoagulation for AF, etc.) and / or device re-setting. The process evaluation using data collected in the trial will measure the extent to which such changes in medication and in device re-sets occurred more frequently in the intervention compared to the control arm. The frequency of intervention by type would be compared with available data from other similar trials, with exploration, if relevant, of the implications for cost effectiveness.

## 4.3 QoL assessment

EQ5D data will be used with the UK tariff to estimate differences in QALY increments. Values will be interpolated between collection points and differences estimated using area under the curve.

### 4.3.1 Transformation of skewed data

Log-transformation or bootstrapping of any continuous data (e.g. direct costs), will be applied as required. If normality is not achieved, these data will be analysed by non-parametric analyses and tests.

The non-parametric method of bootstrapping, which makes no assumptions concerning the distribution of the underlying data will be applied to data on cost and quality of life.

### 4.3.2 Missing data

No imputation of missing data will be applied for the CEA data. Only patients with complete data will be included in the primary economic analysis, with imputation explored in sensitivity analyses.

## 4.4 Costs

The relevant costs are those of the intervention and of linked changes in the resources used related to HF. These include medications, devices, SAEs, any other HF related hospitalisations and use of community health services.

### 4.4.1 Intervention cost

The intervention, remote monitoring, has to do with the provision of the monitors in each of the 9 sites. This excludes the cost of the devices fitted as these would be fitted anyhow. The direct cost of the remote monitoring will be personnel, seven of which were HF nurses (band 7) and two physiologists. These personnel worked partly on providing the intervention but also partly as researchers. Their research roles included recruitment, consent and data recording. Their remote monitoring role had to do with receiving and checking the weekly patient downloads and recommending changes as required. These elements have to be separated with only the costs of the intervention being relevant to the economic evaluation. The relevant cost is what the intervention might cost if applied in routine practice.

To separate these aspects, the relevant person at each centre was asked to identify the total time spent on REM and to divide this between that devoted to the intervention and that to do with the research, at the start and in early 2015. The time devoted to providing the intervention was aggregated by centre and multiplied by the staff cost, based on a HF nurse band 7 including overheads.

### 4.4.2 Resource use data

Data relevant to the use of NHS resources were collected in five routine questionnaires headed:

1. Healthcare Utilisation Form,
2. System Modification,
3. SAE Hospitalisation,
4. Medication, and
5. Follow up interview.

These were administered at baseline, 3, 6 and 12 months and then yearly with the final collection at trial exit.

Levels of resource use by patient will be estimated under the following seven headings:

1. System modification (including reasons from a pre specified list)
2. CVD medication by 11 classes at start of study and new medication during the study with date. Other medication was recorded as yes/no.
3. Cardiac tests/procedures as day case admission
4. SAEs resulting in a hospitalisation, divided between those related to the study and not, along with duration of hospital stay
5. Hospital admissions (any other HF related hospitalisations other than SAEs)
6. Use of GP and practice nurses and Cardiac / HF Specialist nurses
7. Outpatient appointments and any A&E attendances.

Of these, 1) to 4) are plausibly related to the intervention while 5) to 7) refer to wider changes in health service use. For each of these items, the cost will be estimated as number of contacts multiplied by unit cost. The unit costs for GPs, practice nurses and Cardiac / HF nurses will be taken from PSSRU^5^. Hospital services costs, including for SAEs will be taken from National Reference costs. Drug costs by class will be taken from the BNF and PPA. The unit cost of system modifications will be based on that of an outpatient or day case contact as recorded in the trial. The cost of any A&E attendances will also be included.

### 4.4.3 Medication Costs

The REM medication questionnaire collected data on 11 classes of CVD related drugs, by name. Mean cost per day for each of the more commonly used drugs will be estimated, with class means used for those used rarely or lacking full data. The prices will be combined with recommended daily dose to provide a medications cost per patient in each arm.

Clinical opinion suggests that efficacy was most likely to apply to changed use of diuretics. Aldosterone antagonists also matter due to their potential effect on mortality. The levels of use of each of these classes of drug will be shown separately. As AF was defined as a subgroup for analysis, the relevant drugs (warfarin, NOACs) will also be identified and costed separately. Cost units for all drugs will be calculated based on BNF.

System modifications (to the implanted device) would normally require an outpatient or day-case attendance. These will be identified by attendance type and costed as standard attendances.

Serious adverse events (SAEs) requiring hospitalisation are identified in the SAE questionnaire. The number of hospital days in each arm for SAEs and any other relevant conditions will be multiplied by mean NHS national HRG cost. This will be expressed as cost per day by dividing by mean length of stay^6-7^.

### 4.4.4 Cost Analysis

Costs will be estimated as the sum of the costs of the intervention, hospitalisations, and medication. They will be calculated by multiplying measures of resource use time estimates by unit costs for each type of resource^6-7^. The description of the services provided to the patients or the medical procedures used for the initiation of treatment or for use of the device will be provided by each centre.

The costing year will be the last study year prior to data unblinding (2014/2015) - if cost data are not available for this year, inflation of previous cost years data will be performed.

QALYs can be estimated from both EQ5D and SF12, both of which were administered at baseline, in addition to 12 and 24 months (or at trial exit if less than 24 months). The primary analysis will use EQ5D with the SF12 used in a sensitivity analysis.EQ5D will be used to estimate QALYs via the UK valuation set. Utility scores will then be multiplied by the duration of time spent in that health state and total QALYs will then be calculated with the area under the curve method.^8-12^

Both QALYs and intervention costs will be estimated at patient level once the final dataset is made available. This will enable both to be included in the statistical analyses described above. This will be done by the trial statistician or by the health economic team as appropriate.

### 4.4.5 Incremental Cost-Effectiveness Analysis

The incremental differences in costs and effects will be compared between the intervention and control group and the incremental cost-effectiveness ratio (ICER) will be calculated using the formula: ICER = [costs (Intervention) – costs (control)] / [effects (intervention) – effects (control)].

Incremental costs and effects will be jointly bootstrapped and shown as a scatter plot on the cost effectiveness plane.

An ICER will be estimated for different levels of willingness to pay for a QALY using a cost effectiveness acceptability curve. Results will be compared with NICE’s threshold of £20,000 to £30,000.

The base case will be based on QALYs via EQ5D and NHS costs related to HF. Sensitivity analyses will include QALYs based on SF12 and on a range of monitoring costs based on different caseloads.

# Section 5. Adverse events

## 5.1 Adverse event collection

It is believed that there is no increased risk to REM-HF trial patients, as they will have access to currently available care pathways when required but will have the additional advantage of immediate but remote expert monitoring. However, the study will evaluate the acceptability of such monitoring to patients through any adverse impact on quality of life. Adverse events for this study are not being actively collected. The trial participants (or guardians of the trial participants) are however encouraged to report any serious adverse event by contacting the study team by telephone. They are also recommended to contact the GP or emergency service.

## 5.2 Serious adverse event monitoring

In agreement with the Sponsor of this study, all AEs that are classified within GCP as "serious" and that result in hospitalisation or death will be considered an SAE, documented in the ECRF and reviewed by an appropriately trained member of the research team. Where an SAE is assessed as being related to study procedures, the reporting will be escalated to the Sponsor and the concerned hosting R&D Department. SAEs not related to the study intervention will be documented in the eCRF but will not be escalated to the Sponsor or hosting R&D.

An Independent DSMB will review recruitment, data completeness, and endpoints, and to monitor any occurrence of serious adverse effects from remote monitoring, at appropriate time points during the study. Trial procedures also specify that the remote monitoring staff will review any available data in the light of an adverse event. The trial manager will also collate all protocol deviations and serious adverse event data reported by the remote monitors.

## 5.3 SAE reporting

All SAEs will be coded according to the latest version of MedDRA available. The data management team may wish to group MedDRA codes further into more clinically meaningful groups to aid interpretation.

All reported adverse events will be tabulated by treatment pathway. These will be separated into two tables: treatment related serious adverse events and serious adverse events that weren’t considered to be related to treatment. The number of participants who discontinue or who are withdrawn due to an SAE will also be tabulated.

# Section 6. List of tables and figures

A list of main planned tables and figures is shown below. Note, that this is an *a priori* table and figure listing. During statistical analysis, changes in the order and content of the tables and figures and to the structure of the tables and figures may be necessary. Additional tables and figures will also be produced to aid better exploration and understanding of the data.

## 6.1 Tables

### 6.1.1 Planned Baseline tables

Table 1: Demographic and baseline characteristics and medical history (ITT population)

Table 2a: Concomitant medication (ITT population).

Table 2b. Baseline Dosage of medication (ITT population).

Table 3: Baseline Laboratory values (ITT population).

Table 4a: Baseline EQ5D (ITT population).

Table 4b: Baseline SF-12 (ITT population).

Table 4c: Baseline KCCQ (ITT population).

### 6.1.2 Planned Outcome tables

Table 5: Hazard ratios for Primary and Secondary Time to Event Endpoints (ITT population).

Table 6: Relative risks for number of hospitalisations (ITT population).

Table 7a: Change in QoL scores from baseline to 2 years (ITT population).

Table 7b: Change in QoL scores from baseline to 1 year (ITT population).

Table 8: Health Economics Outcomes.

Tables 9 to 63: Replicates of Tables 5, 6, 7a, 7b and 8 in each of the 11 pre-specified subgroups (ITT population).

Tables 64 to 68: Replicates of Tables 5, 6, 7a, 7b and 8 (PP population). – Including Multiple Imputation results where applicable.

Table 69: Treatment related serious adverse events (ITT Population).

Table 70: Serious adverse reactions not considered related to treatment (ITT Population).

## 6.2 Figures

Figure 1: CONSORT Diagram.

Figure 2: Overview and time course of enrolment in REM-HF trial

Figure 3: Patient recruitment over time

Figure 4: Kaplan Meier: Time to first unplanned hospitalisation for cardiovascular reasons or death by any cause.

Figure 5: Kaplan Meier: Time to death.

Figure 6: Kaplan Meier: Time to a cardiovascular related death.

Figure 7: Kaplan Meier: Time to a non-cardiovascular related death.

Figure 8: Kaplan Meier: Time to first unplanned hospitalisation for cardiovascular reasons or cardiovascular related death.

Figure 9: Kaplan Meier: Time to first unplanned hospitalisation for non-cardiovascular reasons or death by any cause.

Figure 10: Kaplan Meier: Time to first unplanned hospitalisation for cardiovascular reasons.

Figure 11: Kaplan Meier: Time to first unplanned hospitalisation for non-cardiovascular reasons.

Figure 12: Health Economics Figure 1.

# Section 7.

## 7.1 ERC Charter – Version 7

**Definitions:**

**DEATH**

**General definitions**

*In case of hospitalisation resulting in death:* if death occurs within 24 hours after admission, or requires continuous life support measures from the time of admission until death,the primary label (= major reason) for the SAE is “death”. If death occurs more than 24 hours after admission, the primary label for the SAE is the reason as judged by the endpoint committee.

*Cardiovascular death:* Death will be classified as cardiovascular unless an unequivocal non-cardiovascular cause of death has occurred. Cardiovascular death includes sudden death, death due to MI, heart failure, or stroke; procedure-related death (cardiovascular investigation/procedure/operation); death due to other specified cardiovascular causes; and presumed cardiovascular deaths (i.e. those for which a non-cardiovascular cause had not been clearly established). Patients who undergo emergency heart transplantation due to end-stage heart failure will be counted as deaths. Patients who undergo elective heart transplantation will exit the study on the day of the transplant.

**Cardiovascular – Sudden (arrhythmic)**

Death within 24 hours of the onset of symptoms or unwitnessed death without any other obvious cause with confirmatory evidence from device interrogation where possible.

**Cardiovascular - Progressive Heart failure**

Death occurring with a history of progressive deterioration of symptoms and / or signs of Heart Failure in the absence of an unexpected cardiac arrest.

**Cardiovascular - Other (state cause)**

**Non-Cardiovascular**

**HOSPITALISATION**

**General definitions**

*“Hospitalisation”:* means admission to a hospital involving an overnight stay that involves a calendar date change or resulting in death. Day care admissions are not included. “Unplanned hospitalisation” also includes any prolongation of a hospitalisation based on another serious event.

*“Unplanned hospitalisation”:* any hospital stay overnight for major therapeutic intervention, tests or surgery that was triggered by symptoms, device malfunctions or other pathological findings e.g. laboratory values.

*“Planned hospitalisation”:* any overnight hospital stay that was scheduled, not as a result of symptoms, device malfunctions or pathological findings. Any hospitalisation that was scheduled before randomisation is counted as “planned” in the setting of the trial.

*Number of days in hospital:*  the number of midnights in hospital

**Unplanned Cardiovascular Hospitalisation for heart failure**

Unplanned hospitalisation due to heart failure or primarily for its treatment, or if heart failure was a major component of the patient’s hospital admission. A patient admitted for this reason must have symptoms and/or signs of worsening heart failure, ongoing or worsening in the prior 3 months, and been treated with additional oral or intravenous diuretics.

Evidence of worsening heart failure has to include at least one of the following items: increasing dyspnoea on exertion, orthopnoea, nocturnal dyspnoea or paroxysmal nocturnal dyspnoea, increasing fatigue or decreasing exercise tolerance, raised jugular venous pressure and/or increasing peripheral oedema, and/or radiological signs of pulmonary oedema or as judged by the ERC.

The following are examples fulfilling these admission criteria:

- clinical signs of heart failure recorded on admission with a chest infection
- symptomatic hypotension associated with HF medication
- significant metabolic / renal disturbance associated with heart failure or the medication for heart failure, with or without symptoms
- transplant assessment
- device upgrade to CRTD in the presence of heart failure symptoms or signs

**Unplanned Cardiovascular Hospitalisation but not for heart failure (state reason)**

The following are examples fulfilling these admission criteria:

- manifest vascular disease other than cardiac (cerebral, renal, peripheral)
- revision of system for lead / device malfunction or anticipated malfunction
- pre-syncope or syncope if no clinical signs of HF and no withdrawal of HF Meds = postural or reflex syncope
- acute coronary syndrome
- angina sounding chest pain and flow limiting coronary disease on angiography in the absence of a dynamic troponin rise
- appropriate or inappropriate ICD shocks or anti-tachycardia pacing

**Unplanned Hospitalisation for other reason (state reason)**

The following are examples fulfilling these admission criteria:

- cellulitis as a complication of oedema unless due to a direct vascular cause such as an ischaemic or diabetic ulcer
- venous ulcers
- metabolic / renal disturbance if no clinical signs of overt heart failure and no withdrawal of heart failure medication

**Planned Hospitalisation which should be removed as an endpoint (state reason)**

Conditions leading to an elective admission for a planned non-urgent reason. The following are examples fulfilling these admission criteria:.

- cardiovascular (generator change)
- non-cardiovascular (e.g. orthopaedic, plastic surgery or dental problems).

## 7.2 Charter of the Data Safety Monitoring Board (DSMB)

The purpose of the REM-HF Study is to evaluate whether remote monitoring of heart failure using implanted devices provides for reduction in mortality and hospitalisation as compared with the usual care. It is a randomised multicentre UK trial with parallel group design and patients are randomised one to one to either control (usual care) or remote monitor. The trial is funded by the British Heart Foundation and by an Industry Consortium.

1. The DSMB is a panel of expert clinical cardiologists and a medical statistician whose members have been appointed by the Steering Committee (SC) of the REM-HF Study. The DSMB monitors the execution of the REM-HF Study. The DSMB informs the SC regarding the safety of the patients and the feasibility of the study. For this purpose appropriate data will be provided by the REM-HF Study Manager from the Southampton REM-HF Clinical Trial Unit. The DSMB consists of 3 members with voting power and these are;

- Professor Henry Dargie (Chair)
- Professor Ian Ford
- Dr Richard Charles

In addition non-voting participants can be admitted at the discretion of the chairman of the committee and these are;

- REM-HF Study Manager or the responsible statistician or other visitors participating on an invitation of the DSMB Chairman.

2. The members of the DSMB will meet initially after the start of the REM-HF Study, after the interim analysis as defined in the study protocol (that is after approximately 400 events), at the end of the study, and at any time on special demand by the majority of the SC or DSMB members. The meetings are organised by the Study Manager on demand of the DSMB chairman and can be either a formal meeting or a teleconference. The chairman of the DSMB leads the meeting.

All members of the DSMB are obliged to advise the trial Steering Committee using their medical and scientific experience and competence with regard to the safety of the patients and the feasibility of the study, within the rules of this DSMB charter, which will be approved by the SC.

The attendance of each DSMB member (either in person or by telephone) is requested but in exceptional circumstances one member can provide written comments having reviewed the information, subject to the approval of the DSMB Chair. The substitution of a DSMB member by another person is not permitted.

3. The DSMB obtains access to unblinded data via the Study Manager. The DSMB may request access to additional data collected within the trial.

The tasks of the DSMB members are:

- To receive and comment on the study recruitments data by centre and by month
- To receive and comment on baseline data by randomization group at 6 monthly intervals to ensure that there was reasonable balance in key factors. (like the usual table 1 in an RCT paper)
- To receive and comment on data completeness: key variables and all questionnaires needed to be logged so it is clear what proportion of forms are complete/present and if there are any gaps that can be addressed (such as missing data from patient notes, or if there are processes that may need to be be changed to improve matters)
- To review the safety of patients, and the feasibility of the study, based on data items documented in the electronic case record forms and based on administrative data
- To inform the SC immediately about the DSMB opinion of essential changes of the protocol, or about the recommendation to stop the study in case of safety, efficacy or feasibility concerns
- The DSMB Chair will provide a formal report after each meeting of the DSMB to the SC, stating whether the opinion of the DSMB is that the trial continue or be terminated, and with the reasons for this.

The tasks of the REM-HF Study Manager are to:

- To prepare reports as defined by the DSMB
- To present reports at DSMB meetings
- To assure that all unblinded data and reports will be provided to DSMB voting

members, only

- To organise and manage DSMB face-to-face meetings, and telephone conferences if necessary
- To prepare meeting minutes for review by DSMB voting members.

4. The DSMB is entitled to postpone a decision and to require additional information or to request a joint meeting with the SC if this is necessary to arrive at a recommendation. Major issues of the DSMB members will be presented to the chairpersons of the SC by the chairman of the DSMB and then to all SC members, if requested by the SC chairpersons.

5. DSMB meetings or teleconferences are considered as having a quorum if at minimum two of the three voting members are present. Decisions will be made concordantly. The transfer of voting right to other persons in case of absence is not possible, neither to representatives of one's own institution, nor to other members of DSMB. Additional comments may be provided with the vote.

6. The participants of a DSMB meeting obtain reimbursement of their travel expenses. DSMB members with voting power obtain an allowance additionally. Details are agreed between the DSMB members and Southampton REM-HF Clinical Trial Unit..

Signatures

__________________________________ ___________________

Date

__________________________________ ___________________

Date

__________________________________ ___________________

Date

## 7.3 Planned Baseline Tables

| **Table 1.** Demographics, baseline characteristics and medical history (ITT population).  Figures are N (%) unless stated. | | | | |
| --- | --- | --- | --- | --- |
| **Variable** | | **Treatment Pathway** | |  |
|  |  | **RCP N=XXXX** | **UCP N=XXXX** | **Total N=XXXX** |
| **Age at randomisation**  (N=XXXX) | Mean (SD) | XX.X (XX.XX) | XX.X (XX.XX) | XX.X (XX.XX) |
|  | Median | XX.X | XX.X | XX.X |
|  | LQ to UQ | XX.X to XX.X | XX.X to XX.X | XX.X to XX.X |
|  | Min to Max | XX.X to XX.X | XX.X to XX.X | XX.X to XX.X |
| **Age at randomisation - Females**  (N=XXXX) | Mean (SD) | XX.X (XX.XX) | XX.X (XX.XX) | XX.X (XX.XX) |
|  | Median | XX.X | XX.X | XX.X |
|  | LQ to UQ | XX.X to XX.X | XX.X to XX.X | XX.X to XX.X |
|  | Min to Max | XX.X to XX.X | XX.X to XX.X | XX.X to XX.X |
| **Age at randomisation - Males**  (N=XXXX) | Mean (SD) | XX.X (XX.XX) | XX.X (XX.XX) | XX.X (XX.XX) |
|  | Median | XX.X | XX.X | XX.X |
|  | LQ to UQ | XX.X to XX.X | XX.X to XX.X | XX.X to XX.X |
|  | Min to Max | XX.X to XX.X | XX.X to XX.X | XX.X to XX.X |
| **Gender**  (N=XXXX) | Female | XXX (XX.X%) | XXX (XX.X%) | XXX (XX.X%) |
|  | Male | XXX (XX.X%) | XXX (XX.X%) | XXX (XX.X%) |
| **Body Mass Index (BMI)**  (N=XXXX) | Mean (SD) | XX.X (XX.XX) | XX.X (XX.XX) | XX.X (XX.XX) |
|  | Median | XX.X | XX.X | XX.X |
|  | LQ to UQ | XX.X to XX.X | XX.X to XX.X | XX.X to XX.X |
|  | Min to Max | XX.X to XX.X | XX.X to XX.X | XX.X to XX.X |
| **Body Mass Index (BMI) - Females**  (N=XXXX) | Mean (SD) | XX.X (XX.XX) | XX.X (XX.XX) | XX.X (XX.XX) |
|  | Median | XX.X | XX.X | XX.X |
|  | LQ to UQ | XX.X to XX.X | XX.X to XX.X | XX.X to XX.X |
|  | Min to Max | XX.X to XX.X | XX.X to XX.X | XX.X to XX.X |
| **Body Mass Index (BMI) - Males**  (N=XXXX) | Mean (SD) | XX.X (XX.XX) | XX.X (XX.XX) | XX.X (XX.XX) |
|  | Median | XX.X | XX.X | XX.X |
|  | LQ to UQ | XX.X to XX.X | XX.X to XX.X | XX.X to XX.X |
|  | Min to Max | XX.X to XX.X | XX.X to XX.X | XX.X to XX.X |
| **Recruiting Site**  (N=XXXX) | Blackpool | XXX (XX.X%) | XXX (XX.X%) | XXX (XX.X%) |
|  | Brompton | XXX (XX.X%) | XXX (XX.X%) | XXX (XX.X%) |
|  | Guys | XXX (XX.X%) | XXX (XX.X%) | XXX (XX.X%) |
|  | Leeds | XXX (XX.X%) | XXX (XX.X%) | XXX (XX.X%) |
|  | Leicester | XXX (XX.X%) | XXX (XX.X%) | XXX (XX.X%) |
|  | Liverpool | XXX (XX.X%) | XXX (XX.X%) | XXX (XX.X%) |
|  | Manchester | XXX (XX.X%) | XXX (XX.X%) | XXX (XX.X%) |
|  | Newcastle | XXX (XX.X%) | XXX (XX.X%) | XXX (XX.X%) |
|  | Southampton | XXX (XX.X%) | XXX (XX.X%) | XXX (XX.X%) |
| **NYHA Classification**  (N=XXXX) | I | XXX (XX.X%) | XXX (XX.X%) | XXX (XX.X%) |
|  | II | XXX (XX.X%) | XXX (XX.X%) | XXX (XX.X%) |
|  | III | XXX (XX.X%) | XXX (XX.X%) | XXX (XX.X%) |
|  | IV | XXX (XX.X%) | XXX (XX.X%) | XXX (XX.X%) |
| **Coronary Artery Disease**  (N=XXXX) | None | XXX (XX.X%) | XXX (XX.X%) | XXX (XX.X%) |
|  | Supposed | XXX (XX.X%) | XXX (XX.X%) | XXX (XX.X%) |
|  | Yes, documented, no previous intervention | XXX (XX.X%) | XXX (XX.X%) | XXX (XX.X%) |
|  | Yes, documented, previous intervention | XXX (XX.X%) | XXX (XX.X%) | XXX (XX.X%) |
| **Diabetes**  (N=XXXX) | None | XXX (XX.X%) | XXX (XX.X%) | XXX (XX.X%) |
|  | Type I | XXX (XX.X%) | XXX (XX.X%) | XXX (XX.X%) |
|  | Type II | XXX (XX.X%) | XXX (XX.X%) | XXX (XX.X%) |
| **Type II Diabetics on medication**  (N=XXXX) | No | XXX (XX.X%) | XXX (XX.X%) | XXX (XX.X%) |
|  | Yes | XXX (XX.X%) | XXX (XX.X%) | XXX (XX.X%) |
| **Systolic blood pressure**  (N=XXXX) | Mean (SD) | XX.X (XX.XX) | XX.X (XX.XX) | XX.X (XX.XX) |
|  | Median | XX.X | XX.X | XX.X |
|  | LQ to UQ | XX.X to XX.X | XX.X to XX.X | XX.X to XX.X |
|  | Min to Max | XX.X to XX.X | XX.X to XX.X | XX.X to XX.X |
| **Diastolic blood pressure**  (N=XXXX) | Mean (SD) | XX.X (XX.XX) | XX.X (XX.XX) | XX.X (XX.XX) |
|  | Median | XX.X | XX.X | XX.X |
|  | LQ to UQ | XX.X to XX.X | XX.X to XX.X | XX.X to XX.X |
|  | Min to Max | XX.X to XX.X | XX.X to XX.X | XX.X to XX.X |
| **Type of device**  (N=XXXX) | ICD | XXX (XX.X%) | XXX (XX.X%) | XXX (XX.X%) |
|  | CRT-D | XXX (XX.X%) | XXX (XX.X%) | XXX (XX.X%) |
|  | CRT-P | XXX (XX.X%) | XXX (XX.X%) | XXX (XX.X%) |
| **Pulse pressure**  (N=XXXX) | Mean (SD) | XX.X (XX.XX) | XX.X (XX.XX) | XX.X (XX.XX) |
|  | Median | XX.X | XX.X | XX.X |
|  | LQ to UQ | XX.X to XX.X | XX.X to XX.X | XX.X to XX.X |
|  | Min to Max | XX.X to XX.X | XX.X to XX.X | XX.X to XX.X |
| **Atrial Fibrilation**  (N=XXXX) | No | XXX (XX.X%) | XXX (XX.X%) | XXX (XX.X%) |
|  | Yes | XXX (XX.X%) | XXX (XX.X%) | XXX (XX.X%) |
| **Haemaglobin**  (N=XXXX) | Mean (SD) | XX.X (XX.XX) | XX.X (XX.XX) | XX.X (XX.XX) |
|  | Median | XX.X | XX.X | XX.X |
|  | LQ to UQ | XX.X to XX.X | XX.X to XX.X | XX.X to XX.X |
|  | Min to Max | XX.X to XX.X | XX.X to XX.X | XX.X to XX.X |
| **Heart rate**  (N=XXXX) | Mean (SD) | XX.X (XX.XX) | XX.X (XX.XX) | XX.X (XX.XX) |
|  | Median | XX.X | XX.X | XX.X |
|  | LQ to UQ | XX.X to XX.X | XX.X to XX.X | XX.X to XX.X |
|  | Min to Max | XX.X to XX.X | XX.X to XX.X | XX.X to XX.X |
| **Ischaemic Heart Failure**  (N=XXXX) | No | XXX (XX.X%) | XXX (XX.X%) | XXX (XX.X%) |
|  | Yes | XXX (XX.X%) | XXX (XX.X%) | XXX (XX.X%) |
| **LVEF in %**  (N=XXXX) | Mean (SD) | XX.X (XX.XX) | XX.X (XX.XX) | XX.X (XX.XX) |
|  | Median | XX.X | XX.X | XX.X |
|  | LQ to UQ | XX.X to XX.X | XX.X to XX.X | XX.X to XX.X |
|  | Min to Max | XX.X to XX.X | XX.X to XX.X | XX.X to XX.X |

| **Table 2a.** Concomitant medication (ITT population). Figures are N (%). | | | | |
| --- | --- | --- | --- | --- |
| **Variable** | | **Treatment Pathway** | |  |
|  |  | **RCP N=XXXX** | **UCP N=XXXX** | **Total N=XXXX** |
| **ACE Inhibitors**  (N=XXXX) | No | XXX (XX.X%) | XXX (XX.X%) | XXX (XX.X%) |
|  | Yes | XXX (XX.X%) | XXX (XX.X%) | XXX (XX.X%) |
| **ARB**  (N=XXXX) | No | XXX (XX.X%) | XXX (XX.X%) | XXX (XX.X%) |
|  | Yes | XXX (XX.X%) | XXX (XX.X%) | XXX (XX.X%) |
| **Aldosterone antagonists**  (N=XXXX) | No | XXX (XX.X%) | XXX (XX.X%) | XXX (XX.X%) |
|  | Yes | XXX (XX.X%) | XXX (XX.X%) | XXX (XX.X%) |
| **Antiarrhythmics**  (N=XXXX) | No | XXX (XX.X%) | XXX (XX.X%) | XXX (XX.X%) |
|  | Yes | XXX (XX.X%) | XXX (XX.X%) | XXX (XX.X%) |
| **Anticoagulants**  (N=XXXX) | No | XXX (XX.X%) | XXX (XX.X%) | XXX (XX.X%) |
|  | Yes | XXX (XX.X%) | XXX (XX.X%) | XXX (XX.X%) |
| **Antidepressants**  (N=XXXX) | No | XXX (XX.X%) | XXX (XX.X%) | XXX (XX.X%) |
|  | Yes | XXX (XX.X%) | XXX (XX.X%) | XXX (XX.X%) |
| **Antidiabetics**  (N=XXXX) | No | XXX (XX.X%) | XXX (XX.X%) | XXX (XX.X%) |
|  | Yes | XXX (XX.X%) | XXX (XX.X%) | XXX (XX.X%) |
| **Beta blockers**  (N=XXXX) | No | XXX (XX.X%) | XXX (XX.X%) | XXX (XX.X%) |
|  | Yes | XXX (XX.X%) | XXX (XX.X%) | XXX (XX.X%) |
| **Calcium antagonists**  (N=XXXX) | No | XXX (XX.X%) | XXX (XX.X%) | XXX (XX.X%) |
|  | Yes | XXX (XX.X%) | XXX (XX.X%) | XXX (XX.X%) |
| **Cardiac Glycosides**  (N=XXXX) | No | XXX (XX.X%) | XXX (XX.X%) | XXX (XX.X%) |
|  | Yes | XXX (XX.X%) | XXX (XX.X%) | XXX (XX.X%) |
| **Coronary Medication**  (N=XXXX) | No | XXX (XX.X%) | XXX (XX.X%) | XXX (XX.X%) |
|  | Yes | XXX (XX.X%) | XXX (XX.X%) | XXX (XX.X%) |
| **Diuretics**  (N=XXXX) | No | XXX (XX.X%) | XXX (XX.X%) | XXX (XX.X%) |
|  | Yes | XXX (XX.X%) | XXX (XX.X%) | XXX (XX.X%) |
| **Loop diuretics**  (N=XXXX) | No | XXX (XX.X%) | XXX (XX.X%) | XXX (XX.X%) |
|  | Yes | XXX (XX.X%) | XXX (XX.X%) | XXX (XX.X%) |
| **Nitrates**  (N=XXXX) | No | XXX (XX.X%) | XXX (XX.X%) | XXX (XX.X%) |
|  | Yes | XXX (XX.X%) | XXX (XX.X%) | XXX (XX.X%) |
| **Psychotropic drugs**  (N=XXXX) | No | XXX (XX.X%) | XXX (XX.X%) | XXX (XX.X%) |
|  | Yes | XXX (XX.X%) | XXX (XX.X%) | XXX (XX.X%) |
| **Renin Inhibitors**  (N=XXXX) | No | XXX (XX.X%) | XXX (XX.X%) | XXX (XX.X%) |
|  | Yes | XXX (XX.X%) | XXX (XX.X%) | XXX (XX.X%) |
| **Vasodilators**  (N=XXXX) | No | XXX (XX.X%) | XXX (XX.X%) | XXX (XX.X%) |
|  | Yes | XXX (XX.X%) | XXX (XX.X%) | XXX (XX.X%) |

| **Table 2b.** Baseline Dosage of medication (ITT population). | | | | |
| --- | --- | --- | --- | --- |
| **Variable** | | **Treatment Pathway** | |  |
|  |  | **RCP N=XXXX** | **UCP N=XXXX** | **Total N=XXXX** |
| **Bisoprolol**  (N=XXXX) | Mean (SD) | XX.X (XX.XX) | XX.X (XX.XX) | XX.X (XX.XX) |
|  | Median | XX.X | XX.X | XX.X |
|  | LQ to UQ | XX.X to XX.X | XX.X to XX.X | XX.X to XX.X |
|  | Min to Max | XX.X to XX.X | XX.X to XX.X | XX.X to XX.X |
| **Metoprolol**  (N=XXXX) | Mean (SD) | XX.X (XX.XX) | XX.X (XX.XX) | XX.X (XX.XX) |
|  | Median | XX.X | XX.X | XX.X |
|  | LQ to UQ | XX.X to XX.X | XX.X to XX.X | XX.X to XX.X |
|  | Min to Max | XX.X to XX.X | XX.X to XX.X | XX.X to XX.X |
| **Carvedilol**  (N=XXXX) | Mean (SD) | XX.X (XX.XX) | XX.X (XX.XX) | XX.X (XX.XX) |
|  | Median | XX.X | XX.X | XX.X |
|  | LQ to UQ | XX.X to XX.X | XX.X to XX.X | XX.X to XX.X |
|  | Min to Max | XX.X to XX.X | XX.X to XX.X | XX.X to XX.X |
| **Nebivolol**  (N=XXXX) | Mean (SD) | XX.X (XX.XX) | XX.X (XX.XX) | XX.X (XX.XX) |
|  | Median | XX.X | XX.X | XX.X |
|  | LQ to UQ | XX.X to XX.X | XX.X to XX.X | XX.X to XX.X |
|  | Min to Max | XX.X to XX.X | XX.X to XX.X | XX.X to XX.X |
| **Atenolol**  (N=XXXX) | Mean (SD) | XX.X (XX.XX) | XX.X (XX.XX) | XX.X (XX.XX) |
|  | Median | XX.X | XX.X | XX.X |
|  | LQ to UQ | XX.X to XX.X | XX.X to XX.X | XX.X to XX.X |
|  | Min to Max | XX.X to XX.X | XX.X to XX.X | XX.X to XX.X |
| **Ramipril**  (N=XXXX) | Mean (SD) | XX.X (XX.XX) | XX.X (XX.XX) | XX.X (XX.XX) |
|  | Median | XX.X | XX.X | XX.X |
|  | LQ to UQ | XX.X to XX.X | XX.X to XX.X | XX.X to XX.X |
|  | Min to Max | XX.X to XX.X | XX.X to XX.X | XX.X to XX.X |
| **Enalapril**  (N=XXXX) | Mean (SD) | XX.X (XX.XX) | XX.X (XX.XX) | XX.X (XX.XX) |
|  | Median | XX.X | XX.X | XX.X |
|  | LQ to UQ | XX.X to XX.X | XX.X to XX.X | XX.X to XX.X |
|  | Min to Max | XX.X to XX.X | XX.X to XX.X | XX.X to XX.X |
| **Perindopril**  (N=XXXX) | Mean (SD) | XX.X (XX.XX) | XX.X (XX.XX) | XX.X (XX.XX) |
|  | Median | XX.X | XX.X | XX.X |
|  | LQ to UQ | XX.X to XX.X | XX.X to XX.X | XX.X to XX.X |
|  | Min to Max | XX.X to XX.X | XX.X to XX.X | XX.X to XX.X |
| **Lisinopril** (N=XXXX) | Mean (SD) | XX.X (XX.XX) | XX.X (XX.XX) | XX.X (XX.XX) |
|  | Median | XX.X | XX.X | XX.X |
|  | LQ to UQ | XX.X to XX.X | XX.X to XX.X | XX.X to XX.X |
|  | Min to Max | XX.X to XX.X | XX.X to XX.X | XX.X to XX.X |
| **Candesartan**  (N=XXXX) | Mean (SD) | XX.X (XX.XX) | XX.X (XX.XX) | XX.X (XX.XX) |
|  | Median | XX.X | XX.X | XX.X |
|  | LQ to UQ | XX.X to XX.X | XX.X to XX.X | XX.X to XX.X |
|  | Min to Max | XX.X to XX.X | XX.X to XX.X | XX.X to XX.X |
| **Valsartan**  (N=XXXX) | Mean (SD) | XX.X (XX.XX) | XX.X (XX.XX) | XX.X (XX.XX) |
|  | Median | XX.X | XX.X | XX.X |
|  | LQ to UQ | XX.X to XX.X | XX.X to XX.X | XX.X to XX.X |
|  | Min to Max | XX.X to XX.X | XX.X to XX.X | XX.X to XX.X |
| **Losartan**  (N=XXXX) | Mean (SD) | XX.X (XX.XX) | XX.X (XX.XX) | XX.X (XX.XX) |
|  | Median | XX.X | XX.X | XX.X |
|  | LQ to UQ | XX.X to XX.X | XX.X to XX.X | XX.X to XX.X |
|  | Min to Max | XX.X to XX.X | XX.X to XX.X | XX.X to XX.X |
| **Irbesartan**  (N=XXXX) | Mean (SD) | XX.X (XX.XX) | XX.X (XX.XX) | XX.X (XX.XX) |
|  | Median | XX.X | XX.X | XX.X |
|  | LQ to UQ | XX.X to XX.X | XX.X to XX.X | XX.X to XX.X |
|  | Min to Max | XX.X to XX.X | XX.X to XX.X | XX.X to XX.X |
| **Olmesartan**  (N=XXXX) | Mean (SD) | XX.X (XX.XX) | XX.X (XX.XX) | XX.X (XX.XX) |
|  | Median | XX.X | XX.X | XX.X |
|  | LQ to UQ | XX.X to XX.X | XX.X to XX.X | XX.X to XX.X |
|  | Min to Max | XX.X to XX.X | XX.X to XX.X | XX.X to XX.X |
| **All diuretics**  (N=XXXX) | Mean (SD) | XX.X (XX.XX) | XX.X (XX.XX) | XX.X (XX.XX) |
|  | Median | XX.X | XX.X | XX.X |
|  | LQ to UQ | XX.X to XX.X | XX.X to XX.X | XX.X to XX.X |
|  | Min to Max | XX.X to XX.X | XX.X to XX.X | XX.X to XX.X |
| **Loop diuretics**  (N=XXXX) | Mean (SD) | XX.X (XX.XX) | XX.X (XX.XX) | XX.X (XX.XX) |
|  | Median | XX.X | XX.X | XX.X |
|  | LQ to UQ | XX.X to XX.X | XX.X to XX.X | XX.X to XX.X |
|  | Min to Max | XX.X to XX.X | XX.X to XX.X | XX.X to XX.X |

| **Table 3.** Baseline Laboratory values (ITT population). Figures are N (%) unless stated. | | | | |
| --- | --- | --- | --- | --- |
| **Variable** | | **Treatment Pathway** | |  |
|  |  | **RCP N=XXXX** | **UCP N=XXXX** | **Total N=XXXX** |
| **Haemoglobin (g/dl)**  (N=XXXX) | Mean (SD) | XX.X (XX.XX) | XX.X (XX.XX) | XX.X (XX.XX) |
|  | Median | XX.X | XX.X | XX.X |
|  | LQ to UQ | XX.X to XX.X | XX.X to XX.X | XX.X to XX.X |
|  | Min to Max | XX.X to XX.X | XX.X to XX.X | XX.X to XX.X |
| **Creatinine (umol/l)**  (N=XXXX) | Mean (SD) | XX.X (XX.XX) | XX.X (XX.XX) | XX.X (XX.XX) |
|  | Median | XX.X | XX.X | XX.X |
|  | LQ to UQ | XX.X to XX.X | XX.X to XX.X | XX.X to XX.X |
|  | Min to Max | XX.X to XX.X | XX.X to XX.X | XX.X to XX.X |
| **eGFR (ml/min/1.73m^2^)**  (N=XXXX) | Mean (SD) | XX.X (XX.XX) | XX.X (XX.XX) | XX.X (XX.XX) |
|  | Median | XX.X | XX.X | XX.X |
|  | LQ to UQ | XX.X to XX.X | XX.X to XX.X | XX.X to XX.X |
|  | Min to Max | XX.X to XX.X | XX.X to XX.X | XX.X to XX.X |
| **Leucocytes**  (N=XXXX) | Mean (SD) | XX.X (XX.XX) | XX.X (XX.XX) | XX.X (XX.XX) |
|  | Median | XX.X | XX.X | XX.X |
|  | LQ to UQ | XX.X to XX.X | XX.X to XX.X | XX.X to XX.X |
|  | Min to Max | XX.X to XX.X | XX.X to XX.X | XX.X to XX.X |
| **Haematocrit**  (N=XXXX) | Mean (SD) | XX.X (XX.XX) | XX.X (XX.XX) | XX.X (XX.XX) |
|  | Median | XX.X | XX.X | XX.X |
|  | LQ to UQ | XX.X to XX.X | XX.X to XX.X | XX.X to XX.X |
|  | Min to Max | XX.X to XX.X | XX.X to XX.X | XX.X to XX.X |
| **B-type natriuretic peptide (pg/ml)** (N=XXXX) | Mean (SD) | XX.X (XX.XX) | XX.X (XX.XX) | XX.X (XX.XX) |
|  | Median | XX.X | XX.X | XX.X |
|  | LQ to UQ | XX.X to XX.X | XX.X to XX.X | XX.X to XX.X |
|  | Min to Max | XX.X to XX.X | XX.X to XX.X | XX.X to XX.X |

| **Table 4a.** Baseline EQ5D (ITT population). Figures are N (%) unless stated. | | | | |
| --- | --- | --- | --- | --- |
| **Variable** | | **Treatment Pathway** | |  |
|  |  | **RCP N=XXXX** | **UCP N=XXXX** | **Total N=XXXX** |
| **Mobility**  (N=XXXX) | I have no problem walking around | XXX (XX.X%) | XXX (XX.X%) | XXX (XX.X%) |
|  | I have some difficulty walking around | XXX (XX.X%) | XXX (XX.X%) | XXX (XX.X%) |
|  | I have to stay in bed | XXX (XX.X%) | XXX (XX.X%) | XXX (XX.X%) |
| **To look after oneself**  (N=XXXX) | I have no difficulty looking after myself | XXX (XX.X%) | XXX (XX.X%) | XXX (XX.X%) |
|  | I have some difficulty washing and getting dressed | XXX (XX.X%) | XXX (XX.X%) | XXX (XX.X%) |
|  | I am not able to wash or get dressed unaided | XXX (XX.X%) | XXX (XX.X%) | XXX (XX.X%) |
| **Daily activities (e.g. work, studies, housework, family- or leisure activities)** (N=XXXX) | I have no difficulty going about my daily activities | XXX (XX.X%) | XXX (XX.X%) | XXX (XX.X%) |
|  | I have some difficulty going about my daily activities | XXX (XX.X%) | XXX (XX.X%) | XXX (XX.X%) |
|  | I am not able to go about my daily activities | XXX (XX.X%) | XXX (XX.X%) | XXX (XX.X%) |
| **Pain, physical disorders** (N=XXXX) | I have no pain or discomfort | XXX (XX.X%) | XXX (XX.X%) | XXX (XX.X%) |
|  | I have moderate pain or discomfort | XXX (XX.X%) | XXX (XX.X%) | XXX (XX.X%) |
|  | I have severe pain or discomfort | XXX (XX.X%) | XXX (XX.X%) | XXX (XX.X%) |
| **Fear, depression**  (N=XXXX) | I am not anxious or depressed | XXX (XX.X%) | XXX (XX.X%) | XXX (XX.X%) |
|  | I am moderately anxious or depressed | XXX (XX.X%) | XXX (XX.X%) | XXX (XX.X%) |
|  | I am extremely anxious or depressed | XXX (XX.X%) | XXX (XX.X%) | XXX (XX.X%) |
| **State of health rated on a scale of 1 (very poor) to 100 (very good)** (N=XXXX) | Mean (SD) | XX.X (XX.XX) | XX.X (XX.XX) | XX.X (XX.XX) |
|  | Median | XX.X | XX.X | XX.X |
|  | LQ to UQ | XX.X to XX.X | XX.X to XX.X | XX.X to XX.X |
|  | Min to Max | XX.X to XX.X | XX.X to XX.X | XX.X to XX.X |
| **Total score**  (N=XXXX) | Mean (SD) | XX.X (XX.XX) | XX.X (XX.XX) | XX.X (XX.XX) |
|  | Median | XX.X | XX.X | XX.X |
|  | LQ to UQ | XX.X to XX.X | XX.X to XX.X | XX.X to XX.X |
|  | Min to Max | XX.X to XX.X | XX.X to XX.X | XX.X to XX.X |

| **Table 4b.** Baseline SF-12 (ITT population). | | | | |
| --- | --- | --- | --- | --- |
| **Variable** | | **Treatment Pathway** | |  |
|  |  | **RCP N=XXXX** | **UCP N=XXXX** | **Total N=XXXX** |
| **Physical Functioning (PF)**  (N=XXXX) | Mean (SD) | XX.X (XX.XX) | XX.X (XX.XX) | XX.X (XX.XX) |
|  | Median | XX.X | XX.X | XX.X |
|  | LQ to UQ | XX.X to XX.X | XX.X to XX.X | XX.X to XX.X |
|  | Min to Max | XX.X to XX.X | XX.X to XX.X | XX.X to XX.X |
| **Role Physical (RF)**  (N=XXXX) | Mean (SD) | XX.X (XX.XX) | XX.X (XX.XX) | XX.X (XX.XX) |
|  | Median | XX.X | XX.X | XX.X |
|  | LQ to UQ | XX.X to XX.X | XX.X to XX.X | XX.X to XX.X |
|  | Min to Max | XX.X to XX.X | XX.X to XX.X | XX.X to XX.X |
| **Role Emotional (RE)**  (N=XXXX) | Mean (SD) | XX.X (XX.XX) | XX.X (XX.XX) | XX.X (XX.XX) |
|  | Median | XX.X | XX.X | XX.X |
|  | LQ to UQ | XX.X to XX.X | XX.X to XX.X | XX.X to XX.X |
|  | Min to Max | XX.X to XX.X | XX.X to XX.X | XX.X to XX.X |
| **Mental Health (MH)**  (N=XXXX) | Mean (SD) | XX.X (XX.XX) | XX.X (XX.XX) | XX.X (XX.XX) |
|  | Median | XX.X | XX.X | XX.X |
|  | LQ to UQ | XX.X to XX.X | XX.X to XX.X | XX.X to XX.X |
|  | Min to Max | XX.X to XX.X | XX.X to XX.X | XX.X to XX.X |
| **Bodily Pain (BP)**  (N=XXXX) | Mean (SD) | XX.X (XX.XX) | XX.X (XX.XX) | XX.X (XX.XX) |
|  | Median | XX.X | XX.X | XX.X |
|  | LQ to UQ | XX.X to XX.X | XX.X to XX.X | XX.X to XX.X |
|  | Min to Max | XX.X to XX.X | XX.X to XX.X | XX.X to XX.X |
| **General Health (GH)**  (N=XXXX) | Mean (SD) | XX.X (XX.XX) | XX.X (XX.XX) | XX.X (XX.XX) |
|  | Median | XX.X | XX.X | XX.X |
|  | LQ to UQ | XX.X to XX.X | XX.X to XX.X | XX.X to XX.X |
|  | Min to Max | XX.X to XX.X | XX.X to XX.X | XX.X to XX.X |
| **Vitality (VT)**  (N=XXXX) | Mean (SD) | XX.X (XX.XX) | XX.X (XX.XX) | XX.X (XX.XX) |
|  | Median | XX.X | XX.X | XX.X |
|  | LQ to UQ | XX.X to XX.X | XX.X to XX.X | XX.X to XX.X |
|  | Min to Max | XX.X to XX.X | XX.X to XX.X | XX.X to XX.X |
| **Social Functioning (SF)**  (N=XXXX) | Mean (SD) | XX.X (XX.XX) | XX.X (XX.XX) | XX.X (XX.XX) |
|  | Median | XX.X | XX.X | XX.X |
|  | LQ to UQ | XX.X to XX.X | XX.X to XX.X | XX.X to XX.X |
|  | Min to Max | XX.X to XX.X | XX.X to XX.X | XX.X to XX.X |
| **Physical Component Summary (PCS) score**  (N=XXXX) | Mean (SD) | XX.X (XX.XX) | XX.X (XX.XX) | XX.X (XX.XX) |
|  | Median | XX.X | XX.X | XX.X |
|  | LQ to UQ | XX.X to XX.X | XX.X to XX.X | XX.X to XX.X |
|  | Min to Max | XX.X to XX.X | XX.X to XX.X | XX.X to XX.X |
| **Mental Component Summary (MCS) score**  (N=XXXX) | Mean (SD) | XX.X (XX.XX) | XX.X (XX.XX) | XX.X (XX.XX) |
|  | Median | XX.X | XX.X | XX.X |
|  | LQ to UQ | XX.X to XX.X | XX.X to XX.X | XX.X to XX.X |
|  | Min to Max | XX.X to XX.X | XX.X to XX.X | XX.X to XX.X |
| **Total score**  (N=XXXX) | Mean (SD) | XX.X (XX.XX) | XX.X (XX.XX) | XX.X (XX.XX) |
|  | Median | XX.X | XX.X | XX.X |
|  | LQ to UQ | XX.X to XX.X | XX.X to XX.X | XX.X to XX.X |
|  | Min to Max | XX.X to XX.X | XX.X to XX.X | XX.X to XX.X |

| **Table 4c.** Baseline KCCQ (ITT population). | | | | |
| --- | --- | --- | --- | --- |
| **Variable** | | **Treatment Pathway** | |  |
|  |  | **RCP N=XXXX** | **UCP N=XXXX** | **Total N=XXXX** |
| **Total Score**  (N=XXXX) | Mean (SD) | XX.X (XX.XX) | XX.X (XX.XX) | XX.X (XX.XX) |
|  | Median | XX.X | XX.X | XX.X |
|  | LQ to UQ | XX.X to XX.X | XX.X to XX.X | XX.X to XX.X |
|  | Min to Max | XX.X to XX.X | XX.X to XX.X | XX.X to XX.X |
| **Physical Limitation score**  (N=XXXX) | Mean (SD) | XX.X (XX.XX) | XX.X (XX.XX) | XX.X (XX.XX) |
|  | Median | XX.X | XX.X | XX.X |
|  | LQ to UQ | XX.X to XX.X | XX.X to XX.X | XX.X to XX.X |
|  | Min to Max | XX.X to XX.X | XX.X to XX.X | XX.X to XX.X |
| **Symptom Frequency score**  (N=XXXX) | Mean (SD) | XX.X (XX.XX) | XX.X (XX.XX) | XX.X (XX.XX) |
|  | Median | XX.X | XX.X | XX.X |
|  | LQ to UQ | XX.X to XX.X | XX.X to XX.X | XX.X to XX.X |
|  | Min to Max | XX.X to XX.X | XX.X to XX.X | XX.X to XX.X |
| **Symptom burden score**  (N=XXXX) | Mean (SD) | XX.X (XX.XX) | XX.X (XX.XX) | XX.X (XX.XX) |
|  | Median | XX.X | XX.X | XX.X |
|  | LQ to UQ | XX.X to XX.X | XX.X to XX.X | XX.X to XX.X |
|  | Min to Max | XX.X to XX.X | XX.X to XX.X | XX.X to XX.X |
| **Total symptom score**  (N=XXXX) | Mean (SD) | XX.X (XX.XX) | XX.X (XX.XX) | XX.X (XX.XX) |
|  | Median | XX.X | XX.X | XX.X |
|  | LQ to UQ | XX.X to XX.X | XX.X to XX.X | XX.X to XX.X |
|  | Min to Max | XX.X to XX.X | XX.X to XX.X | XX.X to XX.X |
| **Self efficacy score**  (N=XXXX) | Mean (SD) | XX.X (XX.XX) | XX.X (XX.XX) | XX.X (XX.XX) |
|  | Median | XX.X | XX.X | XX.X |
|  | LQ to UQ | XX.X to XX.X | XX.X to XX.X | XX.X to XX.X |
|  | Min to Max | XX.X to XX.X | XX.X to XX.X | XX.X to XX.X |
| **Quality of life score**  (N=XXXX) | Mean (SD) | XX.X (XX.XX) | XX.X (XX.XX) | XX.X (XX.XX) |
|  | Median | XX.X | XX.X | XX.X |
|  | LQ to UQ | XX.X to XX.X | XX.X to XX.X | XX.X to XX.X |
|  | Min to Max | XX.X to XX.X | XX.X to XX.X | XX.X to XX.X |
| **Social limitation score**  (N=XXXX) | Mean (SD) | XX.X (XX.XX) | XX.X (XX.XX) | XX.X (XX.XX) |
|  | Median | XX.X | XX.X | XX.X |
|  | LQ to UQ | XX.X to XX.X | XX.X to XX.X | XX.X to XX.X |
|  | Min to Max | XX.X to XX.X | XX.X to XX.X | XX.X to XX.X |
| **Overall summary score**  (N=XXXX) | Mean (SD) | XX.X (XX.XX) | XX.X (XX.XX) | XX.X (XX.XX) |
|  | Median | XX.X | XX.X | XX.X |
|  | LQ to UQ | XX.X to XX.X | XX.X to XX.X | XX.X to XX.X |
|  | Min to Max | XX.X to XX.X | XX.X to XX.X | XX.X to XX.X |
| **Clinical summary score**  (N=XXXX) | Mean (SD) | XX.X (XX.XX) | XX.X (XX.XX) | XX.X (XX.XX) |
|  | Median | XX.X | XX.X | XX.X |
|  | LQ to UQ | XX.X to XX.X | XX.X to XX.X | XX.X to XX.X |
|  | Min to Max | XX.X to XX.X | XX.X to XX.X | XX.X to XX.X |

## 7.4 Planned Outcome Tables

| **Table 5.** Cox regression models for Primary and Secondary Time-to-Event endpoints.(ITT population) | | | | | | |
| --- | --- | --- | --- | --- | --- | --- |
| **Outcome** | **Adjusted for Site and Device type** | | | **Adjusted for Site and Device type, Age, Gender and NYHC** | | |
|  | **Hazard Ratio^✝^** | **95% CI** | **P value** | **Hazard Ratio^✝^** | **95% CI** | **P value** |
| **Death or first unplanned hospitalisation for CV reasons (Primary)** | X.XX | X.XX to X.XX | X.XXX | X.XX | X.XX to X.XX | X.XXX |
| **All cause mortality^*^** | X.XX | X.XX to X.XX | X.XXX | X.XX | X.XX to X.XX | X.XXX |
| **CV related mortality^*^** | X.XX | X.XX to X.XX | X.XXX | X.XX | X.XX to X.XX | X.XXX |
| **Non-CV related mortality** | X.XX | X.XX to X.XX | X.XXX | X.XX | X.XX to X.XX | X.XXX |
| **CV related death or first unplanned hospitalisation for CV reasons** | X.XX | X.XX to X.XX | X.XXX | X.XX | X.XX to X.XX | X.XXX |
| **Death or first unplanned hospitalisation for non-CV reasons** | X.XX | X.XX to X.XX | X.XXX | X.XX | X.XX to X.XX | X.XXX |
| **First unplanned hospitalisation for CV reasons** | X.XX | X.XX to X.XX | X.XXX | X.XX | X.XX to X.XX | X.XXX |
| **First unplanned hospitalisation for non-CV reasons** | X.XX | X.XX to X.XX | X.XXX | X.XX | X.XX to X.XX | X.XXX |

✝ Hazard ratio calculated with the UCP group set as the reference.

* Sensitivity analyses and listings will be performed around ACM and CV mortality including (and not including) CV death equivalents (heart transplantation, appropriate shock from ICD, LTAD insertion and survived resuscitation of sudden cardiac arrest)

| **Table 6.** Poisson regression models for secondary count outcomes (follow-up time included as an offset). (ITT population) | | | | | | |
| --- | --- | --- | --- | --- | --- | --- |
| **Outcome** | **Adjusted for Site and Device type** | | | **Adjusted for Site, Device type, Age, Gender and NYHC** | | |
|  | **Relative Risk^✝^** | **95% CI** | **P value** | **Relative Risk^✝^** | **95% CI** | **P value** |
| **Total number of unplanned hospitalisations** | X.XX | X.XX to X.XX | X.XXX | X.XX | X.XX to X.XX | X.XXX |
| **All cause mortality** | X.XX | X.XX to X.XX | X.XXX | X.XX | X.XX to X.XX | X.XXX |

✝ Relative risk calculated with the UCP group set as the reference.

| **Table 7a.** Quality of Life Endpoints at 2 years. (ITT population) | | | | | | | |
| --- | --- | --- | --- | --- | --- | --- | --- |
| **Outcome** | | **Adjusted for Site, Device type and Baseline score** | | | **Adjusted for Site, Device type, Age, Gender, NYHC and Baseline score** | | |
|  |  | **Difference^✝^** | **95% CI** | **P value** | **Difference^✝^** | **95% CI** | **P value** |
| **EQ-5D** | Visual Analogue | XX.XX | XX.XX to XX.XX | X.XXX | XX.XX | XX.XX to XX.XX | X.XXX |
|  | Total | XX.XX | XX.XX to XX.XX | X.XXX | XX.XX | XX.XX to XX.XX | X.XXX |
| **SF-12** | Physical Component Summary (PCS) | XX.XX | XX.XX to XX.XX | X.XXX | XX.XX | XX.XX to XX.XX | X.XXX |
|  | Mental Component Summary (MCS) | XX.XX | XX.XX to XX.XX | X.XXX | XX.XX | XX.XX to XX.XX | X.XXX |
|  | Total | XX.XX | XX.XX to XX.XX | X.XXX | XX.XX | XX.XX to XX.XX | X.XXX |
| **KCCQ** | Physical Limitation | XX.XX | XX.XX to XX.XX | X.XXX | XX.XX | XX.XX to XX.XX | X.XXX |
|  | Symptom Frequency | XX.XX | XX.XX to XX.XX | X.XXX | XX.XX | XX.XX to XX.XX | X.XXX |
|  | Symptom burden | XX.XX | XX.XX to XX.XX | X.XXX | XX.XX | XX.XX to XX.XX | X.XXX |
|  | Total symptom | XX.XX | XX.XX to XX.XX | X.XXX | XX.XX | XX.XX to XX.XX | X.XXX |
|  | Self-efficacy | XX.XX | XX.XX to XX.XX | X.XXX | XX.XX | XX.XX to XX.XX | X.XXX |
|  | Quality of life | XX.XX | XX.XX to XX.XX | X.XXX | XX.XX | XX.XX to XX.XX | X.XXX |
|  | Social limitation | XX.XX | XX.XX to XX.XX | X.XXX | XX.XX | XX.XX to XX.XX | X.XXX |
|  | Overall summary | XX.XX | XX.XX to XX.XX | X.XXX | XX.XX | XX.XX to XX.XX | X.XXX |
|  | Clinical summary | XX.XX | XX.XX to XX.XX | X.XXX | XX.XX | XX.XX to XX.XX | X.XXX |
|  | Total | XX.XX | XX.XX to XX.XX | X.XXX | XX.XX | XX.XX to XX.XX | X.XXX |

✝ Difference calculated with the UCP group as the reference.

| **Table 7b.** Quality of Life Endpoints at 1 year. (ITT population) | | | | | | | |
| --- | --- | --- | --- | --- | --- | --- | --- |
| **Outcome** | | **Adjusted for Site, Device type and Baseline score** | | | **Adjusted for Site, Device type, Age, Gender, NYHC and Baseline score** | | |
|  |  | **Difference^✝^** | **95% CI** | **P value** | **Difference^✝^** | **95% CI** | **P value** |
| **EQ-5D** | Visual Analogue | XX.XX | XX.XX to XX.XX | X.XXX | XX.XX | XX.XX to XX.XX | X.XXX |
|  | Total | XX.XX | XX.XX to XX.XX | X.XXX | XX.XX | XX.XX to XX.XX | X.XXX |
| **SF-12** | Physical Component Summary (PCS) | XX.XX | XX.XX to XX.XX | X.XXX | XX.XX | XX.XX to XX.XX | X.XXX |
|  | Mental Component Summary (MCS) | XX.XX | XX.XX to XX.XX | X.XXX | XX.XX | XX.XX to XX.XX | X.XXX |
|  | Total | XX.XX | XX.XX to XX.XX | X.XXX | XX.XX | XX.XX to XX.XX | X.XXX |
| **KCCQ** | Physical Limitation | XX.XX | XX.XX to XX.XX | X.XXX | XX.XX | XX.XX to XX.XX | X.XXX |
|  | Symptom Frequency | XX.XX | XX.XX to XX.XX | X.XXX | XX.XX | XX.XX to XX.XX | X.XXX |
|  | Symptom burden | XX.XX | XX.XX to XX.XX | X.XXX | XX.XX | XX.XX to XX.XX | X.XXX |
|  | Total symptom | XX.XX | XX.XX to XX.XX | X.XXX | XX.XX | XX.XX to XX.XX | X.XXX |
|  | Self-efficacy | XX.XX | XX.XX to XX.XX | X.XXX | XX.XX | XX.XX to XX.XX | X.XXX |
|  | Quality of life | XX.XX | XX.XX to XX.XX | X.XXX | XX.XX | XX.XX to XX.XX | X.XXX |
|  | Social limitation | XX.XX | XX.XX to XX.XX | X.XXX | XX.XX | XX.XX to XX.XX | X.XXX |
|  | Overall summary | XX.XX | XX.XX to XX.XX | X.XXX | XX.XX | XX.XX to XX.XX | X.XXX |
|  | Clinical summary | XX.XX | XX.XX to XX.XX | X.XXX | XX.XX | XX.XX to XX.XX | X.XXX |
|  | Total | XX.XX | XX.XX to XX.XX | X.XXX | XX.XX | XX.XX to XX.XX | X.XXX |

✝ Difference calculated with the UCP group as the reference.

## 7.5 Trial Timeline

## 7.6 Health economics: questionnaire and results (RMS)

### 7.6.1 Questionnaire sent to RMS

Please estimate the average number of hours per week the remote monitor role at your site is spending in each of the following three activity groups, both during the first year if you were employed at that time and again now. Please put a whole number in here not a range. It is just your estimate.

| **Site name:** | **Year 1** | **Year 3** |
| --- | --- | --- |
| **Download specific:**  *Reviewing the downloads*  *Initiating interventions as a result of the downloads* |  |  |
| **Research Specific Activity:**  *Screening patients*  *Randomisation and baseline visits*  *Research paperwork- GP information sheets, health questionnaires*  *ECRF data entry*  *Reporting SAEs and collecting source data*  *Follow up visits* |  |  |
| **Other:**  Any other activity or tasks such as other duties/mandatory training /meetings etc |  |  |

Thank you for completing this.

### 7.6.2 Results of RMS survey

| Year 1 | Newcastle | Guys | Liverpool | Blackpool | Leicester | Leeds | Soothampton | Brompton | Manchester |  |  |
| --- | --- | --- | --- | --- | --- | --- | --- | --- | --- | --- | --- |
| Monitoring | 18 | - | 12 | 14 | 10 | 12 | 6 | 10 | - | 12 | 31 |
| Research | 18 | - | 25 | 20 | 28 | 35 | 25 | 12 | - | 23 |  |
| Other | 2 | - | 1 | 3 | 5 | 2 | 4 | 1 | - | 2 |  |
| total | 38 |  | 38 | 37 | 43 | 48 | 35 | 23 |  | 37 |  |
| Year 2 |  |  |  |  |  |  |  |  |  |  |  |
| Monitoring | 12 | 22 | 10 | 10 | 15 | 17 | 12 | 6 | 10 | 13 | 39 |
| Research | 18 | 15 | 27 | 22 | 21 | 16 | 20 | 5 | 15 | 18 |  |
| Other | 2 | 1 | 1 | 5 | 2 | 1 | 4 | 1 | 3 | 2 |  |
| total | 32 | 38 | 38 | 37 | 38 | 33 | 36 | 12 | 28 | 32 | 39 |

Of the 9 centres, 7 replied for year one, and all in year two. These replies put the mean number of hours in year one at 37, with 12 devoted to monitoring. Figures for year two were 32 and 13 hours per week, respectively.

For costing, the mean number of hours per week in each centre devoted to monitoring will be 12 in year one and 13 in year two.

# Section 8. References

1. Morgan JM, Dimitrov BD, Gill J, Kitt S, Ng GA, McComb JM, Raftery J, Roderick P, Seed A, Williams SG, Witte KK, Wright DJ, Yao GL and Cowie MR. Rationale and study design of the REM-HF study: remote management of heart failure using implanted devices and formalised follow-up procedures. *European Journal for Heart Failure* 2014; 16(9): 1039-1045.
2. Bristow MR, Saxon LA, Boehmer J, et al. Cardiac-resynchronization therapy with or without an implantable defibrillator in advanced chronic heart failure. *N Engl J Med* 2004; 350: 2140-2150.
3. Cleland JG, Daubert JC, Erdmann E, Freemantle N, Gras D, Kappenberger L, Tavazzi L; Cardiac Resynchronization-Heart Failure (CARE-HF) Study Investigators. The effect of cardiac resynchronization on morbidity and mortality in heart failure. *N Engl J Med* 2005; 352: 1539-49.
4. Clark RA, Inglis SC, McAlister FA, et al Telemonitoring or structured telephone support programmes for patients with chronic heart failure: systematic review and meta-analysis. *British Medical Journal* 2007; 334: 910-1.
5. Curtis L, Burns A. *Unit Costs of Health and Social Care 2015*. Personal Social Services Research Unit. Available from:

<http://www.pssru.ac.uk/project-pages/unit-costs/2015/> [Accessed: 19^th^ May 2016].

1. Department of Health. *NHS Reference costs 2012 to 2013*. Available from:

<https://www.gov.uk/government/publications/nhs-reference-costs-2012-to-2013>

[Accessed: 19^th^ May 2016].

1. Department of Health. *Payment by Results in the NHS: tariff for 2013 to 2014*. Available from: <https://www.gov.uk/government/collections/payment-by-results-2013-14>

[Accessed: 19^th^ May 2016].

1. The EuroQol Group. EQ-5D-3L User Guide - Basic information on how to use the EQ-5D-3L instrument. Available from: <http://www.euroqol.org/about-eq-5d/publications/user-guide.html> [Accessed: 20^th^ May 2016]
2. Lothgren M, Zethraeus N. Definition, interpretation and calculation of cost-effectiveness acceptability curves. *Health Econ* 2000; 9(7):623-630.
3. National Institute of Health and Care Excellence (NICE). Guide to the methods of technology appraisal 2013. Availe from: <https://www.nice.org.uk/article/pmg9/chapter/Foreword> [Accessed 20th May 2016]
4. Whitehead SJ, Ali S. Health outcomes in Economic Evaluation: the QALY and utilities. *Br Med Bull* 2010; 96: 5-21.
5. Zethraeus N, Johannesson M, Jonsson B, Lothgren M, Tambour M. Advantages of using the net-benefit approach for analysing uncertainty in economic evaluation studies. *Pharmacoeconomics* 2003; 21(1):39-48.
